# Integrative genomic and single-cell transcriptomic analyses suggest a putative zinc transport–immune axis in Meniere’s disease

**DOI:** 10.3389/fneur.2026.1858821

**Published:** 2026-09-08

**Authors:** Yanping Yu, Jiancheng Xue, Zhuohao Li, Tao Chen, Rizhao Liu, Hongsong Dong, Beiping Miao, Guohui Nie

**Affiliations:** 1 Department of Otolaryngology Head & Neck Surgery, The Second People’s Hospital of Shenzhen, The First Affiliated Hospital of Shenzhen University, Shenzhen, China; 2 Dongguan Key Laboratory of Environmental Medicine, The First Dongguan Affiliated Hospital, School of Public Health, Guangdong Medical University, Dongguan, Guangdong, China

**Keywords:** *GAB1*, immune-cell remodeling, Mendelian randomization (MR), Meniere’s disease, single-cell RNA (scRNA) sequencing, *SLC39A10*, *XCL2*, zinc homeostasis

## Abstract

**Objective:**

Meniere’s disease is a disabling inner-ear disorder whose molecular basis remains poorly understood. Immune dysregulation and genetic susceptibility have been implicated in disease etiology, but the genes and immune-cell programs linking genetic susceptibility to disease-associated molecular alterations remain incompletely defined. Focusing on zinc transport and immune signaling, we aimed to prioritize genetically supported candidate genes and identify the immune-cell programs in which they are embedded.

**Methods:**

We combined bulk peripheral blood transcriptomes, single-cell RNA sequencing of peripheral blood mononuclear cells, large-scale blood eQTL data, and genome-wide association summary statistics for Meniere’s disease. Differential expression analysis and WGCNA were used to define bulk transcriptional signatures, which were then integrated with scRNA-seq-derived cell-type marker genes to generate a candidate-gene set with complementary bulk disease-association and cell-type localization evidence. Two-sample Mendelian randomization and colocalization were applied to test whether genetically predicted expression of these candidate genes influences Meniere’s disease risk. Prioritized genes were then mapped to immune-cell subsets, ligand–receptor communication networks, and CD4^+^ T-cell differentiation trajectories at single-cell resolution, and their expression changes were validated at the mRNA and protein levels in an independent clinical cohort.

**Results:**

Bulk and single-cell analyses yielded 202 Meniere’s disease-associated candidate genes enriched in RNA processing, chromatin regulation, and immune-related pathways. Two-sample Mendelian randomization identified nominal associations between genetically predicted expression of *SLC39A10, GAB1, and XCL2* and Meniere’s disease risk. Integration with bulk transcriptomic evidence prioritized increased *SLC39A10* expression and reduced *GAB1* and *XCL2* expression for further analysis. Immune-signature enrichment and single-cell analyses localized their expression-related programs to CD4^+^ T cells, NK cells, monocytes, B cells, and other peripheral immune populations. CellChat and pseudotime analyses provided complementary descriptions of inferred intercellular communication and CD4^+^ T-cell state transitions. In an independent clinical cohort, *SLC39A10, GAB1*, and *XCL2* showed concordant changes at the transcript and protein levels.

**Conclusion:**

Our multi-layered genomic and single-cell analyses prioritize *SLC39A10*, *GAB1*, and *XCL2* as candidate genes associated with Meniere’s disease and map their expression-related programs to CD4^+^ T-cell, NK-cell, and other peripheral immune compartments. Together, these findings support a putative zinc transport–immune framework that may contribute to systemic immune dysregulation in Meniere’s disease. This framework should be regarded as a hypothesis generated by integrative genomic and transcriptomic evidence and requires direct validation through measurements of zinc homeostasis, transporter activity, and downstream immune function.

## 1 Introduction

Meniere’s disease (MD) is a chronic inner-ear disorder characterized by recurrent spontaneous vertigo, fluctuating sensorineural hearing loss, tinnitus, and aural fullness (1–3). Beyond these cardinal symptoms, Meniere’s disease imposes a substantial psychosocial and socioeconomic burden because unpredictable attacks and progressive auditory decline severely impair daily functioning and quality of life. Histopathologically, endolymphatic hydrops is a hallmark lesion, yet it is not known how systemic and local factors converge on endolymphatic dysregulation and neuroepithelial injury (4, 5). Current treatments, from dietary modification and diuretics to intratympanic steroids, gentamicin and ablative surgery, are largely empirical and only partially effective for many patients, a condition that underscores the need for a mechanistic framework to guide rational biomarker development and targeted interventions (1, 2).

Clinical and experimental evidence point to immune and inflammatory contributions to Meniere’s disease (6–9). Co-occurrence of Meniere’s disease with systemic autoimmune diseases, detection of circulating autoantibodies and immune complexes, and partial responsiveness to corticosteroids in subsets of patients all suggest an immune-mediated component (7–10). Importantly, Meniere’s disease is clinically and immunologically heterogeneous and is unlikely to represent a single inflammatory entity. Previous studies have proposed distinct inflammatory, autoinflammatory, and autoimmune-associated endotypes characterized by differences in cytokine profiles, T-cell polarization, innate immune activation, and treatment responsiveness. Peripheral immune alterations may therefore be more prominent in selected immunologically active subgroups rather than being uniformly present across all patients with Meniere’s disease (10–12).

Studies of peripheral blood have shown altered cytokine profiles and shifts in immune-cell populations, whereas analyses of inner-ear tissue, endolymphatic sac fluid, and animal models have implicated inflammatory changes within the endolymphatic compartment and cochlea (6, 9, 13, 14). In parallel, familial aggregation, rare familial forms, and genome-wide association studies indicate a genetic contribution to disease susceptibility; these studies implicate rare variants in genes such as *OTOG*, *MYO7A* and *TECTA* and a broader polygenic architecture in sporadic MD (15–17). However, most genetic findings have not been systematically integrated with transcriptomic readouts or mapped to specific immune cell compartments. Thus, the genetically supported candidate genes and immune programs linking disease susceptibility to peripheral immune alterations remain incompletely characterized.

Zinc homeostasis is a plausible yet underexplored axis linking immunity, stress responses, and inner-ear vulnerability (18–20). Zinc functions both as a structural cofactor for many proteins and as a dynamic intracellular second messenger in immune cells and neurons. Members of the SLC39 (ZIP) family mediate zinc influx into the cytosol, whereas SLC30 (ZnT) transporters mediate zinc efflux or sequestration, thereby shaping the magnitude and timing of cellular zinc signals (21, 22). Perturbations of zinc transport alter epithelial-barrier integrity, innate and adaptive immune activation, and neuronal excitability in diverse contexts. In particular, the zinc importer SLC39A10 (ZIP10) modulates B-cell receptor signaling, humoral immunity, and hematopoietic stem-cell survival under stress. These observations raise the possibility that dysregulated zinc transport could modulate immune and neural circuits relevant to Meniere’s disease, but this hypothesis has not been systematically tested with patients or at single-cell resolution.

Technological advances in genomics and statistical genetics now make it possible to dissect complex axes in a unified framework. Bulk RNA sequencing of peripheral blood captures global immune and metabolic states but averages signals across diverse cell types. Conversely, single-cell RNA sequencing (scRNA-seq) overcomes this limitation by resolving transcriptional states at single cell resolution, enabling precise definition of immune-cell subsets, activation states, and developmental trajectories (23, 24). Expression quantitative trait loci (eQTL) analyses link genetic variants to variation in gene expression, and genome-wide association study (GWAS) summary statistics capture common-variant associations with disease risk.

Using methods such as two-sample Mendelian randomization and statistical colocalization, investigators can move beyond correlation and obtain evidence for potential causal relationships between gene expression and disease (25–27). Network-based approaches, including weighted gene co-expression network analysis (WGCNA), immune deconvolution, gene set variation analysis (GSVA), cell–cell communication inference, and pseudotime modelling, further connect genes, pathways, and intercellular interactions into coherent disease mechanisms.

In this study, we used this integrated toolbox to dissect the molecular and cellular architecture of Meniere’s disease. Using peripheral blood bulk transcriptomes and scRNA-seq datasets from patients with Meniere’s disease and healthy controls, we first identified MD-associated co-expression modules and differentially expressed genes, and anchored these signatures to specific immune cell types. We then integrated blood eQTL data with Meniere’s disease GWAS summary statistics in a two-sample Mendelian randomization framework, refined by colocalization, to nominate genes whose genetically regulated expression is associated with Meniere’s disease risk. Focusing on convergent candidates, we then used immune-cell signature-enrichment analysis, enrichment analyses, and regulatory-network reconstruction to delineate the MD-risk associated genes, their pathway contexts, transcription factor and microRNA regulators, and their embedding within an altered immune landscape. At single-cell resolution, we mapped these gene programs to defined CD4^+^ T-cell and NK-cell subsets, inferred rewired ligand–receptor communication networks, and reconstructed CD4^+^ T-cell differentiation trajectories. Lastly, we validated key candidates at both mRNA and protein levels in an independent clinical cohort.

By integrating these complementary layers of evidence, we prioritized *SLC39A10*, *GAB1*, and *XCL2* as candidate genes associated with Meniere’s disease and mapped their expression-related programs to defined peripheral immune-cell populations. On the basis of the known biological functions of these genes and their convergent genomic and transcriptomic associations, we propose a putative zinc transport–immune framework linking altered *SLC39A*10, *GAB1*, and *XCL2* expression to peripheral immune remodeling. Because zinc concentrations, transporter activity, and downstream immune functions were not directly measured, this framework is presented as a hypothesis for future mechanistic investigation rather than as an experimentally established pathogenic mechanism.

## 2 Materials and methods

### 2.1 Overall study design

**Figure 1:**
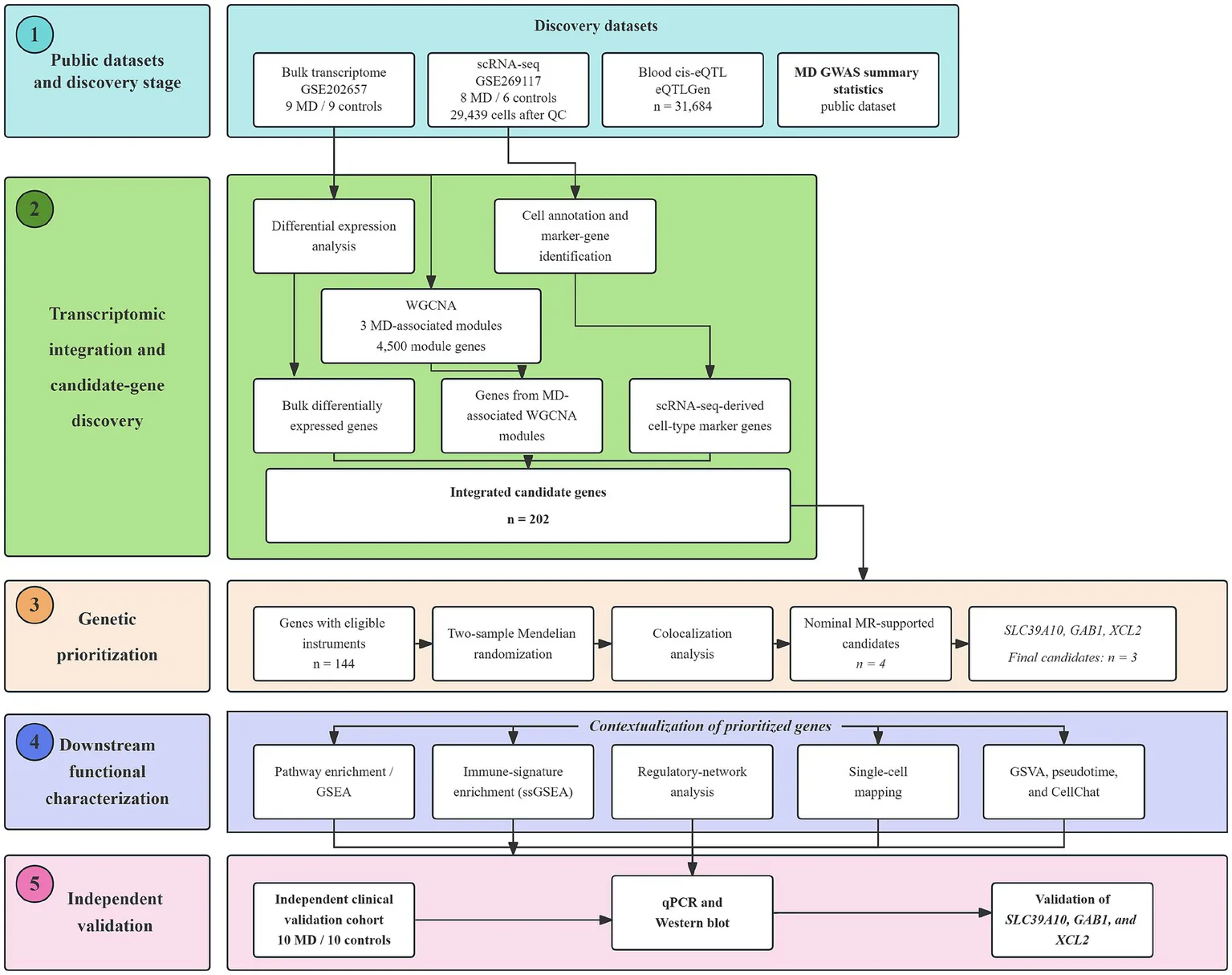
Overall study design and integrative candidate-gene prioritization workflow. Peripheral-blood bulk transcriptomic data from GSE202657, comprising 9 patients with Meniere’s disease and 9 healthy controls, were integrated with scRNA-seq data from GSE269117, comprising 8 patients and 6 controls, with 29,439 cells retained after quality control. Genes from three MD-associated WGCNA modules were integrated with bulk differentially expressed genes and scRNA-seq-derived cell-type marker genes, yielding 202 candidate genes. Blood cis-eQTL data from 31,684 individuals and Meniere’s disease GWAS summary statistics were subsequently used for two-sample Mendelian randomization and colocalization analyses. Of the 202 candidate genes, 144 had eligible genetic instruments, four showed nominal associations in the primary MR analysis, and *SLC39A10*, *GAB1*, and *XCL2* were prioritized for downstream functional characterization. Their expression changes were independently evaluated by qPCR and Western blot in a validation cohort comprising 10 patients with Meniere’s disease and 10 healthy controls.

The purpose of this study was to prioritize genetically supported candidate genes associated with Meniere’s disease and to characterize the peripheral immune-cell programs in which these genes are embedded. To achieve this goal, we integrated bulk transcriptomic data, single-cell RNA sequencing data, blood expression quantitative trait loci, and genome-wide association study summary statistics. We first identified MD-associated transcriptional signatures and co-expression modules in peripheral blood and mapped these signatures to specific immune-cell populations in the scRNA-seq dataset. We then applied two-sample Mendelian randomization to evaluate potential directional associations between genetically predicted gene expression and MD risk, followed by colocalization analysis to assess whether the corresponding eQTL and disease-association signals were compatible with shared causal variants. Finally, the prioritized candidate genes were validated at the mRNA and protein levels in an independent clinical cohort. The overall study design and integrative candidate-gene prioritization workflow are summarized in Figure 1.

### 2.2 Public datasets and data sources

#### 2.2.1 Bulk transcriptome data

Peripheral blood transcriptome data for Meniere’s disease were obtained from the Gene Expression Omnibus dataset GSE202657, which includes nine patients with Meniere’s disease and nine healthy controls profiled on the GPL23159 platform. Raw expression matrices and sample annotations were downloaded together with the corresponding platform file. Probe-level data were mapped to official gene symbols based on the platform annotation, and probes mapping to the same gene were collapsed by averaging. The resulting gene-level expression matrix and curated clinical information were used for downstream bulk analyses.

#### 2.2.2 Single-cell RNA-seq data

Single-cell RNA-seq data were obtained from GSE269117, which includes peripheral blood mononuclear cells from eight patients with Meniere’s disease and six healthy controls. Raw unique molecular identifier (UMI) count matrices were processed using the Seurat R package (version 4.3.0.1). Seurat objects were generated from the raw count matrices for each sample, followed by quality control to remove low-quality cells. The filtered samples were subsequently integrated for joint analysis. Integration was performed using Seurat’s anchor-based workflow (FindIntegrationAnchors and IntegrateData).

#### 2.2.3 Blood eQTL and MD GWAS summary statistics

Cis-eQTL summary statistics for whole blood were obtained from the eQTLGen Consortium, which aggregates data from 37 cohorts totaling 31,684 individuals.^1^

GWAS summary statistics for Meniere’s disease (MD) were obtained from the IEU OpenGWAS database (dataset: ebi-a-GCST90018880), derived from the cross-population GWAS study by Sakaue et al. These summary statistics were used as the outcome dataset for Mendelian randomization and colocalization analyses (28).

#### 2.2.4 Availability of clinical metadata

Available demographic and clinical annotations for the public transcriptomic and single-cell datasets were reviewed. However, detailed clinical variables, including disease stage, attack frequency, unilateral or bilateral involvement, recent attack status, treatment exposure, and inflammatory or autoimmune phenotypes, were not consistently reported across the datasets and therefore could not be incorporated into stratified or covariate-adjusted analyses.

### 2.3 Processing and annotation of single-cell RNA-seq data

#### 2.3.1 Quality control and normalization

Single-cell RNA-seq data from GSE269117, including eight Meniere’s disease (MD) samples and six healthy control samples, were analyzed using the Seurat R package (version 4.3.0.1). Genes detected in fewer than three cells were excluded. To remove low-quality cells, empty droplets, and potential doublets, cells were retained if they contained between 300 and 7,000 detected genes (nFeature_RNA). In addition, cells with a mitochondrial gene proportion greater than 40% were excluded, as elevated mitochondrial content is commonly indicative of cellular stress or apoptosis. After quality control, 29,439 high-quality cells and 19,684 genes were retained for downstream analyses.

Gene expression counts were normalized using the NormalizeData function (log-normalization), and the top 2,000 highly variable genes were identified with FindVariableFeatures. Principal component analysis was then conducted on the scaled expression of these variable genes. The number of principal components used for clustering and visualization was selected based on elbow plots and JackStraw analysis.

#### 2.3.2 Clustering and dimensionality reduction

Shared nearest-neighbor graphs were constructed using the FindNeighbors function on the selected principal components, and unsupervised clustering was performed with FindClusters at a resolution of 0.4. Uniform manifold approximation and projection (UMAP) embedding were generated via RunUMAP to visualize the global and local organization of cell populations in two-dimensional space.

#### 2.3.3 Cell-type annotation and identification of cluster marker genes

Cell-type annotation was performed using the SingleR algorithm (version 2.0.0) with the HumanPrimaryCellAtlasData reference from the celldex package. Automated labels were reviewed and refined using canonical marker genes to ensure biologically coherent annotations. Cluster-enriched marker genes were identified using the FindAllMarkers function in Seurat with the Wilcoxon rank-sum test, comparing each annotated cluster with the remaining cells. These marker genes were used to characterize cell-type-specific transcriptional features and were not interpreted as MD-versus-control differentially expressed genes. Marker-expression patterns across cell types were visualized using UMAP plots, bubble plots, and violin plots with scRNAtoolVis.

### 2.4 Bulk differential expression and weighted gene co-expression network analysis

#### 2.4.1 Differentially expressed genes in bulk peripheral blood

The processed GSE202657 expression matrix was analyzed using the limma package. Expression distributions across the 18 samples were first examined, followed by between-array quantile normalization using the normalizeBetweenArrays function. Sample-level quality control was subsequently performed using relative log expression (RLE) analysis, principal-component analysis, and hierarchical sample clustering. For each gene, RLE values were calculated by subtracting the median expression across all samples from the expression value in each individual sample. Principal-component analysis was performed on the normalized gene-expression matrix, and hierarchical clustering was conducted using Pearson-correlation distance and average linkage to identify potential sample-level outliers. GSE202657 was generated within a single GEO series on the GPL23159 platform, and the available sample annotations did not identify a separate experimental batch variable.

Linear models were then fitted to compare patients with Meniere’s disease and healthy controls, and empirical Bayes moderation was applied to obtain stabilized variance estimates. Differentially expressed genes were defined by an absolute log2 fold change greater than 1 and a Benjamini–Hochberg-adjusted *p* value below 0.05.

Volcano plots of DEGs were generated with ggplot2 (version 3.5.1), and hierarchical clustering heatmaps of selected DEGs were drawn using pheatmap (version 1.0.12).^2^ DEGs identified from bulk RNA-seq were subsequently intersected with scRNA-seq markers and weighted gene co-expression network analysis module genes to delineate robust MD-associated gene sets.

#### 2.4.2 Weighted gene co-expression network analysis

Weighted gene co-expression network analysis was performed using the WGCNA R package on the GSE202657 expression matrix. Samples were first subjected to hierarchical clustering to assess potential outliers. Pairwise correlations between gene-expression profiles were then transformed into weighted connection strengths using a soft-thresholding power, *β*. This transformation reduces the contribution of weak correlations while preserving stronger co-expression relationships, thereby facilitating the identification of groups of genes with coordinated expression patterns.

**Figure 2:**
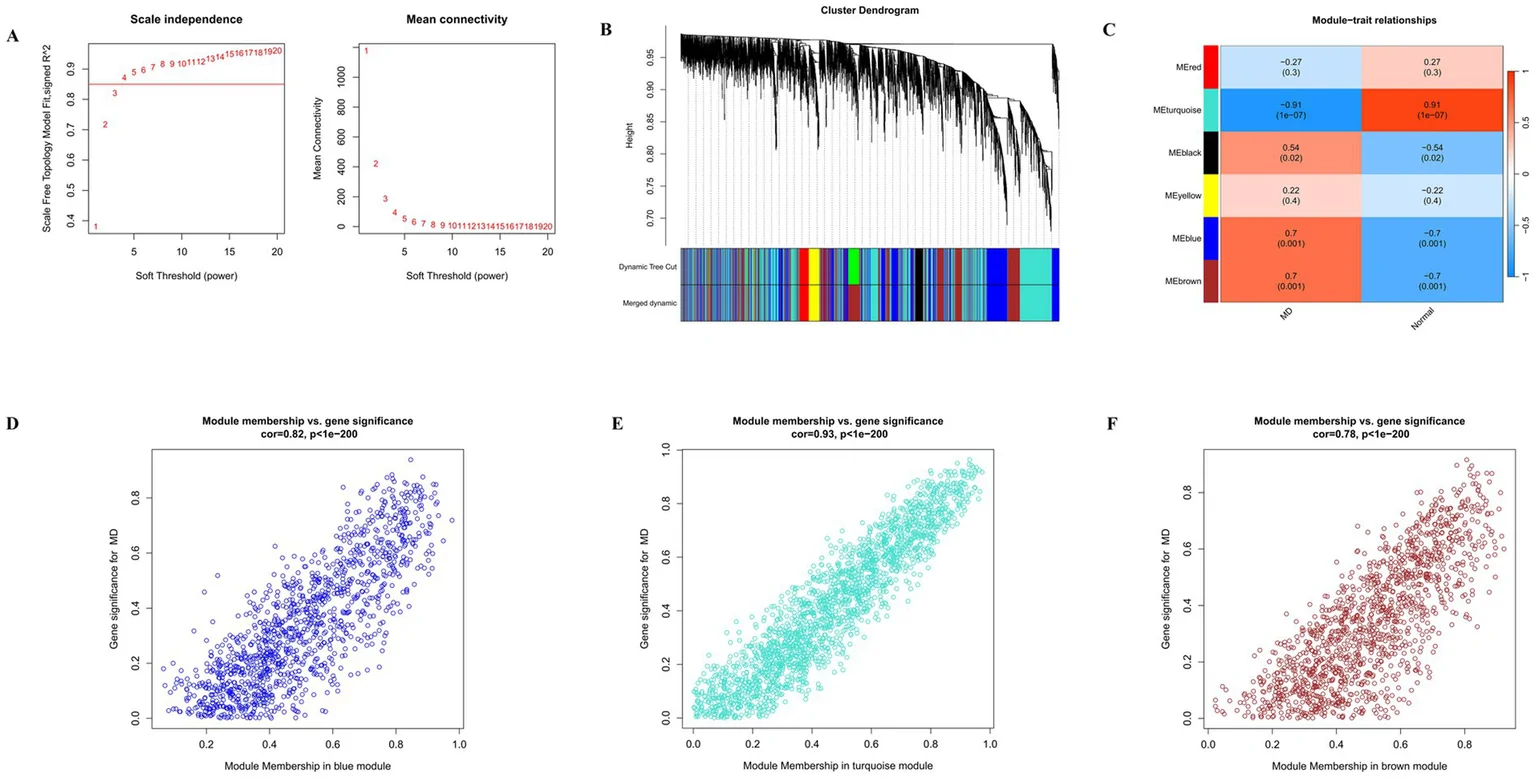
Weighted gene co-expression network construction and identification of Meniere’s disease-associated modules. **(A)** Selection of the soft-thresholding power based on the scale-free topology fit index and mean network connectivity. A power of *β* = 4 was selected to balance an approximately scale-free network structure with preservation of adequate network connectivity. **(B)** Gene-clustering dendrogram and dynamic tree cutting showing the co-expression modules identified from GSE202657. **(C)** Heat map of correlations between module eigengenes and Meniere’s disease status. Each cell shows the correlation coefficient and corresponding *p-*value. **(D–F)** Relationships between module membership and gene significance in the blue, turquoise, and brown modules, respectively. These modules showed the strongest associations with MD status and were retained for subsequent integrative analyses.

Candidate soft-thresholding powers were evaluated according to both the scale-free topology fit index and mean network connectivity. A soft-thresholding power of *β* = 4 was selected as the lowest candidate power at which the scale-free topology fit index reached approximately 0.85, while mean connectivity remained adequate and began to stabilize (Figure 2A). This value was therefore considered to provide an appropriate balance between an approximately scale-free network structure and preservation of overall network connectivity.

The resulting adjacency matrix was transformed into a topological overlap matrix (TOM), which incorporates both direct gene–gene connections and shared network neighbors. Genes were hierarchically clustered according to TOM-based dissimilarity, and initial co-expression modules were identified using dynamic tree cutting. Modules with similar eigengene profiles were subsequently merged using a module eigengene dissimilarity threshold of 0.5.

Module eigengenes, defined as the first principal component of each module’s expression profile, were correlated with Meniere’s disease status to identify disease-associated modules. Genes within the modules showing the strongest associations with disease status were retained as the WGCNA-derived gene set for subsequent integration with the bulk differential-expression and single-cell analyses.

### 2.5 Functional enrichment and pathway analysis

#### 2.5.1 Gene ontology **and** Kyoto encyclopedia of genes and genomes **enrichment analysis**

To characterize the biological functions of MD-related gene sets, GO and KEGG pathway enrichment analyses were performed using clusterProfiler (29). Unless otherwise stated, adjusted *p* values < 0.05 were considered statistically significant. Enrichment results were visualized using GOplot (30), including chord and circular plots that depict gene–term relations and the overall functional landscape.

#### 2.5.2 Gene set enrichment analysis of prioritized candidate genes

To further interrogate the pathways linked to MR-supported candidate genes, we conducted gene set enrichment analysis using the GSEA function in clusterProfiler. The curated KEGG-based gene set collection “c2.kegg.v7.4.symbols” from the Molecular Signatures Database was used as the reference. Genes were ranked according to their differential expression statistics, and enrichment scores were computed by permutation testing. Nominal *p* values < 0.05 were considered indicative of significant enrichment. Unlike over-representation analyses, GSEA accounts for the coordinated up- and downregulation of genes across the entire ranked list, providing high sensitivity to pathway-level shifts.

### 2.6 Mendelian randomization analysis

#### 2.6.1 Selection of genetic instruments

To evaluate potential directional associations of gene expression on Meniere’s disease risk, we performed two-sample Mendelian randomization using blood cis-eQTLs as instruments. For each candidate gene, SNPs strongly associated with gene expression in eQTLGen were considered initial instrumental variables (IVs), subject to the following filters:

1. Association strength: eQTL *p* value < 1 × 10^^−5^ for the gene–SNP association.
2. Instrument strength: F statistic computed as F = β_exposure^2^/SE_exposure^2^ >10 to minimize weak-instrument bias.
3. Minor allele frequency: MAF ≥0.01.
4. Linkage disequilibrium pruning: SNPs were clumped using clump_data from ieugwasr with a 1,000 kb window and r^2^ = 0.01, retaining independent variants.
5. Palindrome variants: Ambiguous palindromic SNPs with intermediate allele frequencies were excluded.
6. Harmonization: Exposure (eQTL) and outcome (MD GWAS) datasets were harmonized using harmonise_data to align effect alleles and directions.
7. Outcome-driven instruments: SNPs with strong direct associations with MD (*p* < 5 × 10^^−8^) were removed to reduce reverse causation and overt horizontal pleiotropy.

#### 2.6.2 MR models and effect estimation

Mendelian randomization analyses were performed using the TwoSampleMR package (version 0.5.10),^3^ with genetically predicted gene expression as the exposure and Meniere’s disease as the outcome. Five complementary MR estimators were used:

1. Inverse variance weighted (IVW), the primary analysis, which combines Wald ratios of individual SNPs using inverse-variance weighting.
2. MR-Egger regression, which fits a weighted linear regression enabling a non-zero intercept to account for directional pleiotropy under the InSIDE assumption.
3. Weighted median estimator, which yields a consistent estimate if at least 50% of the total weight stems from valid instruments.
4. Simple mode estimator, which assumes that the largest cluster of instruments corresponds to the true causal effect.
5. Weighted mode estimator, which extends the simple mode by incorporating instrument weights.

For each gene, we obtained MR effect estimates and *p* values from all five methods, taking the IVW estimate as the principal measure and using the others for sensitivity assessments. A two-sided *p* value < 0.05 for IVW was considered evidence of a nominally significant MR association.

#### 2.6.3 Heterogeneity and horizontal pleiotropy

Heterogeneity across SNP-specific estimates was evaluated with Cochran’s Q statistics implemented in mr_heterogeneity within TwoSampleMR. Non-significant Q statistics (*p* > 0.05) were interpreted as an absence of substantial heterogeneity among instruments.

Horizontal pleiotropy was assessed in two ways. First, the MR-Egger intercept test was used to detect directional pleiotropy; an intercept significantly different from zero (*p* < 0.05) suggests potential pleiotropy. Second, the MR-PRESSO method (Mendelian Randomization Pleiotropy RESidual Sum and Outlier) was used to identify and correct for outlier SNPs driving pleiotropic distortion, using 1,000 permutations for the global test. A non-significant MR-PRESSO global test (*p* > 0.05) was taken as evidence against substantial horizontal pleiotropy.

#### 2.6.4 Directionality assessment

To verify that the inferred direction of causality was from gene expression to Meniere’s disease, instead of the reverse, we applied Steiger filtering. For each instrument, we compared the variance explained (R(2)) in the exposure (gene expression) versus the outcome (MD). Instruments for which the exposure *R*^2^ exceeded the outcome *R*^2^ were considered consistent with the hypothesized direction (expression → MD). Genes whose instrument sets failed this directionality check were not considered as prioritized MR-supported candidates.

### 2.7 Colocalization analysis

For genes supported by Mendelian randomization, we conducted colocalization analyses to assess whether the same causal variant underlies both the eQTL and MD GWAS signals. Colocalization was performed using the coloc package^4^.

Within predefined genomic windows around each gene, coloc jointly modeled eQTL and GWAS summary statistics to estimate the posterior probabilities of five mutually exclusive hypotheses:

1. PP_H0: no association with either trait.
2. PP_H1: association with gene expression only.
3. PP_H2: association with MD only.
4. PP_H3: association with both traits driven by distinct causal variants.
5. PP_H4: association with both traits driven by a shared causal variant.

We interpreted PP_H4 > 0.8 as strong evidence that the eQTL and MD GWAS signals are consistent with a shared causal variant at the locus.

### 2.8 Immune-cell signature enrichment and gene–immune correlations

Immune-cell signature enrichment in the GSE202657 bulk peripheral-blood expression dataset was quantified using single-sample gene set enrichment analysis implemented in the GSVA R package with method = “ssgsea.” A predefined immune-cell marker-gene table was grouped according to the Cell.type annotation, and unique genes listed in the Metagene column were used to construct each immune-cell signature. A total of 28 signatures were evaluated, representing activated B cells, activated CD4^+^ T cells, activated CD8^+^ T cells, activated dendritic cells, CD56^bright natural killer cells, CD56^dim natural killer cells, central-memory CD4^+^ T cells, central-memory CD8^+^ T cells, effector-memory CD4^+^ T cells, effector-memory CD8^+^ T cells, eosinophils, γδ T cells, immature B cells, immature dendritic cells, macrophages, mast cells, myeloid-derived suppressor cells, memory B cells, monocytes, natural killer cells, natural killer T cells, neutrophils, plasmacytoid dendritic cells, regulatory T cells, T follicular helper cells, type 1 helper T cells, type 17 helper T cells, and type 2 helper T cells. The resulting ssGSEA scores represent relative enrichment of the corresponding immune-cell transcriptional signatures within each sample and should not be interpreted as direct measurements of absolute cell proportions.

### 2.9 Regulatory network construction and single-cell functional analyses

#### 2.9.1 Transcription factor and miRNA regulatory networks

To identify upstream regulatory mechanisms of Mendelian randomization-supported candidate genes, we integrated transcription factor (TF)–target and microRNA (miRNA)–target interactions. Transcription factor–gene regulatory relations were obtained from ENCODE (31), and miRNA–gene interactions were retrieved from MiRTarBase (32). Combined TF–gene and miRNA–gene networks were constructed and visualized using the NetworkAnalyst platform (33), enabling the identification of key regulatory hubs potentially controlling MD-associated gene programs.

#### 2.9.2 Gene set variation analysis in single cells

To evaluate the activity of the prioritized candidate genes program (*SLC39A10*, *GAB1*, and *XCL2*) at single-cell resolution, we performed gene set variation analysis (GSVA) 32 using the GSVA R package (version 1.46) (34). Gene-set activity scores were calculated for individual cells and summarized according to cell type and disease status. Differences in GSVA scores between Meniere’s disease (MD) and control samples were assessed using two-sided Wilcoxon rank-sum tests. By integrating GSVA scores with cell-type annotations, we examined the distribution of this gene program across immune-cell populations, with particular focus on CD4^+^ T cells and other major immune subsets, to characterize immune-cell–specific alterations in Meniere’s disease.

#### 2.9.3 Reclustering and transcriptional states of CD4^+^ T-cell subsets

CD4^+^ T cells were placed in subsets from the integrated scRNA-seq object and reanalyzed with Seurat. After normalization and scaling, we performed principal component analysis, clustering, and UMAP visualization to resolve CD4^+^ T-cell subpopulations. Marker genes for each CD4^+^ T-cell subset were identified and functionally annotated. Expression patterns of prioritized candidate genes across CD4^+^ T-cell subsets were then examined to infer their potential functions in shaping T-cell heterogeneity in Meniere’s disease.

#### 2.9.4 Cell–cell communication analysis

Cell–cell communication was inferred using CellChat (version 1.6.1). Normalized single-cell expression matrices and cell-type annotations were used as inputs (35). Based on the built-in CellChatDB, CellChat identified overexpressed ligands and receptors and inferred potential ligand–receptor interactions and signaling pathways among annotated immune-cell populations. Global and pathway-specific communication networks were visualized using bubble plots, river plots, and circle plots.

CellChat and GSVA were conducted as separate analytical procedures with distinct purposes. CellChat was used to infer potential ligand–receptor-mediated communication among immune-cell populations, whereas GSVA was used to summarize the relative activity of the *SLC39A10/GAB1/XCL2* gene set across cells and cell types. The GSVA scores were not incorporated into the CellChat model, and no direct correlation or other formal statistical association between GSVA scores and CellChat-derived interaction numbers or communication strengths was performed. Accordingly, the GSVA and CellChat results were interpreted as complementary but analytically independent observations.

#### 2.9.5 Pseudotime trajectory analysis

To reconstruct developmental or activation trajectories of CD4^+^ T cells, we performed pseudotime analysis with monocle (36) (version 2.28.0^5^). Highly variable genes within the CD4^+^ T-cell subset were used to define the low-dimensional manifold and infer trajectories, including potential branch points representing divergent cell fates or states.

Expression dynamics of prioritized candidate and immune-related genes were mapped along pseudotime to delineate their temporal regulation during CD4^+^ T-cell state transitions. Because of limited cell numbers in some minor subclusters, we did not overinterpret single-cell metabolic pathway enrichments that did not reach stable statistical support.

### 2.10 Clinical cohort and experimental validation

#### 2.10.1 Study participants and sample collection

For experimental validation, we recruited an independent cohort comprising 10 patients with Meniere’s disease and 10 healthy controls. Peripheral venous blood was collected from all participants, and buffy-coat fractions were isolated for downstream molecular assays. For the patients with Meniere’s disease, available clinical records were reviewed to extract disease duration, laterality, recorded clinical stage, attack frequency, treatment history, month and year of blood sampling, and the presence of documented inflammatory or autoimmune phenotypes. Individual-level clinical characteristics are summarized in Supplementary Table S5.

The exact interval between the most recent vertigo attack and blood sampling and the temporal relationship between treatment exposure and sample collection were not consistently documented. Therefore, the samples could not be uniformly classified as having been collected during an active attack or an interictal period. The validation cohort was not individually matched by age or sex, and detailed individual-level demographic data were not available for export under institutional data-governance and patient-privacy requirements.

The study protocol was approved by the Ethics Committee of Drug Clinical Trials, Shenzhen Second People’s Hospital (approval no. 2025–523-01PJ), and written informed consent was obtained from all participants.

#### 2.10.2 Reagents and instruments

Major instruments included a refrigerated centrifuge (Eppendorf 5810R), real-time PCR system (LongGene Q2000B), and chemiluminescence imaging system (Tanon 4,600; Shanghai, China). Key reagents were RNA extraction kits (Sevier G3013), reverse transcription and qPCR kits (Allsheng AU341-02 and AQ601-02), RIPA lysis buffer and PMSF (Beyotime P0013B and ST506), BCA protein assay kit (Thermo Fisher Scientific), and primary antibodies against *SLC39A10, LRRFIP1, GAB1, XCL2* and *β-actin*, together with appropriate HRP-conjugated secondary antibodies.

#### 2.10.3 RNA extraction and reverse transcription

Total RNA was extracted from buffy coats using the manufacturer’s protocol. Briefly, 1 mL of RNA extraction reagent was added to each sample and incubated at ambient temperature for 5 min, followed by addition of 200 μL chloroform, vigorous shaking, and a further 5-min incubation. Samples were centrifuged at 12,000 g for 15 min at 4 °C, and the aqueous phase was transferred to a new tube. An equal volume of isopropanol was added, mixed, and incubated at ambient temperature for 15 min, followed by centrifugation at 12,000 g for 10 min at 4 °C.

The RNA pellet was washed with 1 mL of 75% ethanol, centrifuged at 7,500 g for 5 min at 4 °C, air-dried for approximately 10 min, and dissolved in 20 μL of DEPC-treated water. RNA purity and concentration were measured with a UV spectrophotometer using DEPC water as blank.

Complementary DNA (cDNA) was synthesized using a reverse transcription kit according to the manufacturer’s instructions. A 20 μL reaction mixture was incubated at 50 °C for 5 min and then at 85 °C for 2 min to inactivate the reverse transcriptase. cDNA products were stored on ice and used immediately for quantitative PCR or stored at −80 °C.

#### 2.10.4 Quantitative real-time PCR

Quantitative real-time PCR (qPCR) was performed using PerfectStart® Green qPCR SuperMix (Allsheng, Hangzhou, China) in 20 μL reaction mixtures containing 5 μL qPCR SuperMix, 0.2 μL forward primer (10 μM), 0.2 μL reverse primer (10 μM), 2 μL cDNA template, and nuclease-free water to volume. Amplification was carried out under the following cycling conditions: initial denaturation at 95 °C for 30 s, followed by 46 cycles of 95 °C for 5 s and 60 °C for 30 s. A melting-curve analysis (95 °C for 15 s, 60 °C for 60 s, and 95 °C for 15 s) was performed at the end of each run to verify amplification specificity.

Target genes included *SLC39A10*, *LRRFIP1*, *GAB1*, and *XCL2*, with *β*-actin used as the internal reference gene. Primers were synthesized by Sangon Biotech (Shanghai, China), and their sequences are listed in Table 1. Relative mRNA expression levels were calculated using the 2^−ΔΔCt method after normalization to β-actin and were compared between the Meniere’s disease (MD) and control groups.

**Table 1:** qPCR primer sequences.

Target gene	Forward primer (5′→3′)	Reverse primer (5′→3′)
*β-actin*	GGCCGAGGACTTTGATTGCA	GGGACTTCCTGTAACAACGCA
*SLC39A10*	AGTGCTGGATTGACAGGAGG	CACCAAGGCTACATAGAGGA
*LRRFIP1*	GACTTCCGACACCCTCAATA	CATCCCTTTGTAGGTCCATT
*GAB1*	ATTCGTCTGTCTTGGCTTTG	AACTGTGGGCACTCCTTTCT
*XCL2*	TGCCAGTTAGCAGAATCAAG	CTACTAGCCAGTCAGGGTCA

#### 2.10.5 Protein extraction and quantification

For protein extraction, buffy coat samples were pelleted by centrifugation and resuspended in prechilled RIPA lysis buffer supplemented with PMSF immediately before use. Samples were incubated on ice for 30 min with intermittent vortexing every 10 min, followed by centrifugation at 12,000 g for 15 min at 4 °C. The resulting supernatant was collected as total protein. Protein concentrations were determined using the BCA assay according to the manufacturer’s instructions.

#### 2.10.6 Western blot

Proteins were separated on 6–15% SDS–polyacrylamide gels (gel compositions are provided in Supplementary Tables S3, S4) under standard electrophoresis conditions (approximately 60 V for 30 min for stacking gels and 110 V for 120 min for resolving gels). Equal amounts of protein were mixed with loading buffer, heated at 100 °C for 5–6 min, and cooled on ice before loading onto the gels.

Separated proteins were transferred onto polyvinylidene difluoride (PVDF) membranes (Millipore, IPVH00010) at 100 V for 50 min on ice. Membranes were blocked in 5% non-fat milk in TBST for 1–2 h at 37 °C and then washed three times with TBST. The membranes were incubated overnight at 4 °C with primary antibodies against *SLC39A10* (1:1,000), *LRRFIP1* (1:3,000), *GAB1* (1:1,000), *XCL2* (1:1,000), and *β*-actin (1:50,000).

The following day, membranes were washed three times with TBST and incubated with HRP-conjugated secondary antibodies (1:5,000 for anti-rabbit IgG and 1:10,000 for anti-mouse IgG) for 1 h at 37 °C, followed by three additional TBST washes. Protein bands were visualized using an enhanced chemiluminescence (ECL) substrate and captured with a chemiluminescence imaging system. Band intensities were quantified using ImageJ software, and expression levels of target proteins were normalized to β-actin. Normalized protein expression levels were compared between Meniere’s disease and control samples to validate transcriptomic findings at the protein level.

## 3 Results

### 3.1 Single-cell profiling delineates the peripheral immune-cell landscape in Meniere’s disease

**Figure 3:**
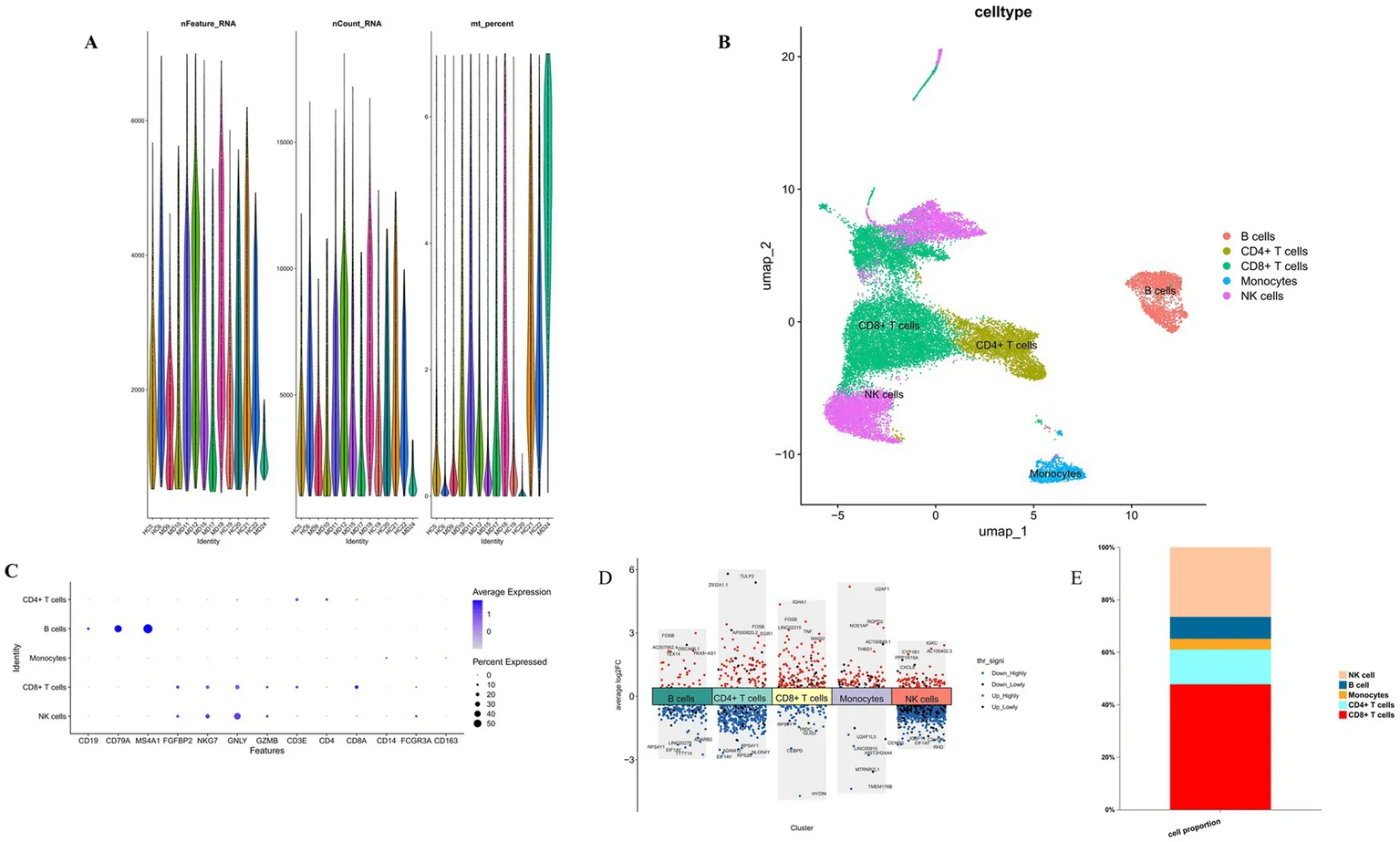
Single-cell landscape of peripheral immune cells in Meniere’s disease. **(A)** Quality-control metrics of peripheral blood scRNA-seq. Violin plots show the distributions of detected genes per cell (*nFeature_RNA*), unique molecular identifier (UMI) counts per cell (*nCount_RNA*) and percent of mitochondrial transcripts (*mt_percent*) across clusters after filtering. **(B)** Uniform manifold approximation and projection (UMAP) embedding of all high-quality cells colored by major immune lineages, including B cells, CD4^+^ T cells, CD8^+^ T cells, monocytes, and NK cells. **(C)** Dot plot of canonical marker genes used to annotate each immune-cell cluster. Dot size reflects the proportion of cells expressing a given gene in each cluster, and color indicates average scaled expression. **(D)** Marker-gene profiles across the annotated immune-cell lineages. Representative genes distinguishing each immune-cell lineage are shown, with red and blue indicating relatively higher and lower scaled expression, respectively. **(E)** Stacked bar plot showing the aggregate cellular composition of the five major immune-cell lineages across the integrated scRNA-seq dataset. This panel is descriptive and does not represent a sample-level comparison between MD and control groups.

We first used scRNA-seq (GSE269117) to map the peripheral immune landscape of patients with Meniere’s disease (MD). After stringent quality control, 29,439 high-quality cells were retained and projected into a two-dimensional UMAP space, where unsupervised clustering resolved five major lineages: B cells, CD4^+^ T cells, CD8^+^ T cells, monocytes, and NK cells (Figures 3A,B). Canonical markers confirmed the identity of each cluster and highlighted clear transcriptional separation between lineages (Figure 3C). Representative marker-gene profiles across the annotated immune-cell lineages are shown in Figure 3D.

The integrated scRNA-seq dataset contained five major immune-cell lineages, including B cells, CD4^+^ T cells, CD8^+^ T cells, monocytes, and NK cells. Figure 3E summarizes the aggregate cellular composition of the integrated dataset. Because this panel does not represent sample-level cell-type proportions, no formal statistical inference regarding differential cell abundance between MD and control groups was drawn from this visualization.

### 3.2 Co-expression network analysis identifies Meniere’s disease-associated gene modules

To complement the single-cell data with bulk-level information, we constructed a weighted gene co-expression network using the GSE202657 peripheral-blood transcriptomic dataset. Hierarchical clustering, dynamic tree cutting, and merging of modules with similar eigengene profiles identified six co-expression modules (Figures 2A,B). Correlation analysis between module eigengenes and disease status identified three MD-associated modules (Figures 2C–F). The turquoise module showed the strongest positive association with MD status (*r* = 0.91, *p* = 1 × 10^−7^), whereas the blue and brown modules were negatively associated with MD status (both *r* = −0.70, *p* = 0.001). Together, these three modules contained 4,500 genes, which were retained as the WGCNA-derived gene set for subsequent integrative prioritization.

### 3.3 Integrative prioritization identifies 202 Meniere’s disease-associated candidate genes

Before differential-expression analysis, we evaluated the sample-level quality of the GSE202657 expression matrix. The supplied expression values showed broadly comparable distributions across the 18 samples, which became closely aligned after quantile normalization (Supplementary Figures S3A,B). Following normalization, the RLE distributions were centered near zero across all samples, with no sample showing a marked global shift in expression distribution (Supplementary Figure S3C). Principal-component analysis and hierarchical sample clustering did not identify an isolated sample-level technical outlier; the dominant structure of the dataset was associated with MD versus control status (Supplementary Figures S3D,E). Because no explicit experimental batch variable was available in the accompanying metadata, these analyses do not definitively exclude all unrecorded technical effects but indicate that no obvious sample-level technical artifact was detected.

Bulk differential-expression analysis subsequently identified 3,489 genes that differed between MD and control samples at an adjusted *p-*value below 0.05 and an absolute log2 fold change greater than 1, including 1,799 upregulated and 1,690 downregulated transcripts (Supplementary Figures S1A,B). Given the relatively large number of differentially expressed genes, this set was treated as a discovery-stage bulk transcriptional signature and was subjected to further integration with WGCNA and single-cell evidence rather than being interpreted as a collection of independently validated disease-specific genes.

To prioritize MD-associated candidate genes using complementary transcriptomic evidence, we integrated three gene sets: (i) genes from the three MD-associated WGCNA modules, (ii) differentially expressed genes identified in bulk peripheral blood, and (iii) scRNA-seq-derived cell-type marker genes identified across the annotated immune-cell clusters. The intersection of these three gene sets yielded 202 candidate genes for subsequent genetic prioritization (Supplementary Figure S1C). The single-cell component was used to provide cell-type localization information and did not represent a formal MD-versus-control differential-expression analysis within each immune-cell lineage.

### 3.4 Functional enrichment implicates biosynthetic, regulatory and immune pathways

**Figure 4:**
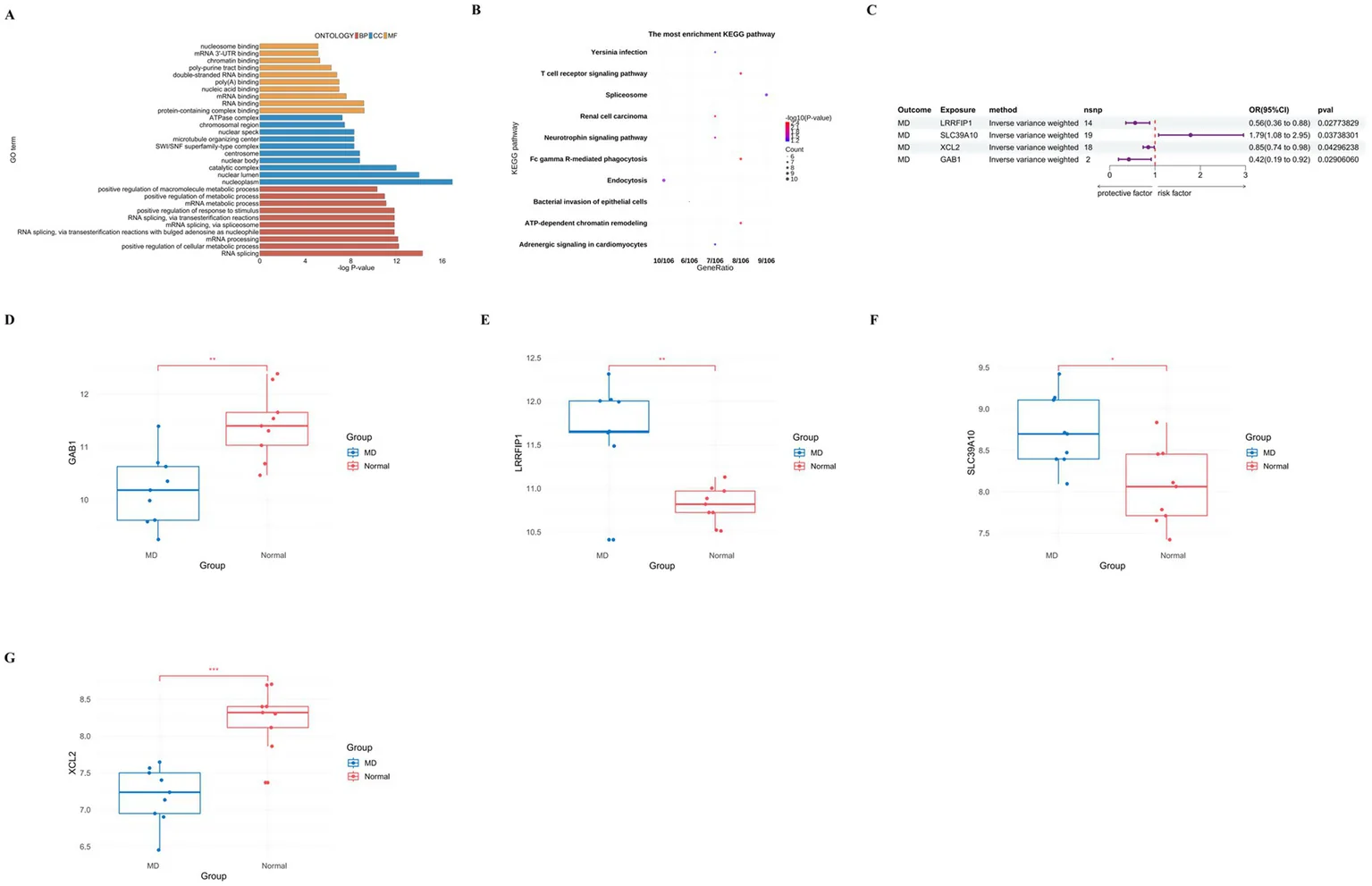
Pathway enrichment and genetic evidence prioritize candidate genes within a putative zinc transport–immune framework. **(A)** Gene Ontology enrichment analysis of the 202 candidate genes. The top enriched biological process, cellular component, and molecular function terms are shown; bar length represents –log_10_(*p* value). **(B)** KEGG pathway enrichment of the candidate genes. Dot size reflects the number of genes in each pathway, and color encodes –log_10_(*p* value). Immune-related pathways, including T-cell receptor signaling and FcγR-mediated phagocytosis, are prominently enriched. **(C)** Two-sample Mendelian randomization (MR) analysis linking genetically predicted gene expression to MD risk. Forest plot shows odds ratios (*ORs*) and 95% confidence intervals (CI) derived primarily from inverse variance-weighted estimates for *SLC39A10, LRRFIP1, GAB1*, and *XCL2*. Genetically predicted *SLC39A10* expression showed a positive association with MD risk, whereas genetically predicted *GAB1* and *XCL2* expression showed inverse associations. **(D–G)** Box plots of GAB1 **(D)**, LRRFIP1 **(E)**, SLC39A10 **(F)**, and XCL2 **(G)** mRNA expression in the discovery bulk-RNA cohort, comparing peripheral blood from patients with MD and healthy controls. Each dot represents one individual. *p*-values are from two-sided statistical tests; *p < 0.05, **p < 0.01, ***p < 0.001, ****p < 0.0001, “ns” not significant.

Gene Ontology enrichment analysis of the 202-gene set revealed 344 significantly enriched terms (adjusted *p* < 0.05), spanning 281 biological process, 36 cellular components, and 27 molecular function categories. Prominent biological processes included “positive regulation of macromolecule metabolic process,” “RNA splicing,” and “positive regulation of cellular metabolic process” (Figure 4A). These findings suggested that MD blood is characterized by coordinated rewiring of biosynthetic and regulatory programs.

KEGG analysis identified nine significantly enriched pathways, such as “Fc gamma R-mediated phagocytosis,” “endocytosis,” “neurotrophin signaling,” and “ATP-dependent chromatin remodeling” (Figure 4B). Although several of these pathways are classically described in cancer or infectious contexts, they converge on immune activation, phagocytic clearance, and stress-response signaling. These results provided functional context for the MD gene set and hinted at an interface between immune regulation and cell-intrinsic stress responses. Thus, we were motivated to search for genetically supported candidate regulators within these pathways.

### 3.5 Genetic analyses prioritize *SLC39A10*, *GAB1*, and *XCL2* as Meniere’s disease-associated candidate genes

To evaluate potential directional associations between genetically predicted gene expression and Meniere’s disease risk, we performed two-sample Mendelian randomization using blood cis-eQTLs for the 202 candidate genes as exposures and MD GWAS summary statistics as the outcome. After stringent instrument selection, data harmonization, linkage disequilibrium (LD) pruning, and exclusion of palindromic and outcome-associated variants, 144 genes retained valid SNP instruments for MR analysis. Among these 144 genes, four showed nominally significant associations in the primary inverse-variance weighted (IVW) analysis (*p* < 0.05): *SLC39A10*, for which increased expression was associated with higher MD risk; *LRRFIP1*, for which higher expression was associated with lower risk; *GAB1*, for which higher expression was associated with lower risk; and *XCL2*, for which higher expression was associated with lower risk (Figure 4C).

Sensitivity analyses were performed to further evaluate the stability of these associations. Cochran’s Q statistics did not indicate substantial heterogeneity across SNP-specific estimates for any of the four genes (Table 2). *LRRFIP1* showed evidence of directional pleiotropy in the MR-Egger intercept test, which reduced confidence in its prioritization. By contrast, *SLC39A10* and *XCL2* showed no clear evidence of substantial horizontal pleiotropy, whereas interpretation of *GAB1* remained more cautious because only two SNP instruments were available. Steiger directionality tests further showed that, for all four genes, the variance explained in gene expression exceeded that explained in MD (correct directionality consistent with expression → MD = TRUE; Table 3), supporting a direction of effect from gene expression to disease rather than the reverse. Together with the concordant directions observed between MR estimates and bulk expression changes, these findings supported the selection of *SLC39A10*, *GAB1*, and *XCL2* as prioritized genes for downstream analyses.

**Table 2:** Results of Mendelian randomization tests.

**Outcome**	**Exposure**	**Method**	**nsnp**	**Q**	**Q_df**	**Q_pval**	**intercept**	**intercept_se**	**intercept_pval**
MD	*LRRFIP1*	Inverse variance weighted	14	15.30162245	13	0.288903419	NA	NA	NA
MD	*LRRFIP1*	MR Egger	14	9.12680439	12	0.692063186	0.082624524	0.033250401	0.028698233
MD	*LRRFIP1*	MRPRESSO_raw	14	15.30162245	13	0.288903419	NA	NA	0.287
MD	*SLC39A10*	Inverse variance weighted	19	19.17234877	18	0.381283245	NA	NA	NA
MD	*SLC39A10*	MR Egger	19	14.96933802	17	0.597690286	−0.096519362	0.047079755	0.056101512
MD	*SLC39A10*	MRPRESSO_raw	19	19.17234877	18	0.381283245	NA	NA	0.371
MD	*XCL2*	Inverse variance weighted	18	14.63802497	17	0.621536313	NA	NA	NA
MD	*XCL2*	MR Egger	18	14.60971835	16	0.553387165	0.00241019	0.014325417	0.868498363
MD	*XCL2*	MRPRESSO_raw	18	14.63802497	17	0.621536313	NA	NA	0.746
MD	*GAB1*	Inverse variance weighted	2	0.669769517	1	0.413131976	NA	NA	NA

**Table 3:** Results of Steiger filtering direction test; correct_causal_direction indicates whether the test is passed; steiger_pval represents the *p*-value in the test.

**Exposure**	**Outcome**	**snp_r2.exposure**	**snp_r2.outcome**	**correct_causal_direction**	**steiger_pval**
*GAB1*	MD	0.001112759	4.58E-06	TRUE	6.70E-08
*LRRFIP1*	MD	0.043435741	8.81E-06	TRUE	1.21E-284
*SLC39A10*	MD	0.027710056	1.87E-05	TRUE	3.24E-176
*XCL2*	MD	0.056597568	2.59E-05	TRUE	0

By integrating MR estimates with bulk expression data, we observed concordant directions of effect for three genes. *SLC39A10* was upregulated in MD and genetically associated with increased disease risk, whereas *GAB1* and *XCL2* were downregulated and showed genetically protective effects. In contrast, *LRRFIP1* displayed discordant directions between MR estimates and bulk transcriptomic results and was therefore not prioritized for further mechanistic analyses. Consequently, we focused on *SLC39A10*, *GAB1*, and *XCL2* as key MD-related genes for downstream analyses (Figures 4D–G).

### 3.6 Immune-cell signature enrichment patterns associated with prioritized candidate genes

**Figure 5:**
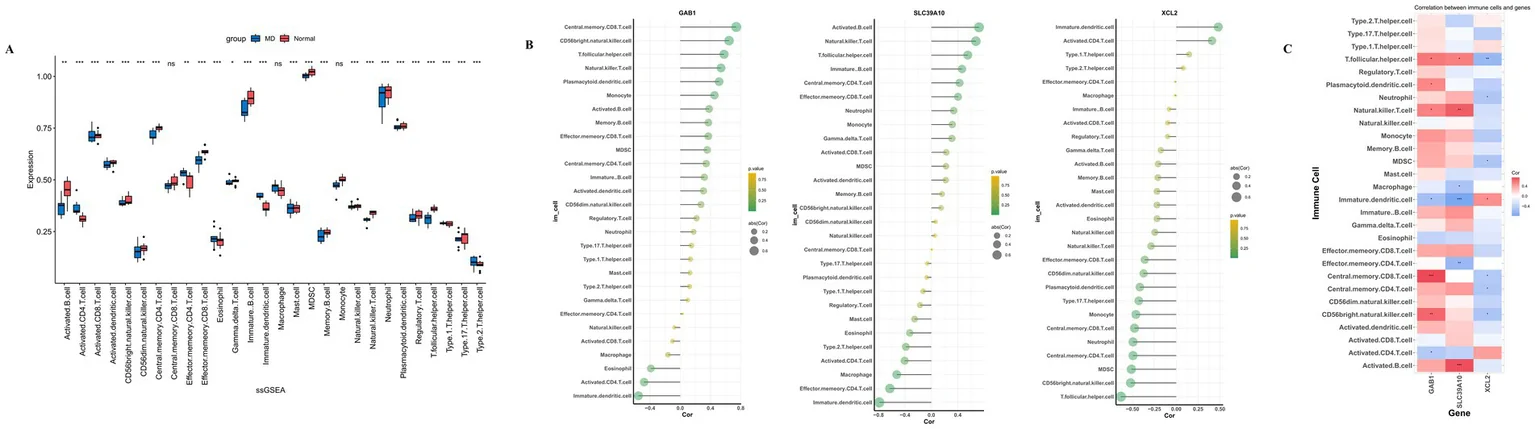
Immune-cell signature enrichment and functional programs associated with *SLC39A10, GAB1,* and *XCL2*. **(A)** Single-sample gene set enrichment analysis-derived scores for 28 predefined immune-cell transcriptional signatures in peripheral-blood samples from patients with Meniere’s disease and healthy controls. Box plots compare the relative signature-enrichment scores between groups. These scores represent relative transcriptional enrichment and should not be interpreted as direct measurements of absolute immune-cell abundance. **(B)** Correlations between the expression of *SLC39A10*, *GAB1*, and *XCL2* and the enrichment scores of the 28 immune-cell signatures. **(C)** Heat map summarizing candidate-gene–immune-signature correlations.

We next placed the three prioritized candidate genes within the broader peripheral immune context of Meniere’s disease. ssGSEA of the GSE202657 expression matrix generated relative enrichment scores for 28 predefined immune-cell transcriptional signatures. Several signatures showed between-group variation, including signatures representing activated and memory lymphocyte populations, dendritic-cell subsets, monocytes, and myeloid-derived suppressor cells (Figure 5A). Because these scores reflect relative transcriptional enrichment rather than direct cell counts, they were interpreted as indicators of immune-cell-associated expression programs rather than absolute cellular abundance.

Correlation analysis between immune-cell signature enrichment scores and gene expression demonstrated structured relations (Figure 5B). *SLC39A10* and *GAB1* were positively correlated with T follicular helper cells and central-memory CD8^+^ T cells, but negatively correlated with immature dendritic cells. *XCL2* showed the opposite pattern, being positively associated with NK-cell subsets and negatively associated with certain activated T-cell populations. These mirror-image correlations suggested that *SLC39A10*, *GAB1*, and *XCL2* do not act in isolation; instead, they occupy axes of T-cell, NK-cell and dendritic-cell interactions that are collectively altered in Meniere’s disease (Figure 5C).

### 3.7 Pathway programs associated with *SLC39A10, GAB1*, and *XCL2*

To dissect the pathway context of each key gene, we stratified patients with Meniere’s disease into high- and low-expression groups (median split) in GSE202657 for *SLC39A10, GAB1* and *XCL2.* We performed differential expression analysis and ranked genes by log₂ fold change. GSEA against the MSigDB c2.kegg.v7.4.symbols collection revealed distinct pathway signatures for each gene.

In *GAB1*-high samples, pathways related to glycosaminoglycan biosynthesis (heparan sulfate) and ubiquitin-mediated proteolysis were significantly enriched (nominal *p* < 0.05), suggesting a link between *GAB1* and extracellular-matrix homeostasis and proteostasis. *SLC39A10*-high samples were enriched for immune and stress-response pathways, consistent with the function of zinc import in amplifying innate and adaptive signaling. *XCL2*-high samples showed enrichment of chemokine and cytotoxic-effector programs, in line with its function in NK–dendritic cooperation. Together, these analyses reinforce the view that the three genes coordinate distinct yet converging aspects of immune activation, tissue protection, and cell–cell communication in Meniere’s disease (Supplementary Figures S2A–C).

### 3.8 Regulatory networks of key genes

**Figure 6:**
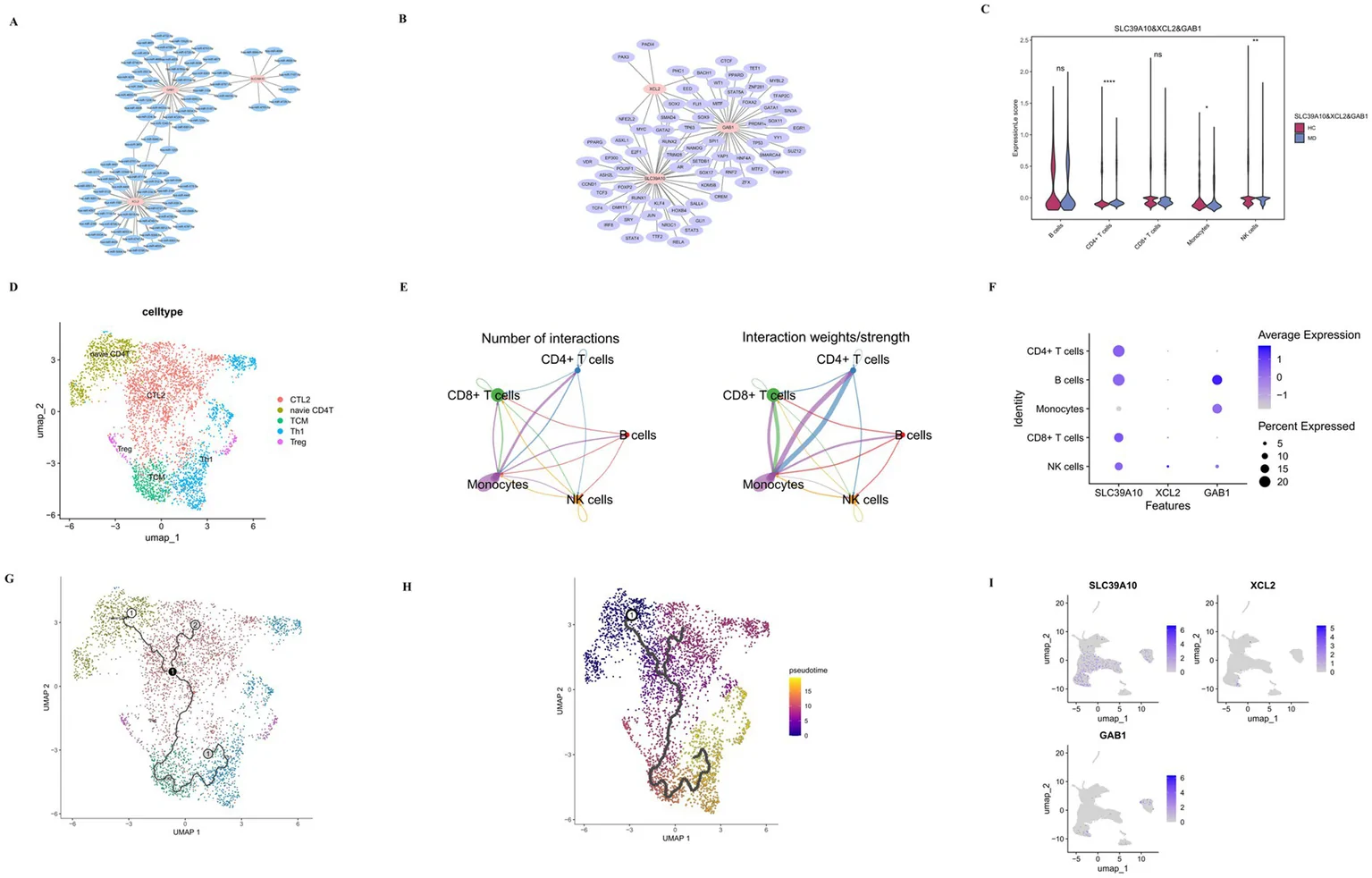
Regulatory networks and CD4^+^ T-cell remodeling along the zinc transport–immune axis. Gene–miRNA interaction network for *SLC39A10, GAB1,* and *XCL2* constructed from MiRTarBase **(A)**. Central pink nodes denote the three key genes; blue nodes correspond to miRNAs predicted or validated to target each gene. Transcription factor (TF)–gene interaction network obtained from ENCODE/ChEA **(B)**. Pink nodes represent *SLC39A10, GAB1,* and *XCL2*; surrounding blue nodes are upstream TFs potentially regulating their expression. **(C)** GSVA scores of the SLC39A10/GAB1/XCL2 gene set across major immune-cell populations in MD and control samples. **(D)** UMAP embedding of CD4^+^ T cells from scRNA-seq, sub-clustered into naïve CD4 T cells, central memory T cells (TCM), Th1 cells, regulatory T cells (Treg), and cytotoxic-like CD4 T cells (CTL2). CellChat-inferred communication networks among major immune-cell populations **(E)**. Edge width represents the inferred interaction number or communication strength, as indicated. Dot plot showing average expression (color) and fraction of expressing cells (dot size) for *SLC39A10, XCL2*, and *GAB1* across major immune-cell types **(F)**. Monocle-based pseudotime trajectories of CD4^+^ T-cell subsets projected onto the UMAP embedding **(G)**. Black lines denote inferred differentiation paths starting from naïve CD4^+^ T cells. Pseudotime coloring of the same trajectories, with early cells in dark blue and late cells in yellow, illustrating two main differentiation branches from naïve CD4^+^ T cells toward TCM/Th1 and CTL2-like states **(H)**. Feature plots depicting the distribution and intensity of *SLC39A10, XCL2*, and *GAB1* expression across the CD4^+^ T-cell manifold, highlighting preferential expression along specific pseudotime branches **(I)**. (The GSVA analysis shown in panel C and the CellChat analysis shown in panel E were performed independently; no formal statistical association between gene-set activity scores and CellChat-derived communication metrics was tested).

To assess gene regulation, we integrated transcription factor (TF) and microRNA (miRNA) interaction data for *SLC39A10*, *GAB1,* and *XCL2*. Using ENCODE and MiRTarBase, we constructed TF–gene and miRNA–gene interaction networks and visualized them in NetworkAnalyst (Figures 6A,B).

*SLC39A10* was predicted to be regulated by miR-885-3p and miR-4689, and by TFs including *NFE2L2* and *VDR*. *GAB1* connected to miR-320a-3p and miR-204-3p, and TFs such as *AR* and *SMAD4*. *XCL2* was linked to miR-4655-5p and miR-6798-5p, and TFs such as *PAX3* and *NFE2L2.* These networks nominated multiple upstream regulatory nodes, both transcriptional and post-transcriptional, that may converge on *SLC39A10-*, *GAB1-*, and *XCL2-*centred pathways in Meniere’s disease. These predicted regulatory networks provide candidate upstream contexts for *SLC39A10*, *GAB1*, and *XCL2* and suggest that their expression-related programs may intersect with stress-response, developmental, and immune-regulatory processes. These network-level associations require experimental validation.

### 3.9 Single-cell mapping localizes the prioritized candidate-gene program to CD4^+^ T cells and NK cells

We next determined at single-cell resolution the immune-cell compartments in which the key gene programs are most active. GSVA scores for a gene set comprising *SLC39A10*, *GAB1,* and *XCL2* were computed for each cell in the scRNA-seq dataset. Enrichment scores were globally higher in MD than in controls and varied across lineages (Figure 6C). Among the major cell types, CD4^+^ T cells, monocytes, and NK cells exhibited the most pronounced separation, with CD4^+^ T cells showing the clearest difference between MD and controls.

Given this signal, we focused on CD4^+^ T cells and performed reclustering. This analysis resolved five transcriptionally distinct CD4^+^ T-cell subsets: naïve CD4 T cells, CTL2-like cytotoxic cells, central memory (TCM) cells, Th1 cells, and regulatory T (Treg) cells (Figure 6D). *SLC39A10* expression was broadly distributed but most prominent in CD4^+^ T-cell and NK-cell subsets, *GAB1* was enriched in B cells and monocytes, and *XCL2* was strongly concentrated in NK cells (Figure 6F). Thus, the expression-related program comprising *SLC39A10*, *GAB1*, and *XCL2* was most evident in CD4^+^ T-cell and NK-cell compartments, with additional contributions from monocytes and B cells. These findings establish cellular localization but do not demonstrate a direct mechanistic interaction among the three genes.

### 3.10 Exploratory group-level cell–cell communication analysis

CellChat analysis of ligand–receptor interactions revealed that CD4^+^ T cells act as major communication hubs in peripheral blood, forming numerous connections with B cells, CD8^+^ T cells, monocytes, and NK cells (Figure 6E). The group-level CellChat networks displayed differences in the inferred communication patterns between the MD and control groups, particularly among CD4^+^ T cells and other major immune-cell lineages. Because cells from different participants were aggregated within each group before communication inference, these patterns were interpreted descriptively and were not subjected to participant-level statistical comparison. The CellChat and GSVA findings provided complementary but analytically independent views of peripheral immune alterations in Meniere’s disease. CellChat characterized inferred ligand–receptor communication among immune-cell populations, whereas GSVA summarized the relative activity of the *SLC39A10/GAB1/XCL2* gene set across these populations. Because no direct statistical association between gene-set activity and CellChat-derived communication metrics was tested, these parallel findings should not be interpreted as evidence that activation of the candidate gene program directly drives altered intercellular communication.

### 3.11 Pseudotime trajectories reveal altered CD4^+^ T-cell differentiation

Pseudotime analysis with monocle reconstructed potential differentiation and activation trajectories of CD4^+^ T-cell subsets. Naïve CD4 T cells occupied early pseudotime positions with high stem-like potential. CTL2-like cells were enriched at intermediate pseudotime, and TCM and Th1 cells were concentrated at later stages (Figures 6G,H). This arrangement suggests that CD4^+^ T cells follow at least two differentiation routes: a trajectory from naïve cells through CTL2-like intermediates to TCM/Th1 effector states, and a second route from naïve cells through CTL2-like states toward alternative effector or terminal phenotypes. Along these trajectories, expression of *SLC39A10* tended to increase, whereas *GAB1* and *XCL2* showed more restricted patterns (Figure 6I), consistent with dynamic re-balancing of zinc transport and immune-regulatory signaling during T-cell activation.

### 3.12 Validation of key genes in an independent clinical cohort

**Figure 7:**
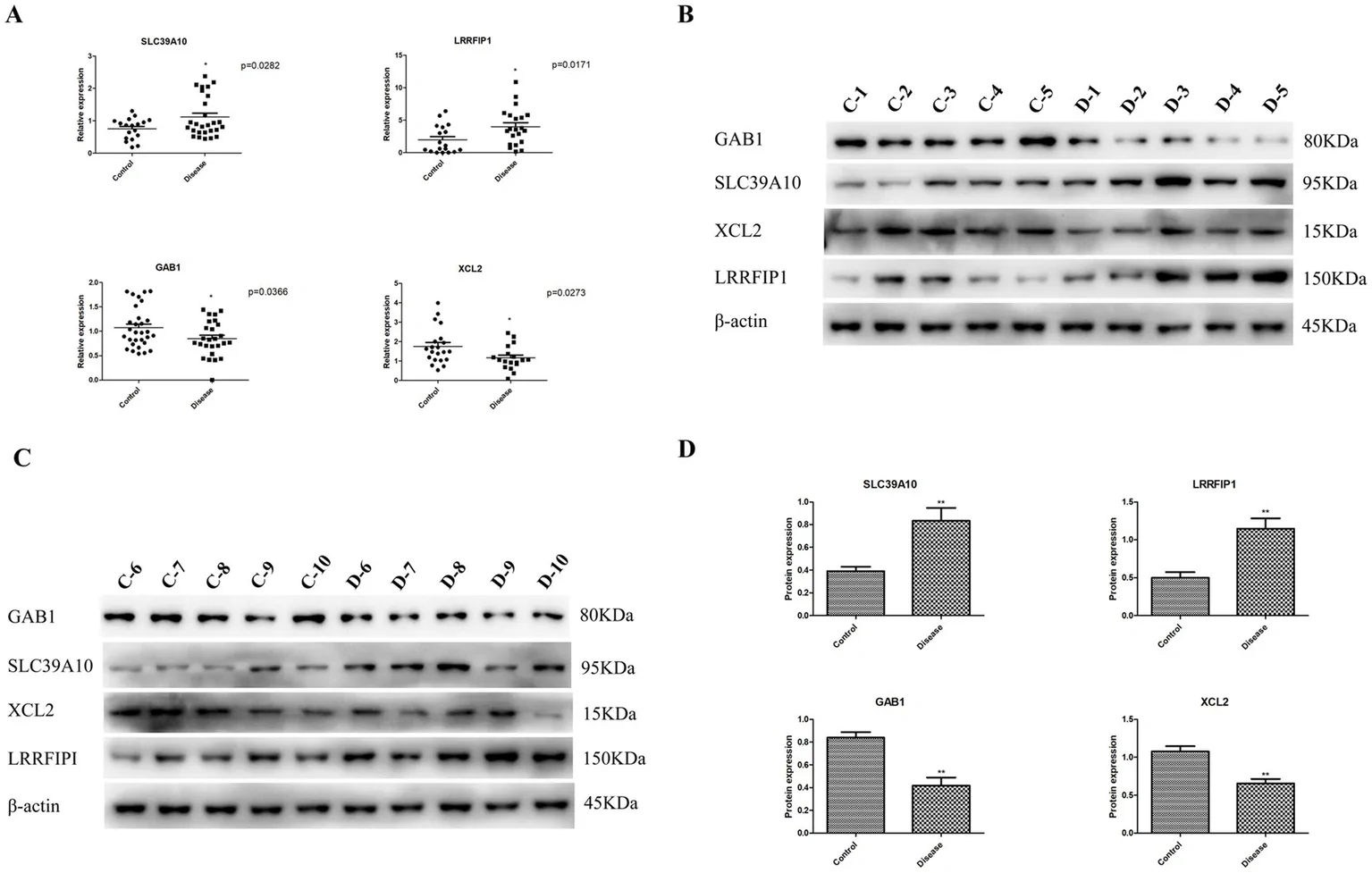
Independent transcript- and protein-level evaluation of *SLC39A10*, *GAB1*, and *XCL2* in peripheral blood. **(A)** Quantitative real-time PCR (qPCR) analysis of relative mRNA expression levels of *SLC39A10, LRRFIP1, GAB1*, and *XCL2* in peripheral blood buffy-coat samples from an independent cohort of patients with Meniere’s disease (MD) and healthy controls. Each dot represents one subject; horizontal lines indicate the median and interquartile range. **(B)** Representative immunoblots of GAB1, SLC39A10, XCL2, and LRRFIP1 protein levels in buffy-coat samples from control (C1–C5) and MD (D1–D5) individuals; β-actin serves as a loading control. **(C)** Immunoblots for an expanded cohort of controls (C6–C10) and MD cases (D6–D10), confirming consistent changes in protein abundance. **(D)** Quantification of protein expression based on densitometric analysis of blots in **(B,C)**, normalized to β-actin. Bars show mean ± s.e.m.; *p*-values were calculated using two-sided statistical tests comparing MD with controls (*p < 0.05, **p < 0.01).

The 10 patients with Meniere’s disease in the independent validation cohort had disease durations ranging from 1 to 7 years. Eight patients had unilateral disease and two had bilateral disease. The recorded stage distribution comprised two patients with stage I disease, two with stage II disease, four with stage III disease, and two with stage IV disease. Attack frequency and recent disease course varied considerably across participants: some patients continued to experience several attacks per year or weekly episodes, whereas four reported no obvious attacks for approximately 6 months to 2 years following treatment. Management included lifestyle modification, glucocorticoid treatment during acute attacks, low-dose diuretics, and intermittent intratympanic dexamethasone or gentamicin. Blood samples were collected between May 2024 and August 2025, and no patient had a documented inflammatory or autoimmune phenotype. Detailed individual-level characteristics are provided in Supplementary Table S5 and summarized in Table 4. To independently evaluate the computationally prioritized genes, we measured the expression of *SLC39A10*, *LRRFIP1*, *GAB1*, and *XCL2* in peripheral blood samples from an independent cohort comprising 10 patients with Meniere’s disease and 10 healthy controls. Quantitative PCR confirmed that *SLC39A10* and *LRRFIP1* mRNA levels were significantly increased in MD, whereas *GAB1* and *XCL2* mRNA levels were significantly decreased (Figure 7A).

Western blot further corroborated these changes at the protein level (Figures 7B–D). SLC39A10 and LRRFIP1 protein abundances were higher in MD samples, whereas GAB1 and XCL2 protein levels were lower in MD than in controls. These concordant transcript- and protein-level changes provided independent support for the MD-associated expression patterns of *SLC39A10*, *GAB1*, and *XCL2*, although the small cohort size and absence of adjustment for demographic or clinical covariates should be considered when interpreting these findings.

Collectively, these multi-layered findings prioritize *SLC39A10*, *GAB1*, and *XCL2* as candidate genes associated with Meniere’s disease and place their expression-related programs within CD4^+^ T-cell and NK-cell compartments. Together with the established biological roles of these genes, the results support a putative zinc transport–immune framework that may be relevant to Meniere’s disease. However, the functional relationships among *SLC39A10* expression, intracellular zinc availability, immune-cell behavior, and disease pathogenesis remain to be experimentally established.

**Table 4:** Individual clinical characteristics of patients with Meniere’s disease in the independent validation cohort.

**Patient ID**	**Disease duration**	**Laterality**	**Recorded stage**	**Attack frequency and recent course**	**Treatment history/Status**	**Blood sampling**	**Documented inflammatory or Autoimmune phenotype**
MD-01	Left ear: 7 years; right ear: 4 years	Bilateral	IV	Initially approximately one attack every 6 months; subsequently progressed to weekly attacks	Initially received oral glucocorticoids and betahistine; currently receives intermittent intratympanic dexamethasone	May 2024	None documented
MD-02	4 years	Left	III	Initially approximately five attacks per year; no obvious attacks during the past year	Glucocorticoid therapy during acute attacks; currently receiving low-dose oral diuretic therapy	October 2024	None documented
MD-03	3 years	Right	II	Approximately two attacks per year	Lifestyle modification and low-dose oral diuretic therapy	September 2024	None documented
MD-04	2.5 years	Left	II	Two attacks within 6 months	Lifestyle modification; glucocorticoid therapy during attacks	October 2024	None documented
MD-05	1 year	Left	I	Three attacks during the first year	Lifestyle modification; glucocorticoid therapy during attacks	October 2024	None documented
MD-06	4 years	Left	I	Initially four major attacks within one year; no obvious attacks for nearly 2 years after treatment	Lifestyle modification; glucocorticoid therapy during attacks	January 2025	None documented
MD-07	6 years	Right	III	Initially one major attack per year; no attacks during the past year	Lifestyle modification; glucocorticoid therapy during attacks; intermittent intratympanic dexamethasone	March 2025	None documented
MD-08	2 years	Right	III	Initially four attacks within 1 year; no attacks during the past 6 months	Lifestyle modification; glucocorticoid therapy during attacks	August 2025	None documented
MD-09	3.5 years	Right	III	Approximately two to three attacks per year	Lifestyle modification; glucocorticoid therapy during attacks; intermittent intratympanic gentamicin	June 2025	None documented
MD-10	7 years	Bilateral	IV	Approximately three to four attacks per year	Intermittent intratympanic dexamethasone	April 2025	None documented

The exact interval between the most recent vertigo attack and blood sampling was not consistently available; therefore, active-attack versus interictal status at the time of sampling could not be uniformly assigned. Treatment information reflects the available treatment history or status recorded in the clinical files and does not necessarily indicate treatment immediately preceding blood collection. “None documented” indicates that no inflammatory or autoimmune phenotype was recorded in the available clinical documentation.

### 3.13 Hypothesis-generating model linking prioritized candidate genes to peripheral immune dysregulation and inner-ear vulnerability

Together, these findings motivated a hypothesis-generating model in which altered expression of *SLC39A10*, *GAB1*, and *XCL2* may be associated with peripheral immune dysregulation and increased inner-ear vulnerability in Meniere’s disease. This model integrates the genetic associations, bulk and single-cell transcriptomic patterns, immune-signature enrichment, inferred intercellular communication, and independent expression-level evidence obtained in the present study with the previously reported biological functions of these genes. Within this framework, altered *SLC39A10* expression may be related to changes in zinc-associated cellular signaling, whereas reduced *GAB1* and *XCL2* expression may be associated with impaired cytoprotective, immune-regulatory, and immune-coordination programs. These molecular patterns may coexist with altered CD4^+^ T-cell and NK-cell states and with changes in inferred ligand–receptor communication within the peripheral immune compartment.

**Figure 8:**
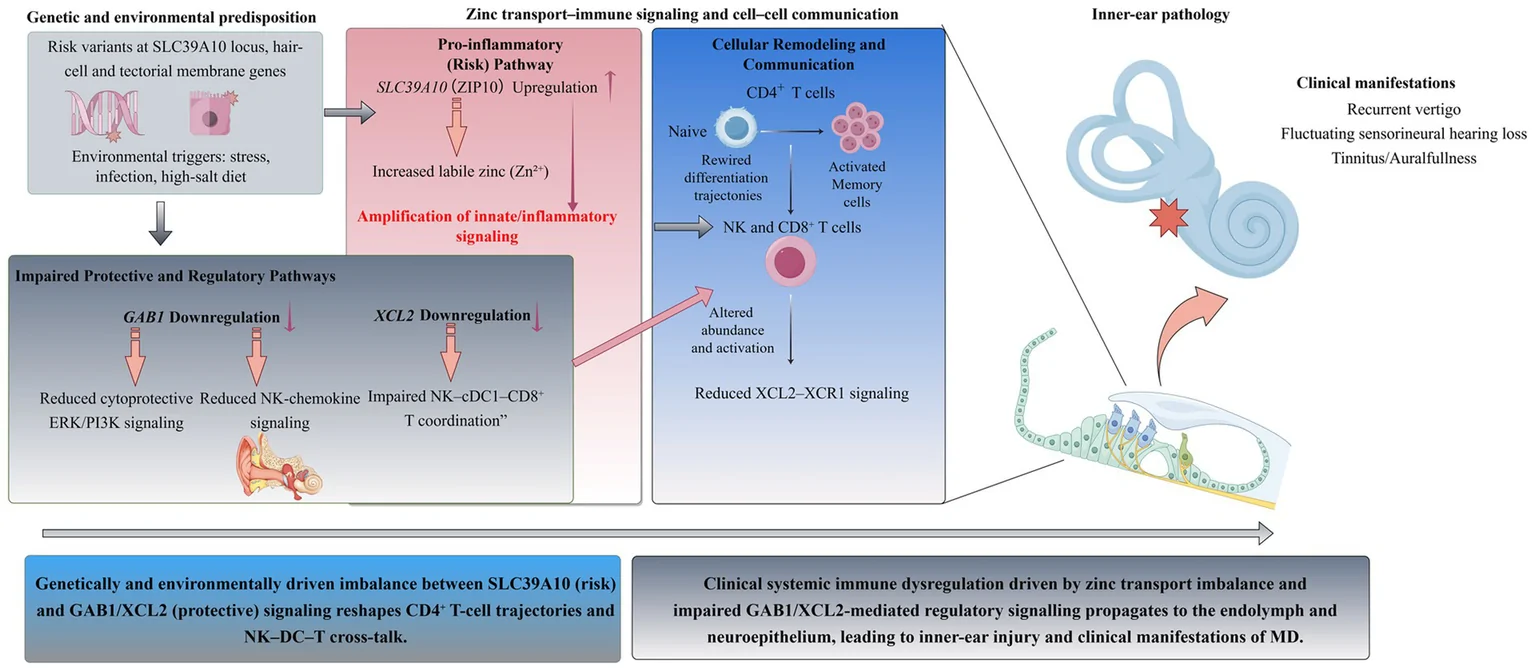
Proposed hypothetical working model of a putative zinc transport–immune framework in Meniere’s disease. This schematic summarizes the hypothesis generated from the integrative genomic, transcriptomic, and single-cell analyses performed in this study. Genetic regulation of *SLC39A10,* together with environmental exposures such as stress, infection, and dietary factors, may be associated with altered *SLC39A10* expression and zinc-related signaling. Reduced expression of *GAB1* and *XCL2* may be associated with impaired cytoprotective and immune-coordination programs. These molecular alterations may coexist with changes in CD4^+^ T-cell and NK-cell states and with altered inferred ligand–receptor communication in peripheral blood. Such systemic immune alterations could potentially influence inner-ear homeostasis and contribute to endolymphatic hydrops and auditory–vestibular dysfunction. The proposed relationships have not been functionally validated, and the figure should therefore be interpreted as a hypothesis-generating working model rather than an experimentally established pathogenic mechanism.

However, the present study did not directly measure intracellular zinc concentrations, zinc-transport activity, zinc-responsive signaling, or the functional consequences of experimentally manipulating *SLC39A10*, *GAB1*, or *XCL2*. Moreover, the temporal and mechanistic relationships between these peripheral molecular alterations, immune-cell remodeling, and inner-ear pathological changes remain unresolved. The proposed framework should therefore be interpreted as a candidate mechanistic hypothesis rather than as an experimentally established pathogenic pathway. Accordingly, *SLC39A10*, *GAB1*, and *XCL2*, together with their associated immune-cell programs, may represent candidate molecular markers and experimental targets for future functional validation rather than established biomarkers or therapeutic targets (Figure 8).

## 4 Discussion

In this integrative genomic and single-cell study, we prioritized *SLC39A10*, *GAB1*, and *XCL2* as candidate genes associated with Meniere’s disease and mapped their expression-related programs to specific peripheral immune-cell contexts. By combining bulk transcriptomics, scRNA-seq, blood eQTLs, MD GWAS summary statistics, and independent clinical validation, we identified a convergent set of 202 candidate genes, obtained supportive MR evidence for three prioritized genes, and confirmed concordant transcript- and protein-level changes. Collectively, these findings support a putative zinc transport–immune framework linking the expression of *SLC39A10*, *GAB1*, and *XCL2* to peripheral immune alterations. However, the study does not establish that altered *SLC39A10* expression produces abnormal zinc influx or that the proposed framework directly causes immune dysregulation or inner-ear injury.

Our findings extend and refine a growing body of work implicating immune and inflammatory mechanisms in Meniere’s disease pathogenesis. Clinical and epidemiological studies have shown co-occurrence of Meniere’s disease with autoimmune and allergic diseases, increased circulating autoantibodies and immune complexes, and partial clinical responses to corticosteroids and other immunomodulators in subsets of patients, supporting the presence of immune-mediated endotypes (9). Recent single-cell and multiomic studies from the López-Escámez research group provide important context for interpreting the peripheral immune alterations observed in Meniere’s disease. Using mass-cytometry-based single-cell immune profiling, Flook et al. identified two patient clusters distinguished by cytokine responses, serum IgE levels, and immune-cell composition, including a subgroup characterized by a type 2/allergic inflammatory phenotype and reduced CD56^dim NK-cell abundance (37). Cruz-Granados et al. subsequently integrated single-cell RNA sequencing with single-cell chromatin-accessibility profiling and identified two immune profiles among patients with Meniere’s disease: a relatively inactive profile and a monocyte-driven profile characterized by transcriptional programs related to responses to biotic stimuli (38). These studies collectively indicate that peripheral immune dysregulation in Meniere’s disease is heterogeneous rather than uniformly distributed across all patients.

Notably, the GSE269117 scRNA-seq dataset reanalyzed in the present study was generated as part of the multiomic investigation by Cruz-Granados et al. (38). Our findings involving monocyte-, NK-cell-, and CD4^+^ T-cell-associated transcriptional programs are consistent with the broader concept that distinct innate- and adaptive-immune programs may predominate in different patient subgroups. However, because we did not reproduce the original participant-level immune clustering or analyze the paired scATAC-seq dataset, individual participants in the present reanalysis cannot be assigned to the relatively inactive or monocyte-driven immune profiles described in the original study (37).

Complementary evidence from studies of inner-ear tissue, endolymphatic sac fluid, and animal models has further suggested inflammatory changes within the endolymphatic compartment and cochlea. In agreement with these observations, our ssGSEA-based immune-signature analysis indicated differences in innate- and adaptive-immune-cell-associated transcriptional programs, while the scRNA-seq analysis further localized relevant expression patterns to B cells, CD4^+^ and CD8^+^ T cells, monocytes, and NK cells.

By integrating WGCNA, bulk differential expression, scRNA-seq-derived cell-type marker information, blood cis-eQTL–based Mendelian randomization, and colocalization analyses, we moved beyond descriptive association and prioritized *SLC39A10*, *GAB1*, and *XCL2* as genes whose genetically predicted expression is associated with MD risk. The directional concordance between MR estimates and observed differential expression, together with independent validation at both transcript and protein levels, strengthens the relevance of these genes to disease-associated pathways, although it does not by itself establish direct causality at the functional level.

*SLC39A10 (ZIP10)* provides a biologically plausible link between systemic immunity, cellular stress responses, and inner-ear vulnerability. Members of the SLC39 family import Zn^2+^ into the cytosol and shape labile zinc pools that function as second messengers in immune and neural cells (39–41). Experimental studies have shown that SLC39A10 is important for hematopoietic stem-cell maintenance and B-cell receptor signal tuning, thereby supporting humoral immunity and stress resistance. More broadly, dysregulated zinc signaling modulates apoptosis, barrier integrity, and inflammatory responses in multiple tissues (41–43). In the auditory system, both zinc deficiency and excess free zinc have been implicated in sensorineural hearing loss. In noise-induced hearing loss, preclinical work has shown that pathological surges of unbound zinc occur in cochlear hair-cell synapses, and that genetic disruption of vesicular zinc release or pharmacologic zinc chelation can mitigate auditory dysfunction and enhance recovery (44). Against this background, our observation that *SLC39A10* is genetically and transcriptionally upregulated in MD, broadly expressed across peripheral lymphocyte subsets, and associated with enrichment of activated immune-cell-associated transcriptional signatures is consistent with a scenario in which heightened zinc influx may amplify innate and adaptive immune responses and lower the threshold for inflammatory injury in susceptible individuals.

In contrast, *GAB1* and *XCL2* may represent protective or counter-regulatory programs within the same broader network. *GAB1* is a multisite docking adaptor that integrates signals from cytokine receptors, growth factors, pattern-recognition receptors, and antigen receptors, thereby modulating MAPK/ERK and PI3K pathways and influencing cell survival, differentiation, and cytokine production (45, 46). In macrophages and dendritic cells, *GAB1* can tune TLR- and RIG-I-associated responses, whereas in endothelial and epithelial contexts it contributes to cytoprotective and angiogenic signaling (47, 48). In our cohort, higher *GAB1* expression was genetically associated with reduced MD risk, yet *GAB1* mRNA and protein levels were significantly decreased in MD. Gene set enrichment indicated that *GAB1*-high patients preferentially engaged pathways related to heparan sulfate biosynthesis and ubiquitin-mediated proteolysis, processes linked to extracellular-matrix integrity and proteostasis. Taken together, these findings suggest that reduced *GAB1* expression may weaken cytoprotective and regulatory circuits, thereby rendering immune and stromal cells more vulnerable to stress and maladaptive inflammation.

*XCL2*, in turn, is a chemokine closely related to XCL1 and forms part of the XCL–XCR1 axis linking NK cells, CD8^+^ T cells, and cross-presenting dendritic cells. Activated NK and CD8^+^ T cells secrete XCL1/2 to recruit XCR1^+^ cDC1s, thereby enabling efficient antigen cross-presentation and cytotoxic immunity in infection, cancer, and autoimmunity (46, 49–51). In our analyses, XCL2 was genetically protective, strongly downregulated in MD, and highly enriched in NK cells. Its inverse correlations with several activated T-cell and dendritic-cell subsets suggest that reduced XCL2 expression may reflect, or contribute to, impaired coordination between NK cells and cDC1s, thereby compromising cytotoxic immune surveillance and downstream adaptive immune responses. Together with the differences observed in NK-cell-associated transcriptional signatures and CD4^+^ T-cell states, these findings raise the possibility that Meniere’s disease may involve not only heightened inflammatory activity but also selective disruption of immune-orchestration pathways that normally help constrain and resolve inflammation.

Our single-cell analyses add further resolution to how these genes are embedded within immune programs. A composite *SLC39A10–GAB1–XCL2* gene set was preferentially enriched in CD4^+^ T cells, monocytes, and NK cells and was significantly altered in Meniere’s disease. Within CD4^+^ T cells, reclustering revealed naïve, CTL2-like cytotoxic, central-memory, Th1, and Treg subsets arranged along pseudotime trajectories consistent with progressive activation and effector differentiation. CD4^+^ T cells also emerged as central nodes in CellChat-inferred communication networks, with MD samples displaying rewired ligand–receptor interactions linking CD4^+^ T cells to multiple partner populations. Importantly, the GSVA and CellChat results represent complementary but analytically independent observations, as no direct association between gene-set activity and CellChat-derived communication metrics was formally tested. Their co-occurrence should therefore be considered hypothesis-generating rather than evidence that the *SLC39A10/GAB1/XCL2* program directly regulates intercellular communication. These observations align with recent reports of immunological heterogeneity in Meniere’s disease, including autoinflammatory and type 2-skewed CD4^+^ T-cell phenotypes and altered Th1/Th2/Th17/Treg balance (9, 11). They are also consistent with MR-based evidence implicating inflammatory cytokine signaling in MD risk (10), together with emerging evidence of altered Th17/Treg-related immune balance in MD. In our dataset, *SLC39A10* and *GAB1* tracked positively with T follicular helper cells and negatively with immature dendritic cells, whereas *XCL2* showed the opposite pattern, suggesting that these genes may lie along axes of Tfh–DC crosstalk and effector–regulatory balance relevant to Meniere’s disease and other immune-mediated inner-ear disorders (10, 12, 52).

Meniere’s disease is unlikely to represent a single immunological entity. Previous clinical and molecular studies have proposed distinct inflammatory, autoinflammatory, and autoimmune-associated endotypes, with substantial interindividual variation in cytokine profiles, CD4^+^ T-cell polarization, innate immune activation, and treatment responsiveness (9–11). Within this framework, the peripheral immune-cell-associated transcriptional patterns observed in the present study may be more prominent in an immunologically active subgroup rather than being uniformly present across all patients with Meniere’s disease. The reported occurrence of vestibular dysfunction in systemic autoimmune and rheumatologic diseases further supports a broader relationship between systemic immune dysregulation and inner-ear dysfunction (38, 53). The independent validation cohort was itself clinically heterogeneous, encompassing unilateral and bilateral disease, stages I–IV, disease durations of 1–7 years, and markedly different attack patterns and treatment histories. Although this heterogeneity may increase the clinical breadth of the validation, the small cohort precluded reliable stratified analyses according to stage, laterality, attack frequency, or treatment status. However, standardized attack status, treatment history, inflammatory endotyping, and accessible individual-level demographic and clinical metadata were not available across the study cohorts. We were therefore unable to perform formal clinical subgroup analyses or adjust the validation results for potential demographic and clinical confounders.

On the basis of the convergent but indirect evidence obtained in this study, we propose a hypothetical working model rather than a functionally established pathway. From a systems perspective, our findings are compatible with a model in which genetic and environmental influences may converge on peripheral immune regulation and inner-ear homeostasis. Altered *SLC39A10* expression may be associated with changes in zinc-related signaling, whereas reduced *GAB1* and *XCL2* expression may accompany weakened cytoprotective and immune-coordination programs. However, the present data do not establish increased zinc influx, direct regulation of immune-cell activity by these genes, or a causal link between peripheral immune alterations and inner-ear pathology. The proposed relationships therefore remain hypothetical and require direct functional validation.

Our findings also have preliminary translational implications. First, the combined expression pattern of *SLC39A10, GAB1*, and *XCL2* warrants further evaluation as a potential peripheral molecular signature of Meniere’s disease. However, its diagnostic performance, stability across disease stages, and applicability to distinct clinical or immunological endotypes remain to be established in larger, independent cohorts. Second, the upstream regulatory nodes identified in the TF–miRNA networks, including NFE2L2, VDR, AR, and selected miRNAs, may provide additional entry points for investigating how stress, hormonal, and redox-responsive programs intersect with immune remodeling in MD. Third, preclinical work in noise-induced hearing loss has shown that pharmacologic chelation of excess free zinc can protect cochlear synapses and improve functional recovery, suggesting that finely tuned modulation of zinc signaling, rather than indiscriminate supplementation or depletion, may be beneficial. Similarly, therapies aimed at rebalancing Th-cell/Treg programs are already in clinical use or clinical translation in autoimmune and allergic diseases, whereas strategies that more directly restore NK–DC–T-cell immune coordination remain more exploratory and may merit future evaluation in carefully selected Meniere’s disease subgroups. Any such approaches, however, will require rigorous experimental validation and controlled clinical trials; our results should be regarded as hypothesis-generating rather than as immediate therapeutic recommendations.

Several limitations of our study warrant consideration. First, we performed all transcriptomic and scRNA-seq analyses using peripheral blood, which may only partially reflect immune processes within the endolymphatic sac, cochlea, and vestibular organs. Although studies of vestibular tissue and inner-ear fluids have identified overlapping immune and stress-related pathways, direct profiling of inner-ear immune and epithelial compartments in Meniere’s disease remains limited. Second, the GWAS and eQTL datasets used for MR were derived predominantly from European-ancestry populations and may not capture genetic architectures in other ancestries, thereby limiting generalizability. Third, although MR reduces confounding relative to observational analyses, it relies on assumptions about instrument validity, and our colocalization analyses did not provide strong evidence that the eQTL and MD risk signals share single causal variants at the nominated loci. The lack of strong colocalization evidence suggests that the underlying genetic architecture may involve multiple regulatory variants, context-dependent effects, or trans-regulation that is not fully captured by current reference panels and analytical methods. Fourth, although SLC39A10 is a well-established zinc transporter, the present study did not directly measure intracellular zinc concentrations, zinc-transport activity, or zinc-responsive signaling in patient-derived cells. Nor did we experimentally manipulate SLC39A10, GAB1, or XCL2 to determine their effects on immune-cell activation, differentiation, or intercellular communication. The CellChat analysis was performed using cells aggregated within disease groups rather than independently for each participant. Therefore, the inferred communication patterns may be influenced by group-level aggregation, and their reproducibility across individual participants could not be evaluated. Consequently, the proposed zinc transport–immune framework is based on convergent genomic, transcriptomic, and prior biological evidence and should be regarded as a hypothesis requiring functional validation. Fifth, the discovery transcriptomic cohort and the independent clinical validation cohort were relatively small, comprising 9 patients with Meniere’s disease and 9 controls and 10 patients with Meniere’s disease and 10 controls, respectively. These sample sizes may increase susceptibility to sampling variability, unstable effect estimates, and overestimation of between-group differences. Although additional RLE, principal-component, and sample-clustering analyses did not identify an obvious sample-level technical outlier, no explicit experimental batch variable was available in the GSE202657 metadata. Therefore, the possibility of residual or unrecorded technical variation cannot be completely excluded. In addition, the validation cohort was not individually matched by age or sex, and the absence of accessible individual-level demographic data precluded adjusted analyses for these potential confounders. Although the concordant direction of transcript- and protein-level changes across the discovery and validation cohorts supports the reproducibility of *SLC39A10*, *GAB1*, and *XCL2*, the stability, diagnostic performance, and generalizability of these candidates require confirmation in larger, prospectively characterized multicenter cohorts. Sixth, although additional clinical characteristics of the validation cohort—including disease duration, laterality, recorded clinical stage, attack frequency, treatment history, blood-sampling month, and documented inflammatory or autoimmune phenotypes—were available and are now reported, several clinically relevant variables remained incomplete. In particular, the exact interval between the most recent vertigo attack and blood sampling and the temporal relationship between recent treatment exposure and sample collection were not consistently documented. Individual-level age and sex data were also unavailable for adjusted analyses. Consequently, residual clinical and demographic confounding cannot be excluded, and we could not determine whether the observed molecular differences were preferentially associated with active attacks, interictal disease, treatment exposure, or a particular clinical or immunological endotype. Future prospective studies should integrate molecular sampling with standardized documentation of attack status, treatment exposure, and clinical phenotype. Finally, the cross-sectional nature of the public transcriptomic datasets and the independent validation cohort precludes determination of whether the observed molecular alterations precede disease onset, fluctuate between active attacks and remission, or represent secondary adaptations to established disease. Longitudinal sampling will therefore be required to establish their temporal stability and relationship to disease activity.

Further work should address these limitations by (i) incorporating longitudinal sampling, larger multi-ancestry cohorts, and more granular clinical phenotyping; (ii) extending multi-omic profiling to inner-ear tissues and cerebrospinal fluid where feasible; and (iii) performing functional experiments that manipulate *SLC39A10*, *GAB1*, and *XCL2* in relevant immune and inner-ear models. Single-cell multi-omics, including joint profiling of chromatin accessibility, DNA methylation, and surface proteomes, could further resolve the regulatory circuits controlling zinc transport and immune responses in Meniere’s disease. Ultimately, functional experiments, clinical endotyping, and interventional studies will be required to determine whether the putative zinc transport–immune framework proposed here represents a reproducible component of Meniere’s disease biology and a tractable target for disease modification, or whether it reflects one of several convergent molecular patterns associated with the disease.

## Data Availability

The datasets presented in this study can be found in online repositories. The names of the repository/repositories and accession number(s) can be found in the article/Supplementary material. The analysis code used in this study is publicly available through GitHub at: https://github.com/meibu4385-bot/Jiancheng-Xue. The version corresponding to the present revision is available under commit 7378f30.

## Footnotes

1
https://eQTLgen.org/cis-eQTLs.html

2
https://cran.r-project.org/package=pheatmap

3
https://mrcieu.github.io/TwoSampleMR/index.html

4
https://github.com/chr1swallace/coloc/

5
https://bioconductor.org/packages/monocle/

## Supplementary material

Supplementary Figure S1 Bulk differential-expression analysis and integrative prioritization of Meniere’s disease-associated candidate genes. **(A)** Heat map of representative differentially expressed genes in GSE202657. **(B)** Volcano plot showing differentially expressed genes between MD and control samples. **(C)** Overlap among genes from the three MD-associated WGCNA modules, bulk differentially expressed genes, and scRNA-seq-derived cell-type marker genes, yielding 202 candidate genes for downstream analyses.

Supplementary Figure S2 Gene set enrichment analyses associated with *SLC39A10*, *GAB1*, and *XCL2* expression.

Supplementary Figure S3 Quality-control assessment of the GSE202657 bulk-transcriptomic dataset. **(A)** Distribution of expression values across the 18 samples in the supplied input expression matrix. **(B)** Expression distributions after between-array quantile normalization. **(C)** Relative log expression distributions after normalization. RLE values centered near zero indicate comparable global expression distributions across samples. **(D)** Principal-component analysis of the normalized expression matrix, with samples annotated according to MD or control status. **(E)** Hierarchical clustering of normalized sample-expression profiles using Pearson-correlation distance and average linkage.
