# Supplementary material for: Integrative genomic and single-cell transcriptomic analyses suggest a putative zinc transport–immune axis in Meniere’s disease
Source: Front Neurol. 2026 Sep 8;17:1858821. doi: 10.3389/fneur.2026.1858821 (PMC13595996; doi:10.3389/fneur.2026.1858821)
Supplement: Supplementary file 1 [file Data_Sheet_1.ZIP › 2.10免疫浸润结果/1分组箱式图+显著性P值.pdf]

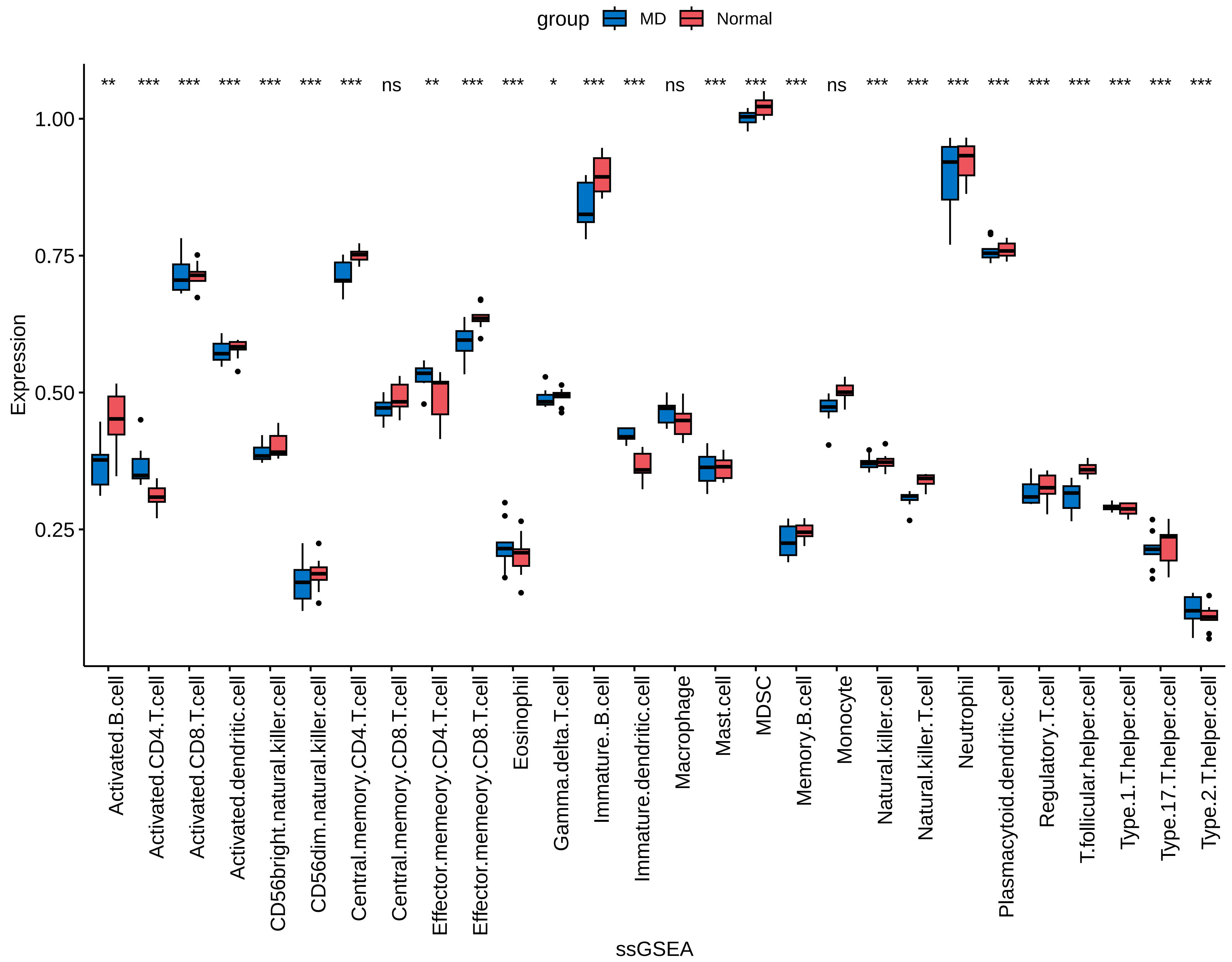

group
MD
Normal
Expression
1.00
0.75
0.50
0.25
** *** *** *** *** *** *** ns ** *** *** * *** *** ns *** *** *** ns *** *** *** *** *** *** *** *** ***
Activated.B.cell
Activated.CD4.T.cell
Activated.CD8.T.cell
Activated.dendritic.cell
CD56bright.natural.killer.cell
CD56dim.natural.killer.cell
Central.memory.CD4.T.cell
Central.memory.CD8.T.cell
Effector.memeory.CD4.T.cell
Effector.memeory.CD8.T.cell
Eosinophil
Gamma.delta.T.cell
Immature..B.cell
Immature.dendritic.cell
Macrophage
Mast.cell
MDSC
Memory.B.cell
Monocyte
Natural.killer.cell
Natural.killer.T.cell
Neutrophil
Plasmacytoid.dendritic.cell
Regulatory.T.cell
T.follicular.helper.cell
Type.1.T.helper.cell
Type.17.T.helper.cell
Type.2.T.helper.cell
ssGSEA
