# Supplementary material for: Integrative genomic and single-cell transcriptomic analyses suggest a putative zinc transport–immune axis in Meniere’s disease
Source: Front Neurol. 2026 Sep 8;17:1858821. doi: 10.3389/fneur.2026.1858821 (PMC13595996; doi:10.3389/fneur.2026.1858821)
Supplement: Supplementary file 1 [file Data_Sheet_1.ZIP › 2.10免疫浸润结果/GAB1+相关性棒棒图.pdf]

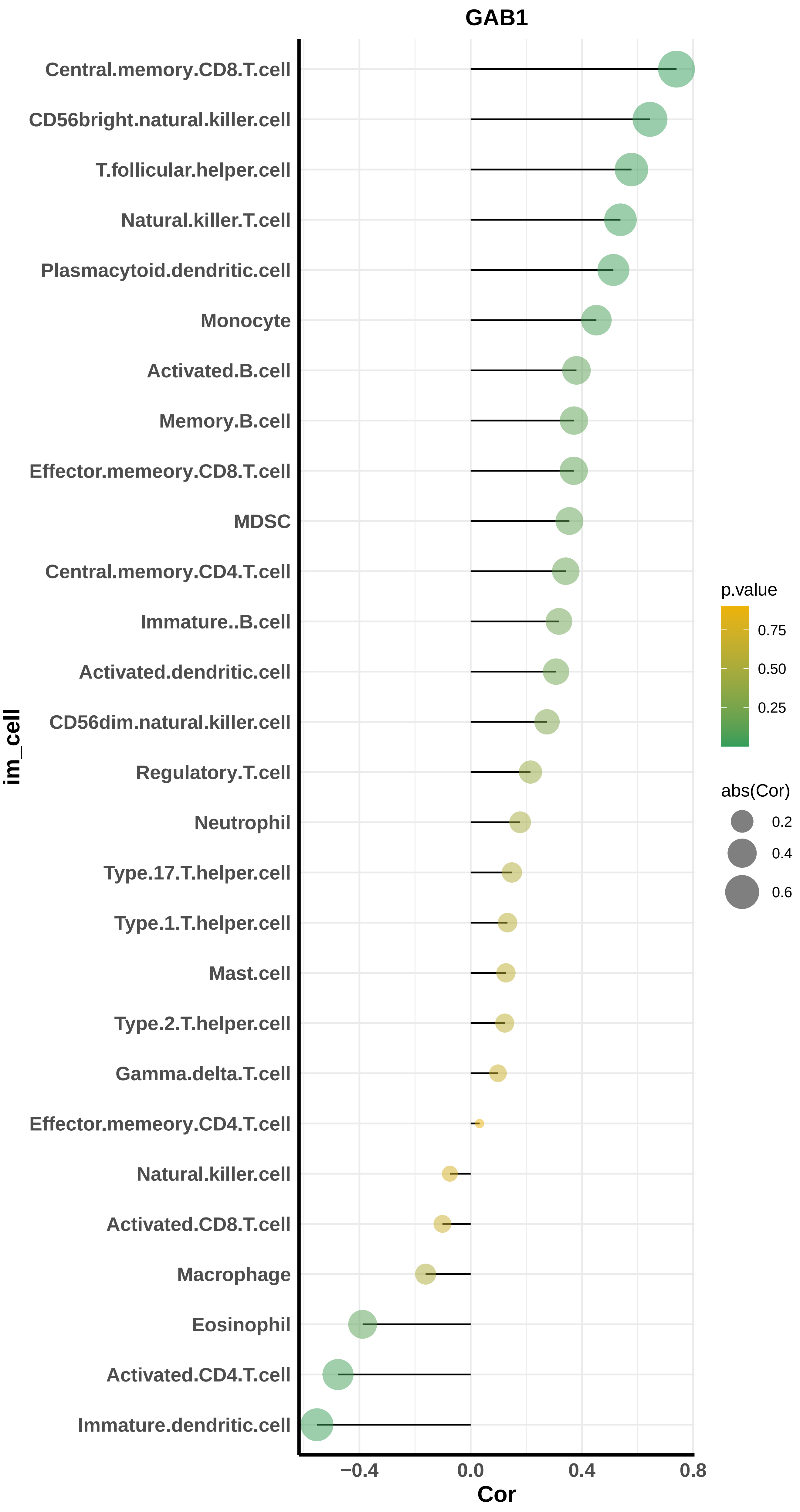

GAB1
Central.memory.CD8.T.cell
CD56bright.natural.killer.cell
T.follicular.helper.cell
Natural.killer.T.cell
Plasmacytoid.dendritic.cell
Monocyte
Activated.B.cell
Memory.B.cell
Effector.memeory.CD8.T.cell
MDSC
Central.memory.CD4.T.cell
Immature..B.cell
Activated.dendritic.cell
CD56dim.natural.killer.cell
Regulatory.T.cell
Neutrophil
Type.17.T.helper.cell
Type.1.T.helper.cell
Mast.cell
Type.2.T.helper.cell
Gamma.delta.T.cell
Effector.memeory.CD4.T.cell
Natural.killer.cell
Activated.CD8.T.cell
Macrophage
Eosinophil
Activated.CD4.T.cell
Immature.dendritic.cell
im_cell
−0.4
0.0
0.4
0.8
Cor
p.value
0.75
0.50
0.25
abs(Cor)
0.2
0.4
0.6
