# Supplementary material for: Integrative genomic and single-cell transcriptomic analyses suggest a putative zinc transport–immune axis in Meniere’s disease
Source: Front Neurol. 2026 Sep 8;17:1858821. doi: 10.3389/fneur.2026.1858821 (PMC13595996; doi:10.3389/fneur.2026.1858821)
Supplement: Supplementary file 1 [file Data_Sheet_1.ZIP › 2.10免疫浸润结果/显著性+相关性矩阵图.pdf]

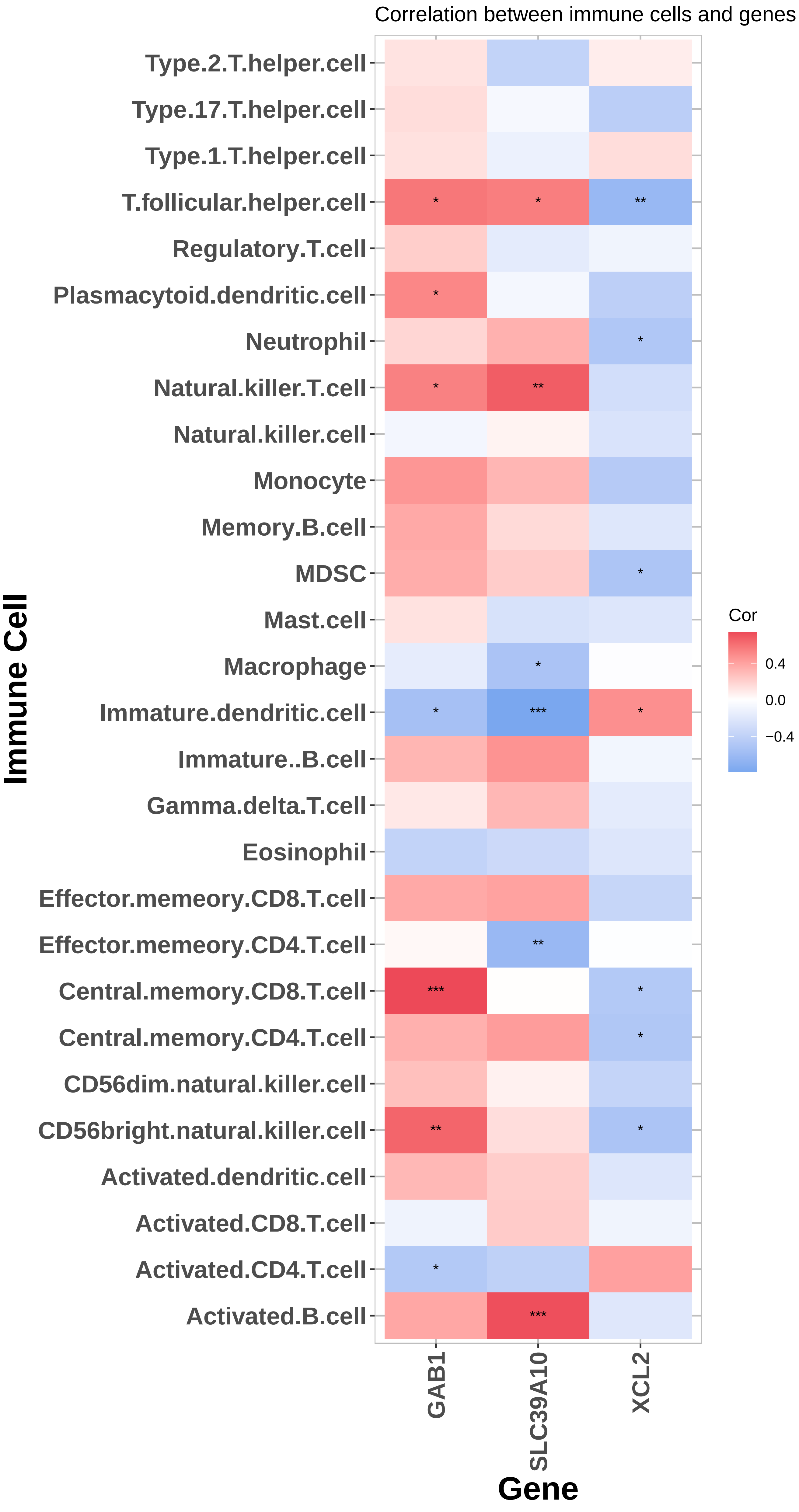
Correlation between immune cells and genes
Immune Cell
Type.2.T.helper.cell
Type.17.T.helper.cell
Type.1.T.helper.cell
T.follicular.helper.cell
Regulatory.T.cell
Plasmacytoid.dendritic.cell
Neutrophil
Natural.killer.T.cell
Natural.killer.cell
Monocyte
Memory.B.cell
MDSC
Mast.cell
Macrophage
Immature.dendritic.cell
Immature..B.cell
Gamma.delta.T.cell
Eosinophil
Effector.memeory.CD8.T.cell
Effector.memeory.CD4.T.cell
Central.memory.CD8.T.cell
Central.memory.CD4.T.cell
CD56dim.natural.killer.cell
CD56bright.natural.killer.cell
Activated.dendritic.cell
Activated.CD8.T.cell
Activated.CD4.T.cell
Activated.B.cell
GAB1
SLC39A10
XCL2
Gene
Cor
0.4
0.0
−0.4
