# Supplementary material for: Integrative genomic and single-cell transcriptomic analyses suggest a putative zinc transport–immune axis in Meniere’s disease
Source: Front Neurol. 2026 Sep 8;17:1858821. doi: 10.3389/fneur.2026.1858821 (PMC13595996; doi:10.3389/fneur.2026.1858821)
Supplement: Supplementary file 1 [file Data_Sheet_1.ZIP › 2.11重要基因的GSEA结果/GAB1_kegg.pdf]

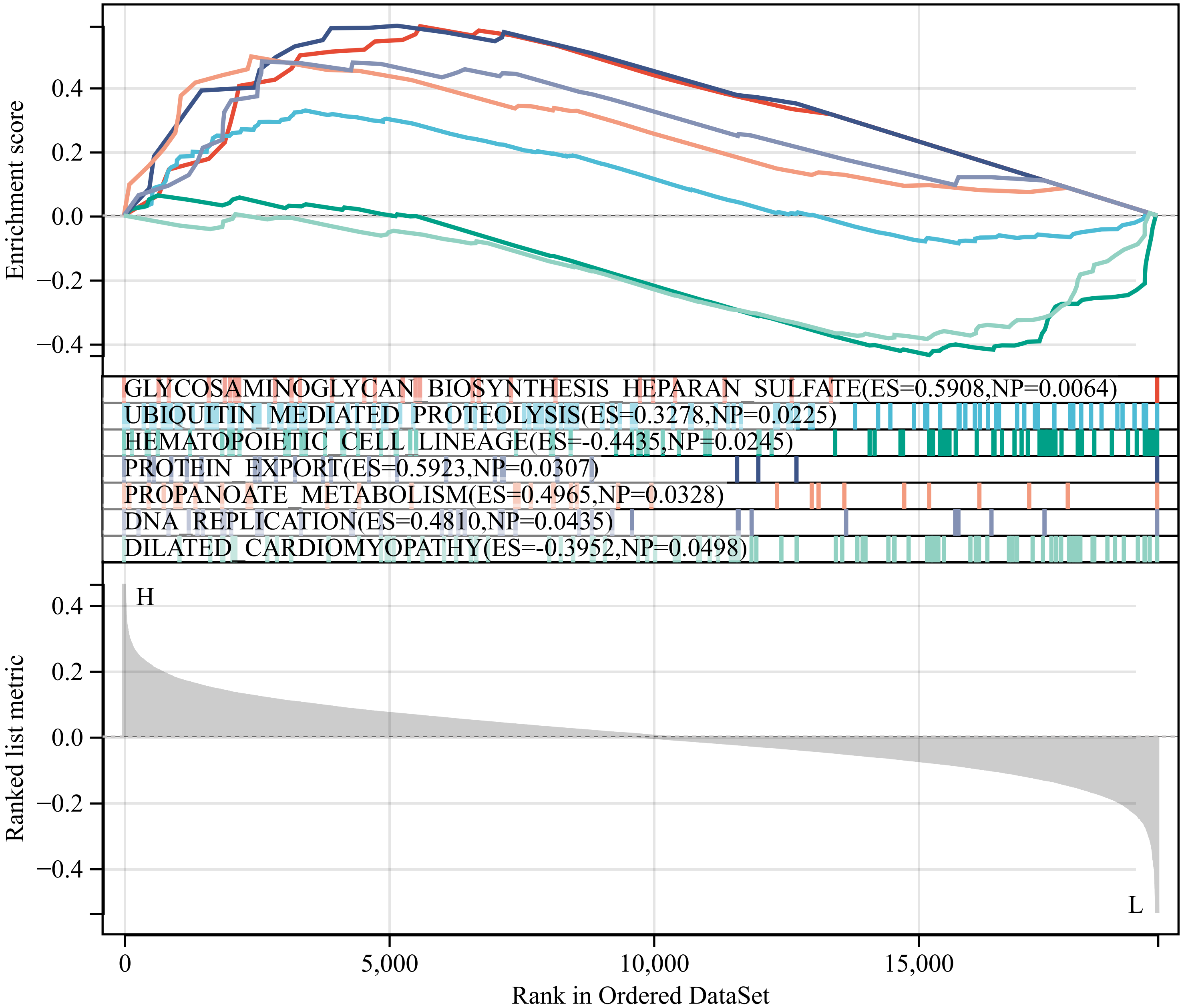

Enrichment score
0.4
0.2
0.0
−0.2
−0.4
GLYCOSAMINOGLYCAN_BIOSYNTHESIS_HEPARAN_SULFATE(ES=0.5908,NP=0.0064)
UBIQUITIN_MEDIATED_PROTEOLYSIS(ES=0.3278,NP=0.0225)
HEMATOPOIETIC_CELL_LINEAGE(ES=-0.4435,NP=0.0245)
PROTEIN_EXPORT(ES=0.5923,NP=0.0307)
PROPANOATE_METABOLISM(ES=0.4965,NP=0.0328)
DNA_REPLICATION(ES=0.4810,NP=0.0435)
DILATED_CARDIOMYOPATHY(ES=-0.3952,NP=0.0498)
Ranked list metric
H
L
0
5,000
10,000
15,000
Rank in Ordered DataSet
