# Supplementary material for: Integrative genomic and single-cell transcriptomic analyses suggest a putative zinc transport–immune axis in Meniere’s disease
Source: Front Neurol. 2026 Sep 8;17:1858821. doi: 10.3389/fneur.2026.1858821 (PMC13595996; doi:10.3389/fneur.2026.1858821)
Supplement: Supplementary file 1 [file Data_Sheet_1.ZIP › 2.11重要基因的GSEA结果/XCL2_kegg.pdf]

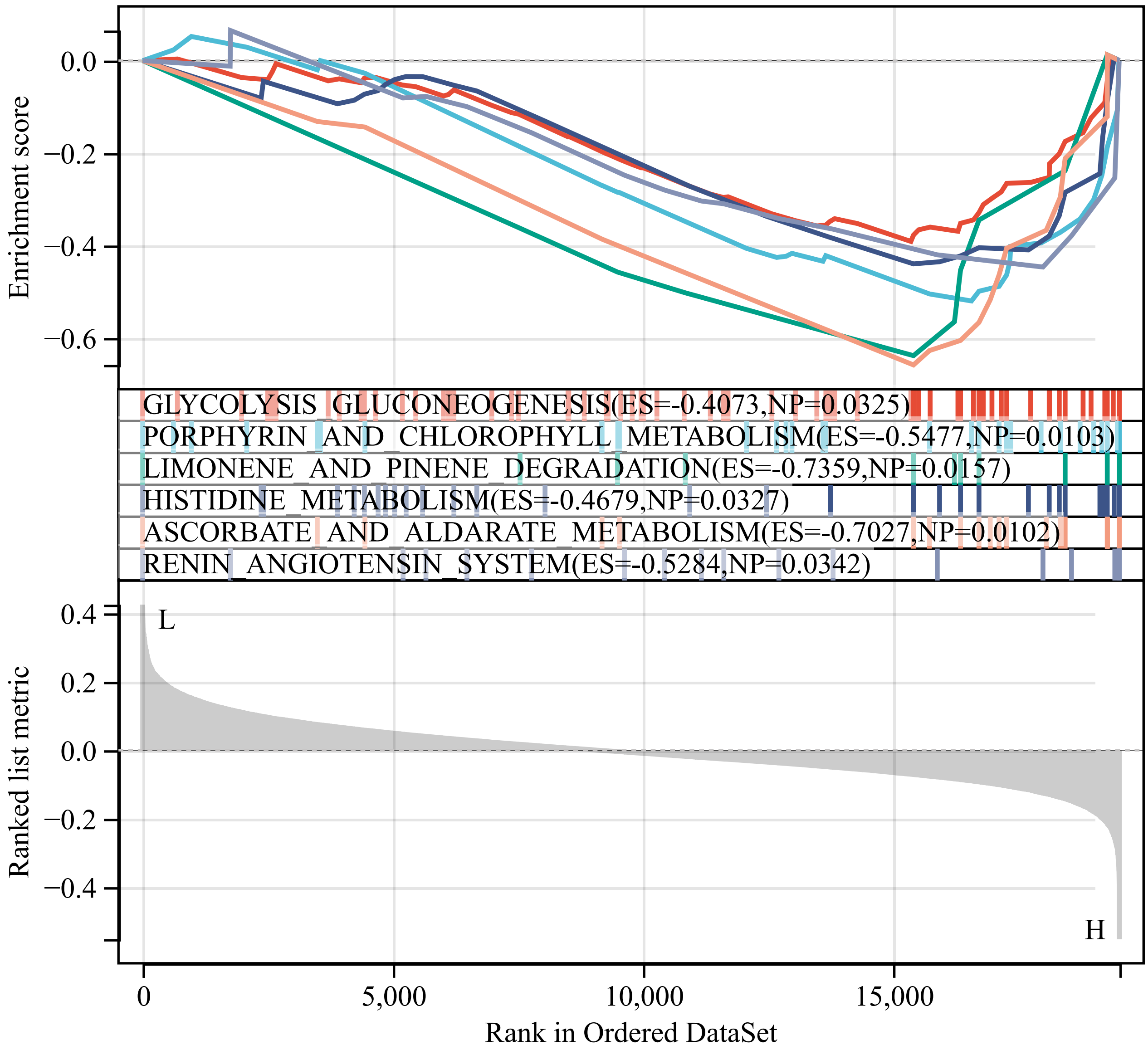
Enrichment score
0.0
−0.2
−0.4
−0.6
GLYCOLYSIS_GLUCONEOGENESIS(ES=-0.4073,NP=0.0325)
PORPHYRIN_AND_CHLOROPHYLL_METABOLISM(ES=-0.5477,NP=0.0103)
LIMONENE_AND_PINENE_DEGRADATION(ES=-0.7359,NP=0.0157)
HISTIDINE_METABOLISM(ES=-0.4679,NP=0.0327)
ASCORBATE_AND_ALDARATE_METABOLISM(ES=-0.7027,NP=0.0102)
RENIN_ANGIOTENSIN_SYSTEM(ES=-0.5284,NP=0.0342)
Ranked list metric
0.4
0.2
0.0
−0.2
−0.4
L
H
0
5,000
10,000
15,000
Rank in Ordered DataSet
