# Supplementary material for: Integrative genomic and single-cell transcriptomic analyses suggest a putative zinc transport–immune axis in Meniere’s disease
Source: Front Neurol. 2026 Sep 8;17:1858821. doi: 10.3389/fneur.2026.1858821 (PMC13595996; doi:10.3389/fneur.2026.1858821)
Supplement: Supplementary file 1 [file Data_Sheet_1.ZIP › 2.12TFs 和 gene-miRNAs 与重要基因的相互作用结果/3GENE_miRNA_Targets.txt.pdf]

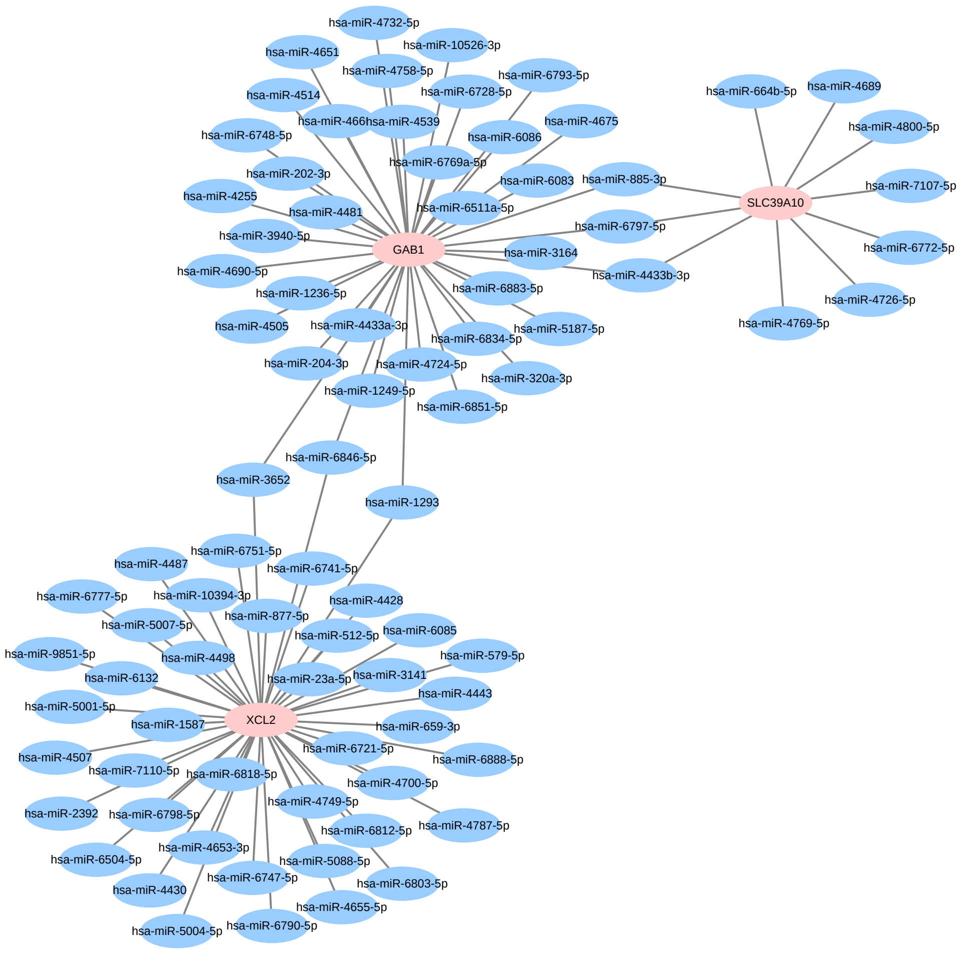
hsa-miR-4732-5p
hsa-miR-4651
hsa-miR-10526-3p
hsa-miR-4758-5p
hsa-miR-6793-5p
hsa-miR-4514
hsa-miR-6728-5p
hsa-miR-466
hsa-miR-4539
hsa-miR-4675
hsa-miR-6748-5p
hsa-miR-6086
hsa-miR-6769a-5p
hsa-miR-202-3p
hsa-miR-6083
hsa-miR-885-3p
hsa-miR-4255
hsa-miR-4481
hsa-miR-6511a-5p
hsa-miR-3940-5p
hsa-miR-6797-5p
GAB1
hsa-miR-3164
hsa-miR-4690-5p
hsa-miR-4433b-3p
hsa-miR-1236-5p
hsa-miR-6883-5p
hsa-miR-4505
hsa-miR-4433a-3p
hsa-miR-5187-5p
hsa-miR-6834-5p
hsa-miR-204-3p
hsa-miR-4724-5p
hsa-miR-320a-3p
hsa-miR-1249-5p
hsa-miR-6851-5p
hsa-miR-6846-5p
hsa-miR-3652
hsa-miR-1293
hsa-miR-664b-5p
hsa-miR-4689
hsa-miR-4800-5p
SLC39A10
hsa-miR-7107-5p
hsa-miR-6772-5p
hsa-miR-4726-5p
hsa-miR-4769-5p
hsa-miR-6751-5p
hsa-miR-4487
hsa-miR-6741-5p
hsa-miR-6777-5p
hsa-miR-10394-3p
hsa-miR-4428
hsa-miR-877-5p
hsa-miR-5007-5p
hsa-miR-6085
hsa-miR-512-5p
hsa-miR-9851-5p
hsa-miR-4498
hsa-miR-579-5p
hsa-miR-6132
hsa-miR-23a-5p
hsa-miR-3141
hsa-miR-4443
hsa-miR-5001-5p
XCL2
hsa-miR-1587
hsa-miR-659-3p
hsa-miR-4507
hsa-miR-6721-5p
hsa-miR-6888-5p
hsa-miR-7110-5p
hsa-miR-6818-5p
hsa-miR-4700-5p
hsa-miR-4749-5p
hsa-miR-2392
hsa-miR-6798-5p
hsa-miR-4787-5p
hsa-miR-6812-5p
hsa-miR-6504-5p
hsa-miR-4653-3p
hsa-miR-5088-5p
hsa-miR-6747-5p
hsa-miR-4430
hsa-miR-6803-5p
hsa-miR-4655-5p
hsa-miR-5004-5p
hsa-miR-6790-5p
