# Supplementary material for: Integrative genomic and single-cell transcriptomic analyses suggest a putative zinc transport–immune axis in Meniere’s disease
Source: Front Neurol. 2026 Sep 8;17:1858821. doi: 10.3389/fneur.2026.1858821 (PMC13595996; doi:10.3389/fneur.2026.1858821)
Supplement: Supplementary file 1 [file Data_Sheet_1.ZIP › 2.12TFs 和 gene-miRNAs 与重要基因的相互作用结果/3GENE_TF.txt.pdf]

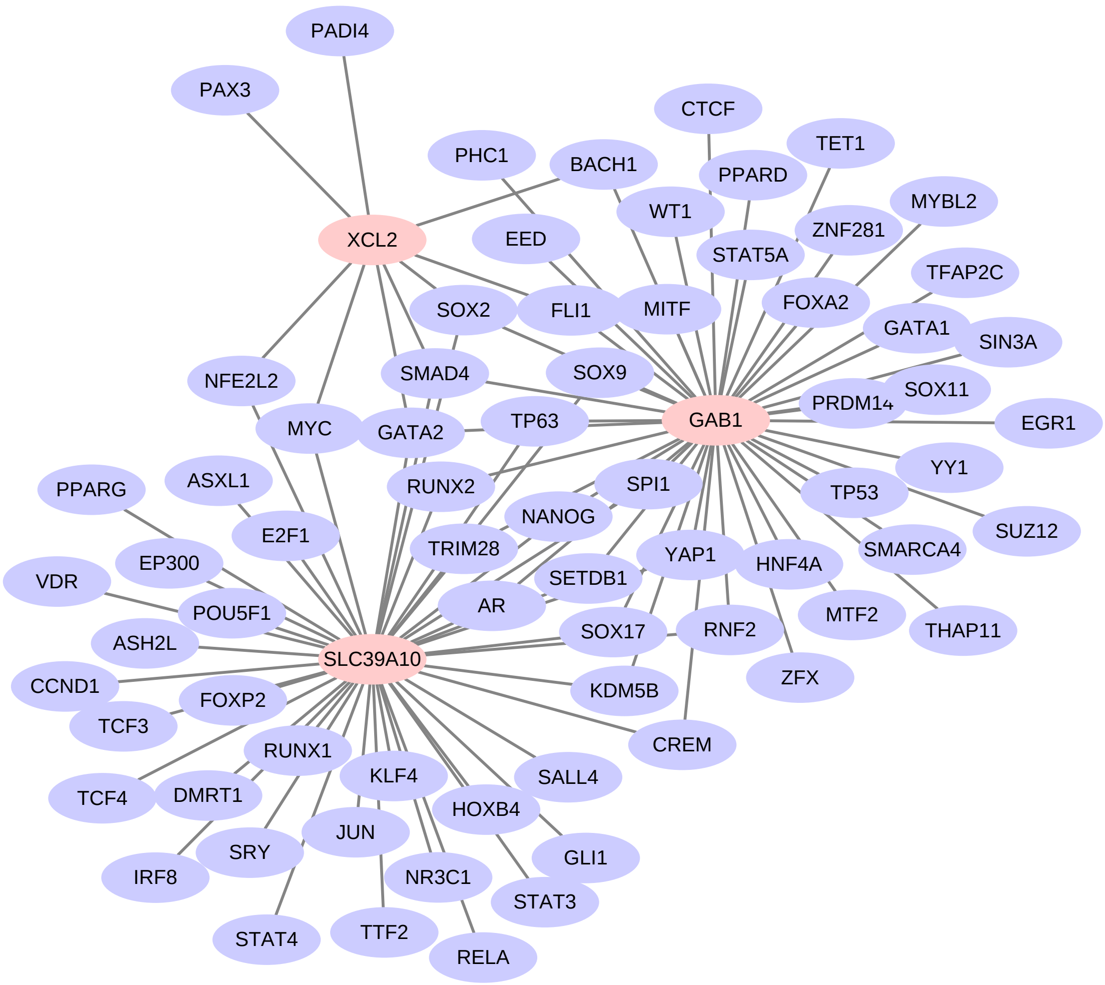
PADI4
PAX3
CTCF
PHC1
BACH1
TET1
PPARD
MYBL2
WT1
ZNF281
XCL2
EED
STAT5A
TFAP2C
SOX2
FLI1
MITF
FOXA2
GATA1
SIN3A
SMAD4
SOX9
SOX11
NFE2L2
PRDM14
GAB1
MYC
GATA2
TP63
EGR1
YY1
ASXL1
RUNX2
SPI1
TP53
PPARG
E2F1
NANOG
SUZ12
TRIM28
SMARCA4
EP300
YAP1
HNF4A
VDR
SETDB1
AR
POU5F1
MTF2
THAP11
ASH2L
SOX17
RNF2
SLC39A10
ZFX
CCND1
KDM5B
FOXP2
TCF3
CREM
RUNX1
SALL4
KLF4
TCF4
DMRT1
HOXB4
JUN
SRY
GLI1
IRF8
NR3C1
STAT3
STAT4
TTF2
RELA
