# Supplementary material for: Integrative genomic and single-cell transcriptomic analyses suggest a putative zinc transport–immune axis in Meniere’s disease
Source: Front Neurol. 2026 Sep 8;17:1858821. doi: 10.3389/fneur.2026.1858821 (PMC13595996; doi:10.3389/fneur.2026.1858821)
Supplement: Supplementary file 1 [file Data_Sheet_1.ZIP › 2.13基因与MD中的免疫相互作用/Rplot02.pdf]

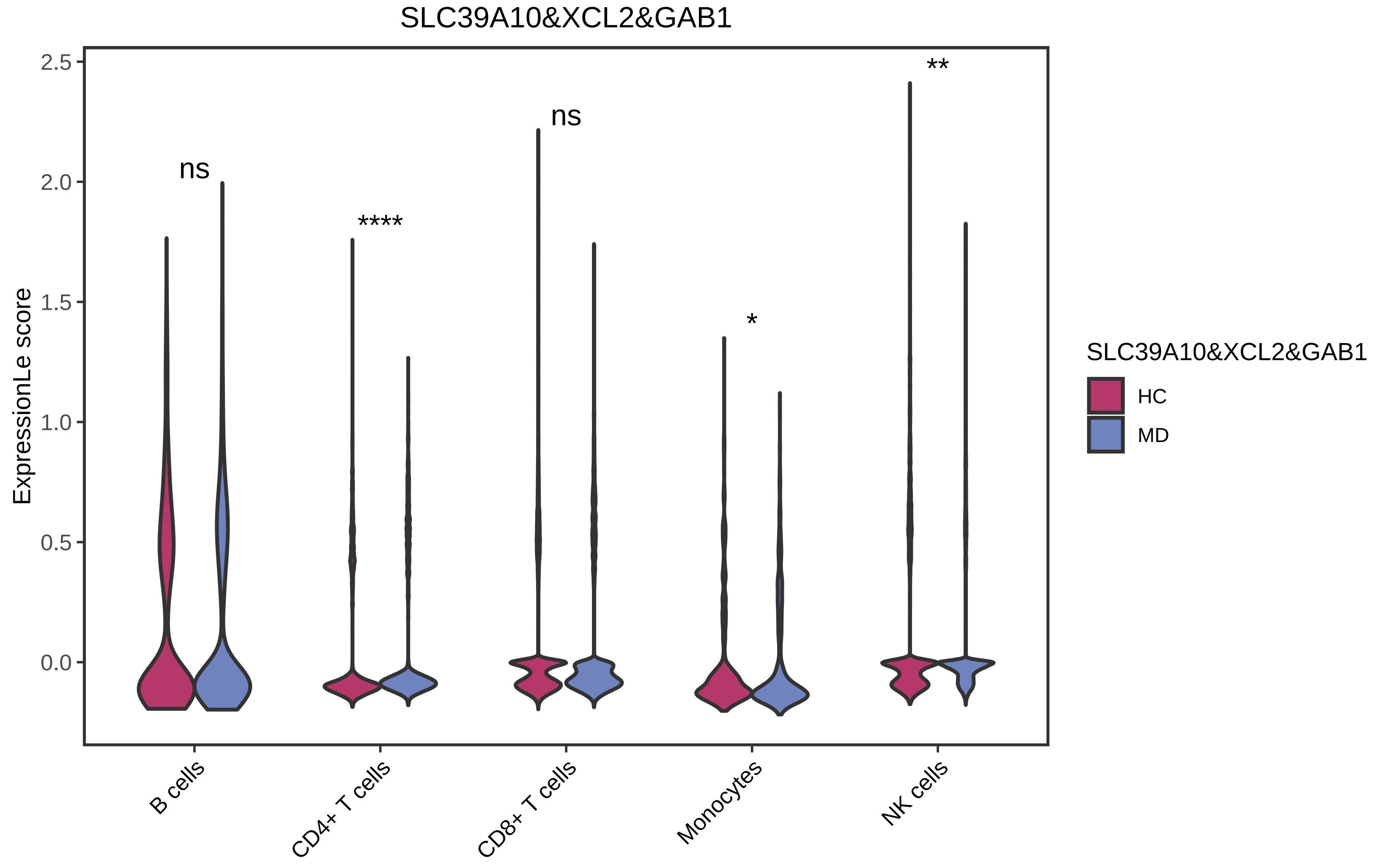

SLC39A10&XCL2&GAB1
ExpressionLe score
2.5
2.0
1.5
1.0
0.5
0.0
ns
****
ns
*
**
B cells
CD4+ T cells
CD8+ T cells
Monocytes
NK cells
SLC39A10&XCL2&GAB1
HC
MD
