# Supplementary material for: Integrative genomic and single-cell transcriptomic analyses suggest a putative zinc transport–immune axis in Meniere’s disease
Source: Front Neurol. 2026 Sep 8;17:1858821. doi: 10.3389/fneur.2026.1858821 (PMC13595996; doi:10.3389/fneur.2026.1858821)
Supplement: Supplementary file 1 [file Data_Sheet_1.ZIP › 2.14scRNA-seq中重点细胞的单细胞亚群分析/CD4MRAKER.pdf]

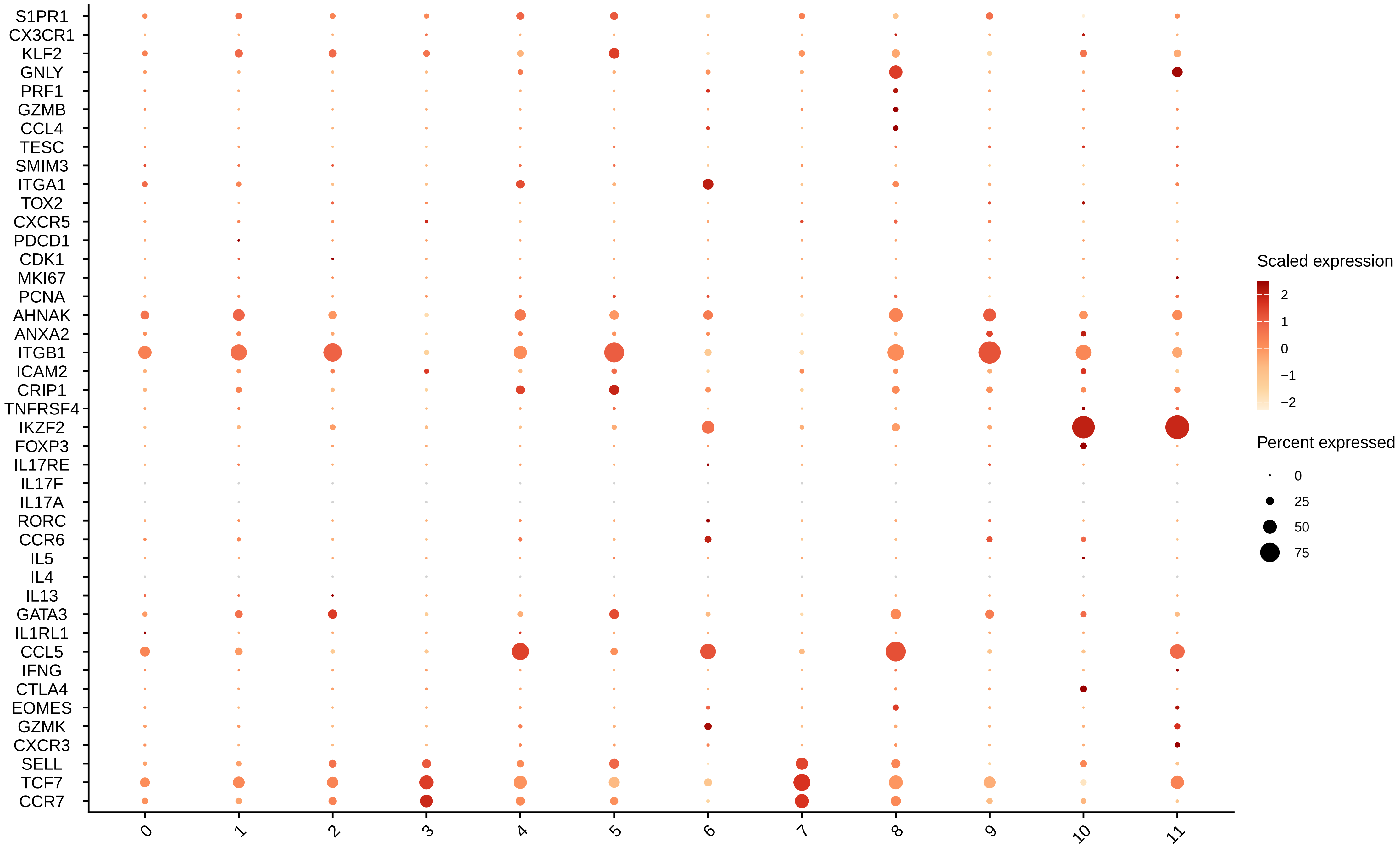
S1PR1
CX3CR1
KLF2
GNLY
PRF1
GZMB
CCL4
TESC
SMIM3
ITGA1
TOX2
CXCR5
PDCD1
CDK1
MKI67
PCNA
AHNAK
ANXA2
ITGB1
ICAM2
CRIP1
TNFRSF4
IKZF2
FOXP3
IL17RE
IL17F
IL17A
RORC
CCR6
IL5
IL4
IL13
GATA3
IL1RL1
CCL5
IFNG
CTLA4
EOMES
GZMK
CXCR3
SELL
TCF7
CCR7
0
1
2
3
4
5
6
7
8
9
10
11
Scaled expression
2
1
0
−1
−2
Percent expressed
0
25
50
75
