# Supplementary material for: Integrative genomic and single-cell transcriptomic analyses suggest a putative zinc transport–immune axis in Meniere’s disease
Source: Front Neurol. 2026 Sep 8;17:1858821. doi: 10.3389/fneur.2026.1858821 (PMC13595996; doi:10.3389/fneur.2026.1858821)
Supplement: Supplementary file 1 [file Data_Sheet_1.ZIP › 2.14scRNA-seq中重点细胞的单细胞亚群分析/CD4注释.pdf]

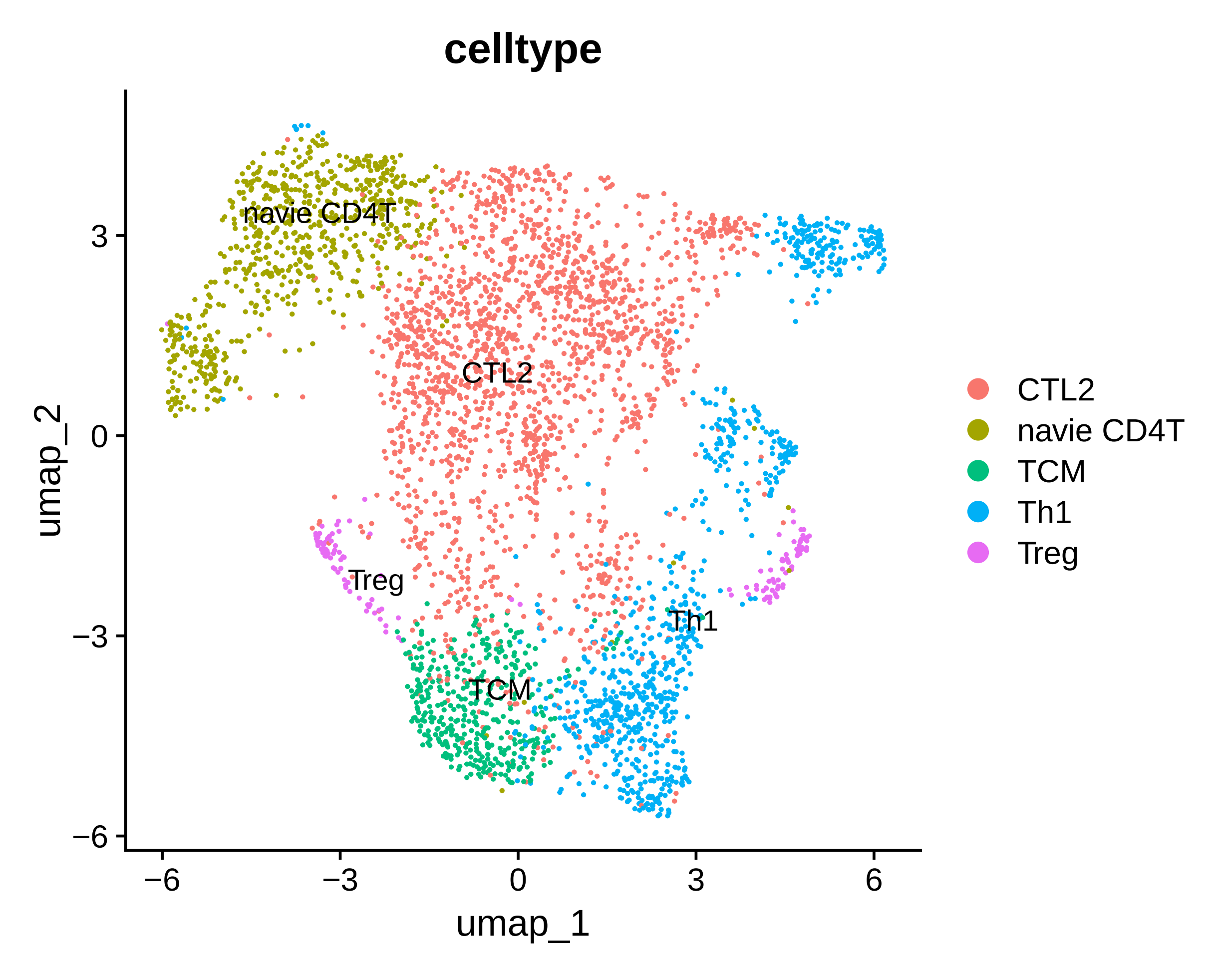

celltype
navie CD4T
CTL2
Treg
Th1
TCM
umap_2
umap_1
3
0
−3
−6
−6
−3
0
3
6
CTL2
navie CD4T
TCM
Th1
Treg
