# Supplementary material for: Integrative genomic and single-cell transcriptomic analyses suggest a putative zinc transport–immune axis in Meniere’s disease
Source: Front Neurol. 2026 Sep 8;17:1858821. doi: 10.3389/fneur.2026.1858821 (PMC13595996; doi:10.3389/fneur.2026.1858821)
Supplement: Supplementary file 1 [file Data_Sheet_1.ZIP › 2.15配体受体相互作用分析/CELL CHAT.pdf]

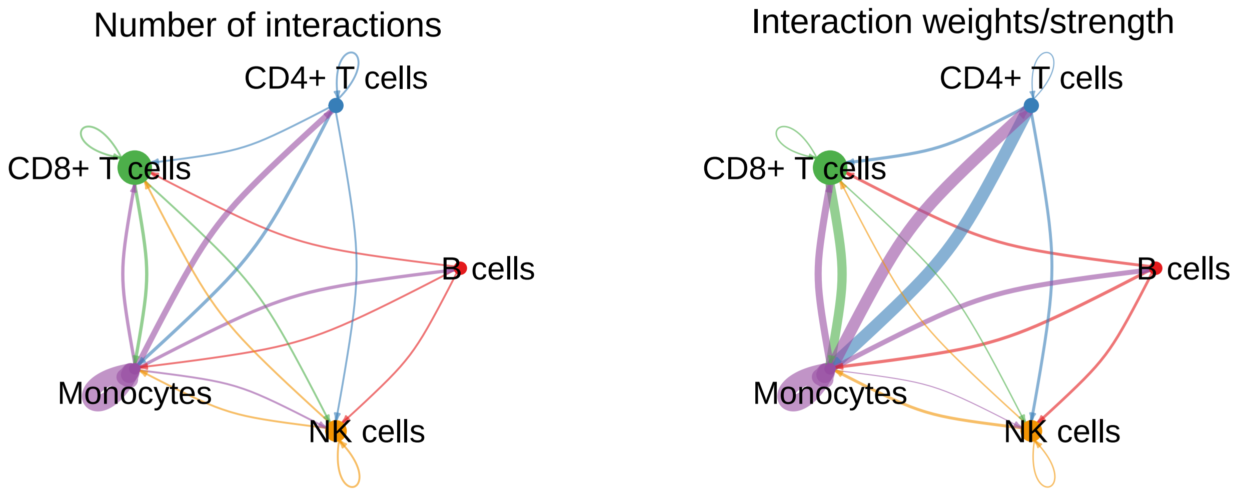
Number of interactions
CD4+ T cells
CD8+ T cells
B cells
Monocytes
NK cells
Interaction weights/strength
CD4+ T cells
CD8+ T cells
B cells
Monocytes
NK cells
