# Supplementary material for: Integrative genomic and single-cell transcriptomic analyses suggest a putative zinc transport–immune axis in Meniere’s disease
Source: Front Neurol. 2026 Sep 8;17:1858821. doi: 10.3389/fneur.2026.1858821 (PMC13595996; doi:10.3389/fneur.2026.1858821)
Supplement: Supplementary file 1 [file Data_Sheet_1.ZIP › 2.15配体受体相互作用分析/HC OUT.pdf]

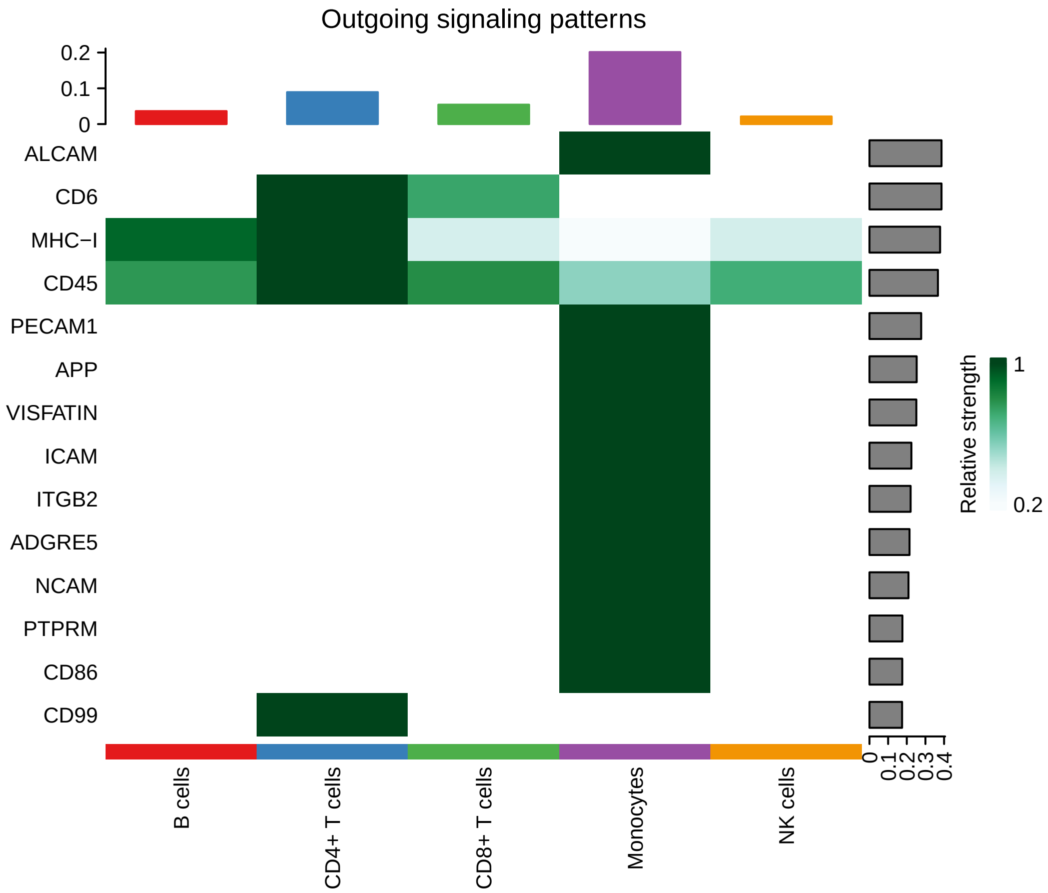

Outgoing signaling patterns
0.2
0.1
0
ALCAM
CD6
MHC−I
CD45
PECAM1
APP
VISFATIN
ICAM
ITGB2
ADGRE5
NCAM
PTPRM
CD86
CD99
B cells
CD4+ T cells
CD8+ T cells
Monocytes
NK cells
0
0.1
0.2
0.3
0.4
Relative strength
1
0.2
