# Supplementary material for: Integrative genomic and single-cell transcriptomic analyses suggest a putative zinc transport–immune axis in Meniere’s disease
Source: Front Neurol. 2026 Sep 8;17:1858821. doi: 10.3389/fneur.2026.1858821 (PMC13595996; doi:10.3389/fneur.2026.1858821)
Supplement: Supplementary file 1 [file Data_Sheet_1.ZIP › 2.16拟时序分析/Rplot(1).pdf]

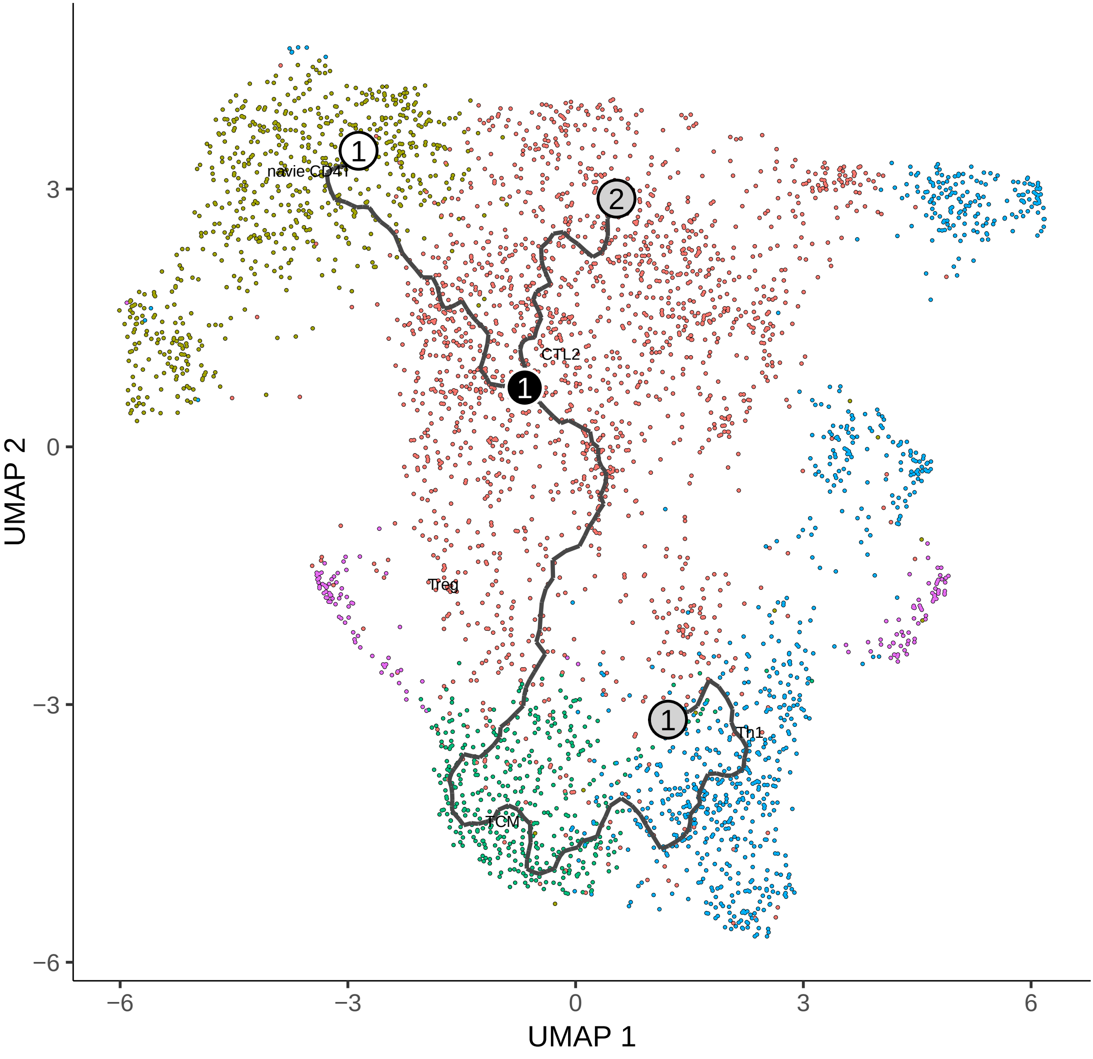

navie CD4T
CTL2
Treg
TCM
Th1
1
2
1
1
3
0
−3
−6
−6
−3
0
3
6
UMAP 1
UMAP 2
