# Supplementary material for: Integrative genomic and single-cell transcriptomic analyses suggest a putative zinc transport–immune axis in Meniere’s disease
Source: Front Neurol. 2026 Sep 8;17:1858821. doi: 10.3389/fneur.2026.1858821 (PMC13595996; doi:10.3389/fneur.2026.1858821)
Supplement: Supplementary file 1 [file Data_Sheet_1.ZIP › 2.17重要基因在细胞中的表达分布/3geneRplot(1).pdf]

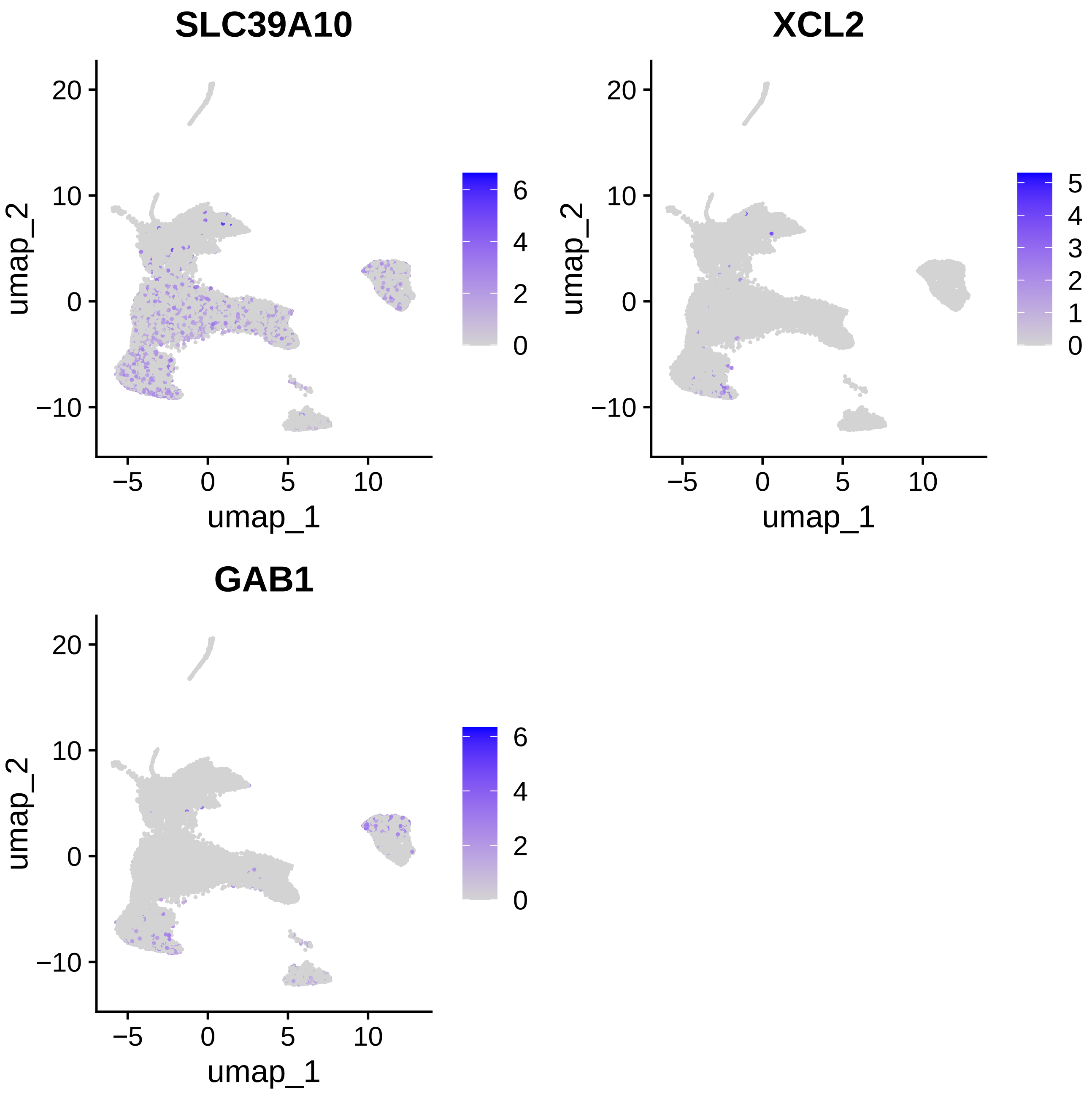
SLC39A10
XCL2
GAB1
umap_1
umap_2
20
10
0
−10
−5
5
6
4
3
2
1
