# Supplementary material for: Integrative genomic and single-cell transcriptomic analyses suggest a putative zinc transport–immune axis in Meniere’s disease
Source: Front Neurol. 2026 Sep 8;17:1858821. doi: 10.3389/fneur.2026.1858821 (PMC13595996; doi:10.3389/fneur.2026.1858821)
Supplement: Supplementary file 1 [file Data_Sheet_1.ZIP › 2.17重要基因在细胞中的表达分布/3基因气泡图Rplot(1).pdf]

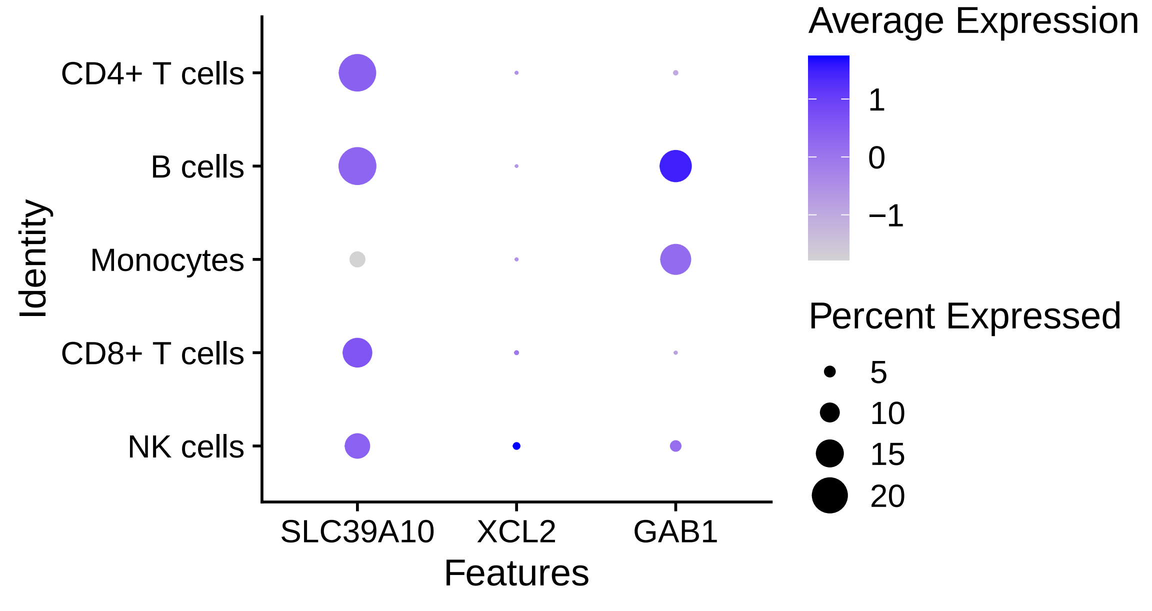
Average Expression
1
0
−1
Percent Expressed
5
10
15
20
Identity
CD4+ T cells
B cells
Monocytes
CD8+ T cells
NK cells
SLC39A10
XCL2
GAB1
Features
