# Supplementary material for: Integrative genomic and single-cell transcriptomic analyses suggest a putative zinc transport–immune axis in Meniere’s disease
Source: Front Neurol. 2026 Sep 8;17:1858821. doi: 10.3389/fneur.2026.1858821 (PMC13595996; doi:10.3389/fneur.2026.1858821)
Supplement: Supplementary file 1 [file Data_Sheet_1.ZIP › 2.1单细胞数据处理/Rplot04.pdf]

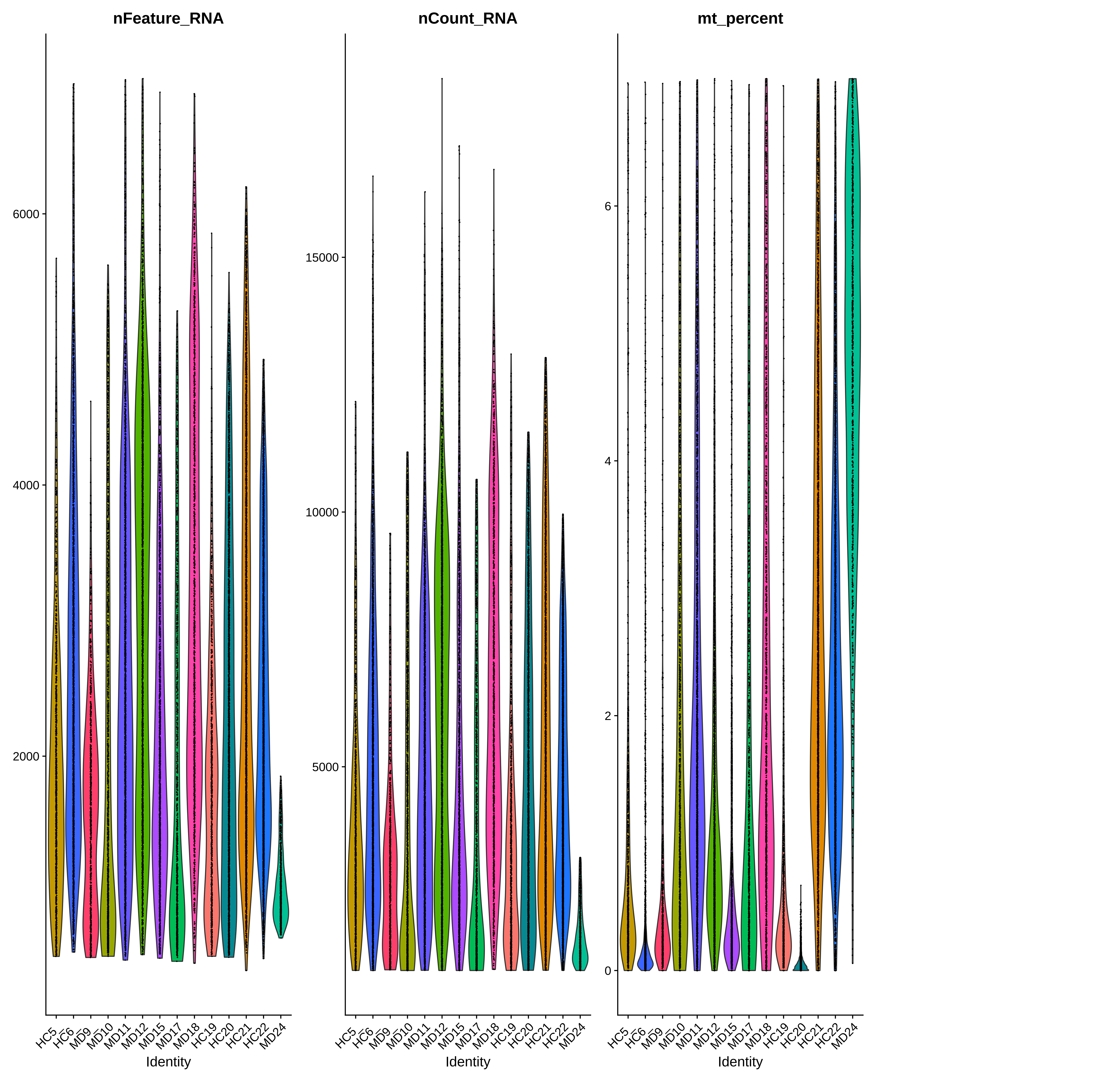
nFeature_RNA
nCount_RNA
mt_percent
6000
4000
2000
15000
10000
5000
6
4
2
0
HC5
HC6
MD9
MD10
MD11
MD12
MD15
MD17
MD18
HC19
HC20
HC21
HC22
MD24
Identity
