# Supplementary material for: Integrative genomic and single-cell transcriptomic analyses suggest a putative zinc transport–immune axis in Meniere’s disease
Source: Front Neurol. 2026 Sep 8;17:1858821. doi: 10.3389/fneur.2026.1858821 (PMC13595996; doi:10.3389/fneur.2026.1858821)
Supplement: Supplementary file 1 [file Data_Sheet_1.ZIP › 2.2细胞聚类分析、可视化和注释/Maker气泡图Rplot.pdf]

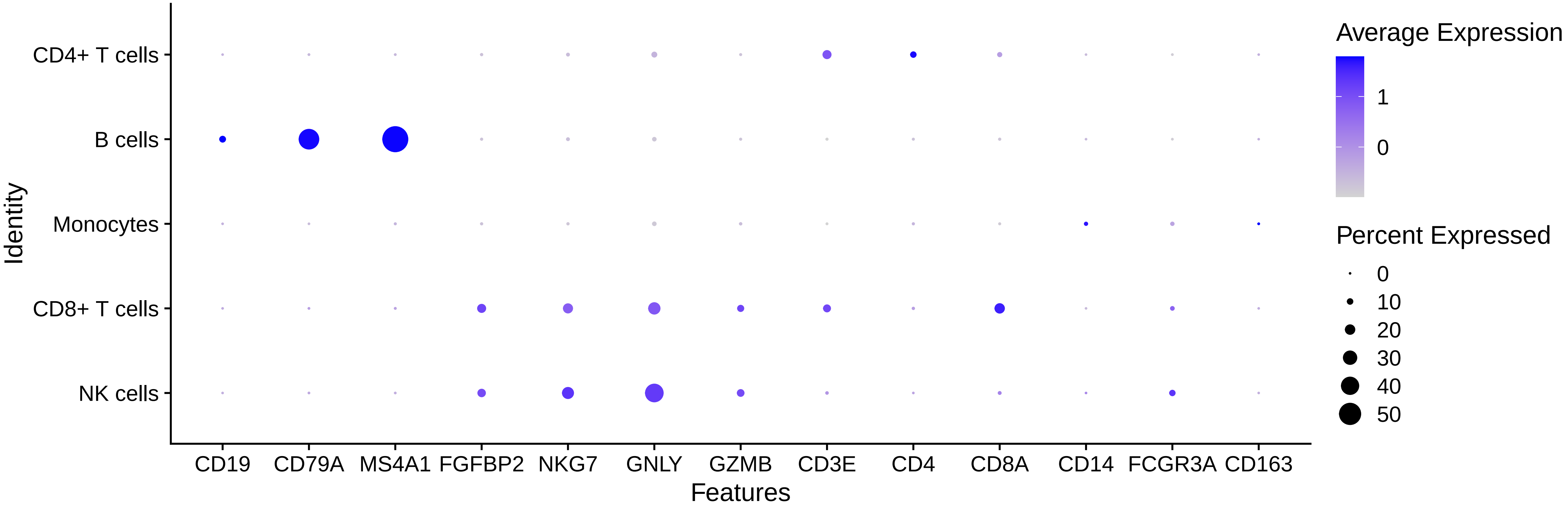

CD4+ T cells
B cells
Monocytes
CD8+ T cells
NK cells
Identity
CD19
CD79A
MS4A1
FGFBP2
NKG7
GNLY
GZMB
CD3E
CD4
CD8A
CD14
FCGR3A
CD163
Features
Average Expression
1
0
Percent Expressed
0
10
20
30
40
50
