# Supplementary material for: Integrative genomic and single-cell transcriptomic analyses suggest a putative zinc transport–immune axis in Meniere’s disease
Source: Front Neurol. 2026 Sep 8;17:1858821. doi: 10.3389/fneur.2026.1858821 (PMC13595996; doi:10.3389/fneur.2026.1858821)
Supplement: Supplementary file 1 [file Data_Sheet_1.ZIP › 2.2细胞聚类分析、可视化和注释/Marker_gene_pointplot.pdf]

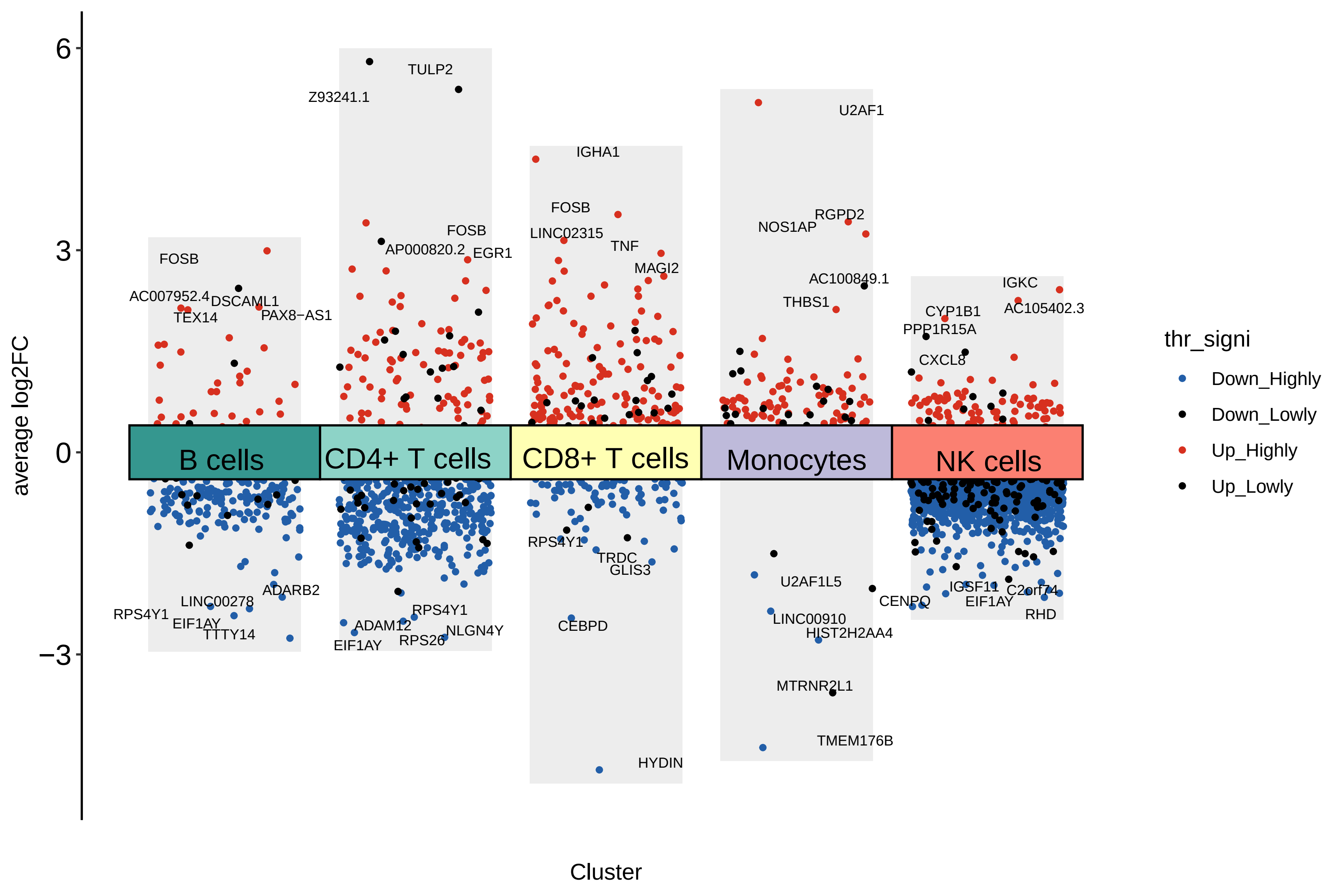

6
3
0
−3
average log2FC
Cluster
B cells
CD4+ T cells
CD8+ T cells
Monocytes
NK cells
FOSB
AC007952.4
DSCAML1
TEX14
PAX8−AS1
RPS4Y1
LINC00278
EIF1AY
TTTY14
ADARB2
TULP2
Z93241.1
FOSB
AP000820.2
EGR1
ADAM12
RPS4Y1
EIF1AY
RPS26
NLGN4Y
IGHA1
FOSB
LINC02315
TNF
MAGI2
RPS4Y1
TRDC
GLIS3
CEBPD
HYDIN
U2AF1
RGPD2
NOS1AP
AC100849.1
THBS1
U2AF1L5
LINC00910
HIST2H2AA4
MTRNR2L1
TMEM176B
IGKC
CYP1B1
AC105402.3
PPP1R15A
CXCL8
CENPQ
IGSF11
C2orf74
EIF1AY
RHD
thr_signi
Down_Highly
Down_Lowly
Up_Highly
Up_Lowly
