# Supplementary material for: Integrative genomic and single-cell transcriptomic analyses suggest a putative zinc transport–immune axis in Meniere’s disease
Source: Front Neurol. 2026 Sep 8;17:1858821. doi: 10.3389/fneur.2026.1858821 (PMC13595996; doi:10.3389/fneur.2026.1858821)
Supplement: Supplementary file 1 [file Data_Sheet_1.ZIP › 2.2细胞聚类分析、可视化和注释/细胞比例图.pdf]

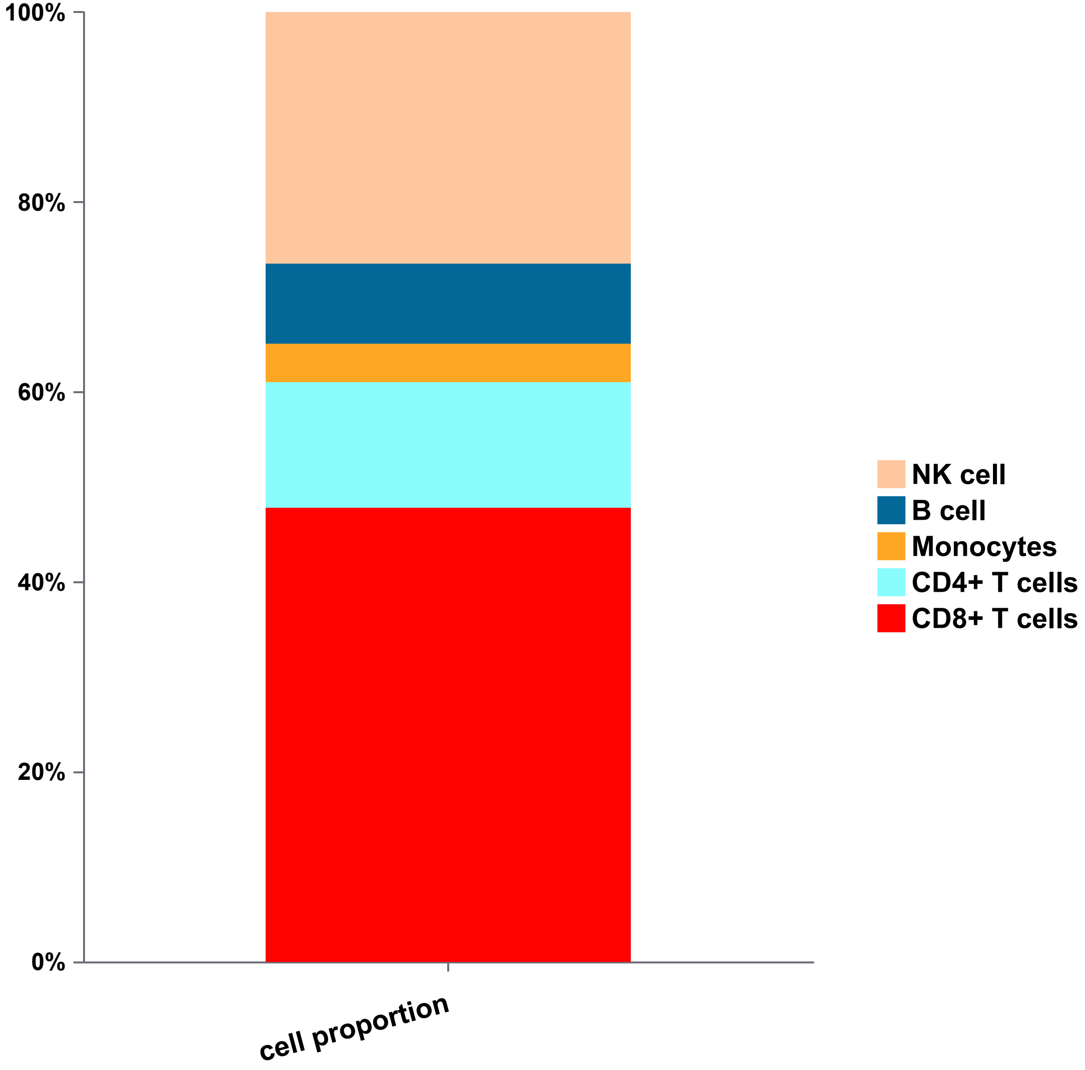
100%
80%
60%
40%
20%
0%
cell proportion
NK cell
B cell
Monocytes
CD4+ T cells
CD8+ T cells
