# Supplementary material for: Integrative genomic and single-cell transcriptomic analyses suggest a putative zinc transport–immune axis in Meniere’s disease
Source: Front Neurol. 2026 Sep 8;17:1858821. doi: 10.3389/fneur.2026.1858821 (PMC13595996; doi:10.3389/fneur.2026.1858821)
Supplement: Supplementary file 1 [file Data_Sheet_1.ZIP › 2.2细胞聚类分析、可视化和注释/聚类.pdf]

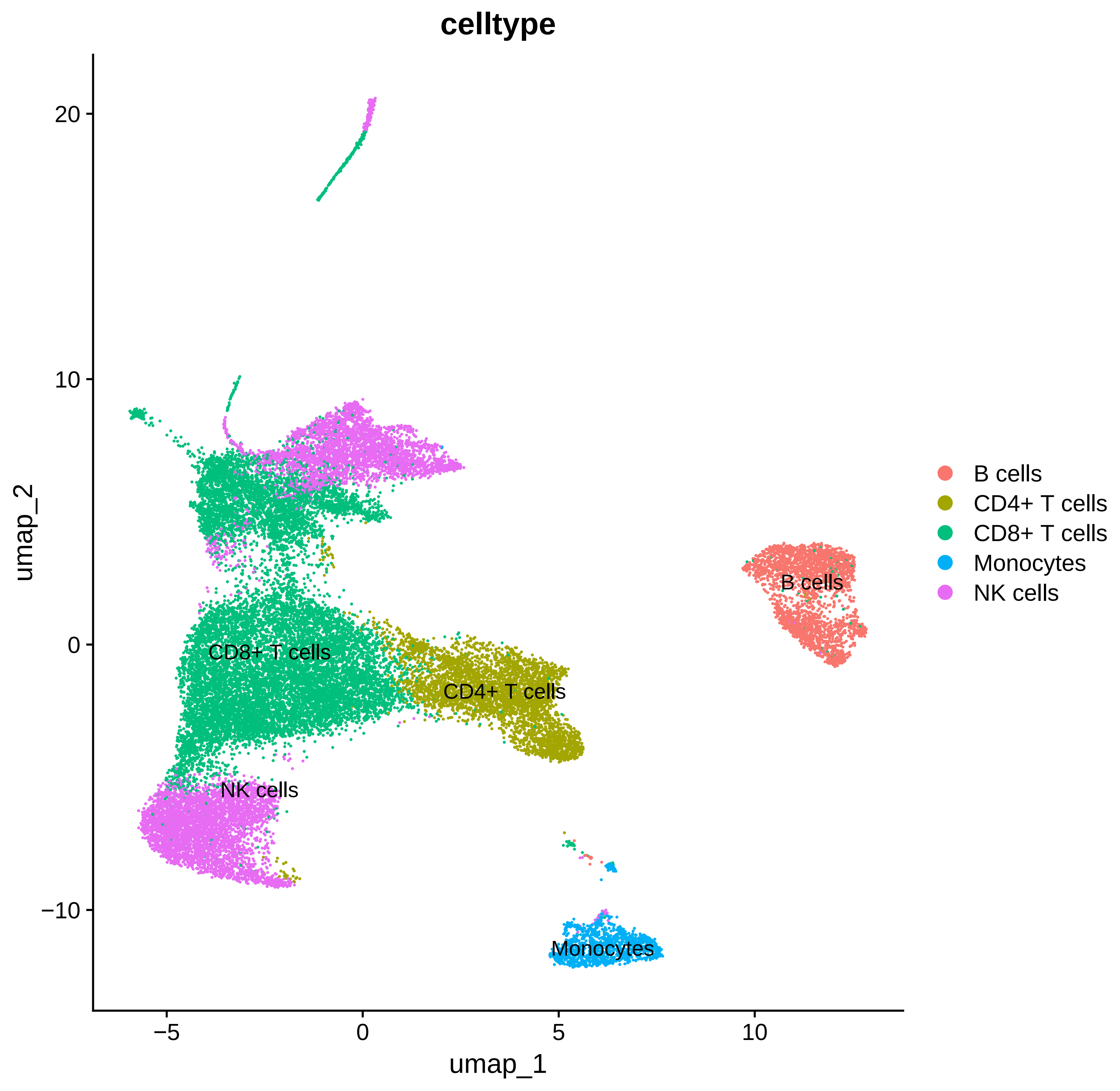
celltype
umap_2
umap_1
20
10
0
−10
−5
0
5
10
CD8+ T cells
CD4+ T cells
NK cells
B cells
Monocytes
B cells
CD4+ T cells
CD8+ T cells
Monocytes
NK cells
