# Supplementary material for: Integrative genomic and single-cell transcriptomic analyses suggest a putative zinc transport–immune axis in Meniere’s disease
Source: Front Neurol. 2026 Sep 8;17:1858821. doi: 10.3389/fneur.2026.1858821 (PMC13595996; doi:10.3389/fneur.2026.1858821)
Supplement: Supplementary file 1 [file Data_Sheet_1.ZIP › 2.3加权基因共表达网络分析结果/3_Scale independence.pdf]

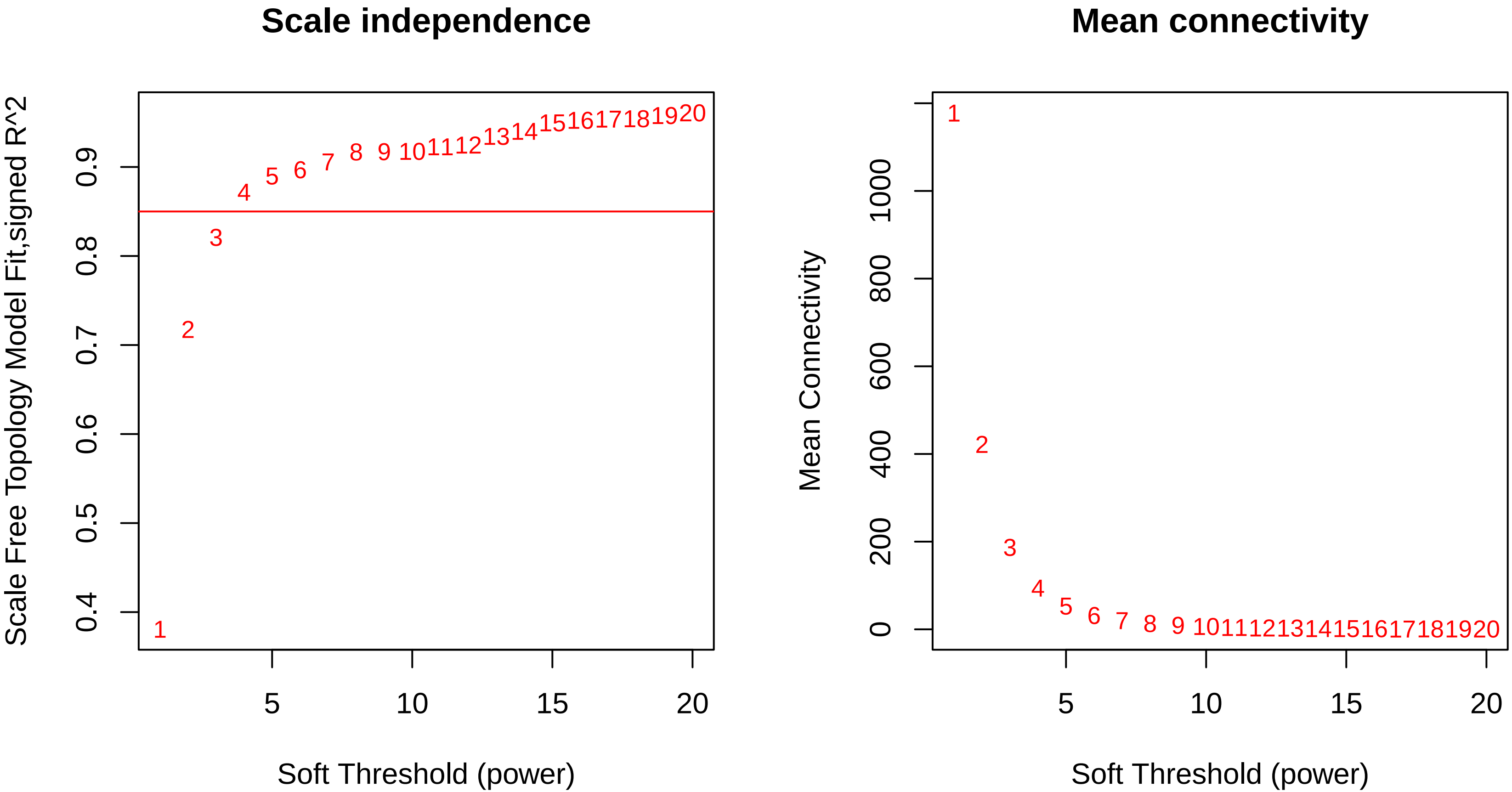
Scale independence
Scale Free Topology Model Fit,signed R^2
0.4
0.5
0.6
0.7
0.8
0.9
1
2
3
4
5
6
7
8
9
10
11
12
13
14
15
16
17
18
19
20
5
10
15
20
Soft Threshold (power)
Mean connectivity
Mean Connectivity
0
200
400
600
800
1000
Soft Threshold (power)
