# Supplementary material for: Integrative genomic and single-cell transcriptomic analyses suggest a putative zinc transport–immune axis in Meniere’s disease
Source: Front Neurol. 2026 Sep 8;17:1858821. doi: 10.3389/fneur.2026.1858821 (PMC13595996; doi:10.3389/fneur.2026.1858821)
Supplement: Supplementary file 1 [file Data_Sheet_1.ZIP › 2.3加权基因共表达网络分析结果/7_merged dynamic.pdf]

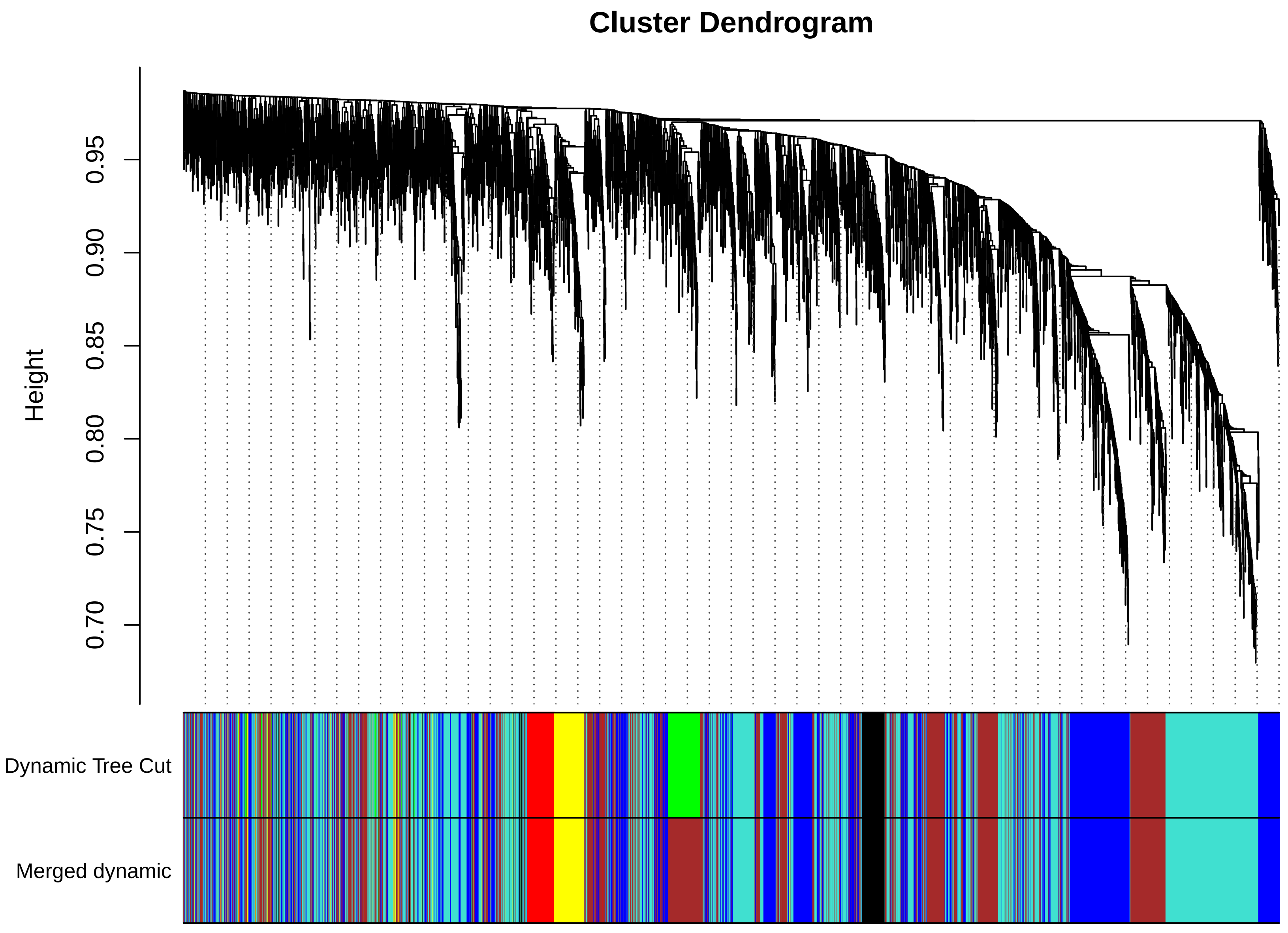
Cluster Dendrogram
Height
0.95
0.90
0.85
0.80
0.75
0.70
Dynamic Tree Cut
Merged dynamic
