# Supplementary material for: Integrative genomic and single-cell transcriptomic analyses suggest a putative zinc transport–immune axis in Meniere’s disease
Source: Front Neurol. 2026 Sep 8;17:1858821. doi: 10.3389/fneur.2026.1858821 (PMC13595996; doi:10.3389/fneur.2026.1858821)
Supplement: Supplementary file 1 [file Data_Sheet_1.ZIP › 2.3加权基因共表达网络分析结果/8_Module-trait relationships.pdf]

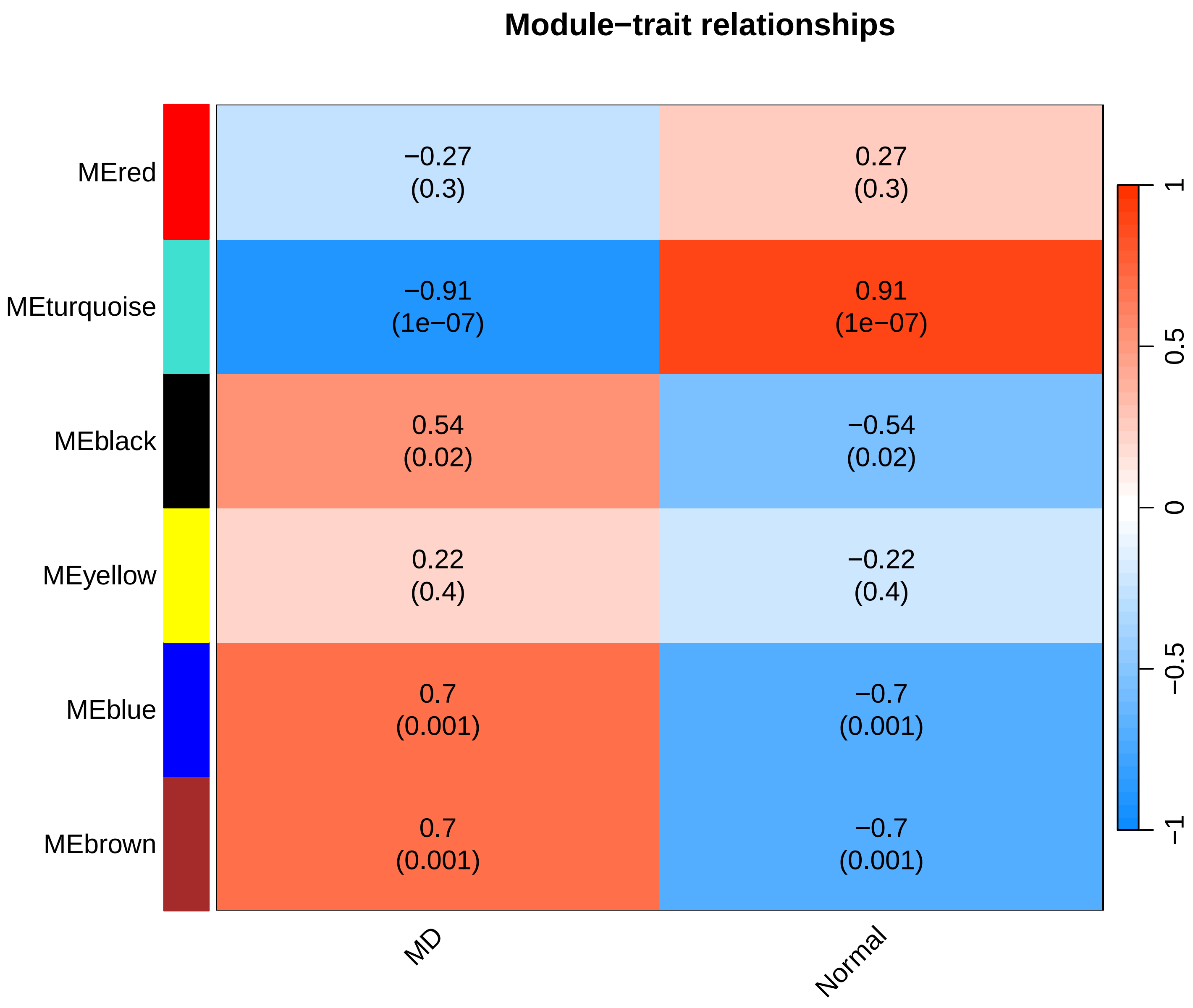
Module−trait relationships
MEred
MEturquoise
MEblack
MEyellow
MEblue
MEbrown
−0.27
(0.3)
0.27
(0.3)
−0.91
(1e−07)
0.91
(1e−07)
0.54
(0.02)
−0.54
(0.02)
0.22
(0.4)
−0.22
(0.4)
0.7
(0.001)
−0.7
(0.001)
0.7
(0.001)
−0.7
(0.001)
MD
Normal
1
0.5
0
−0.5
−1
