# Supplementary material for: Integrative genomic and single-cell transcriptomic analyses suggest a putative zinc transport–immune axis in Meniere’s disease
Source: Front Neurol. 2026 Sep 8;17:1858821. doi: 10.3389/fneur.2026.1858821 (PMC13595996; doi:10.3389/fneur.2026.1858821)
Supplement: Supplementary file 1 [file Data_Sheet_1.ZIP › 2.3加权基因共表达网络分析结果/9_MD_blue_Module membership vs gene significance.pdf]

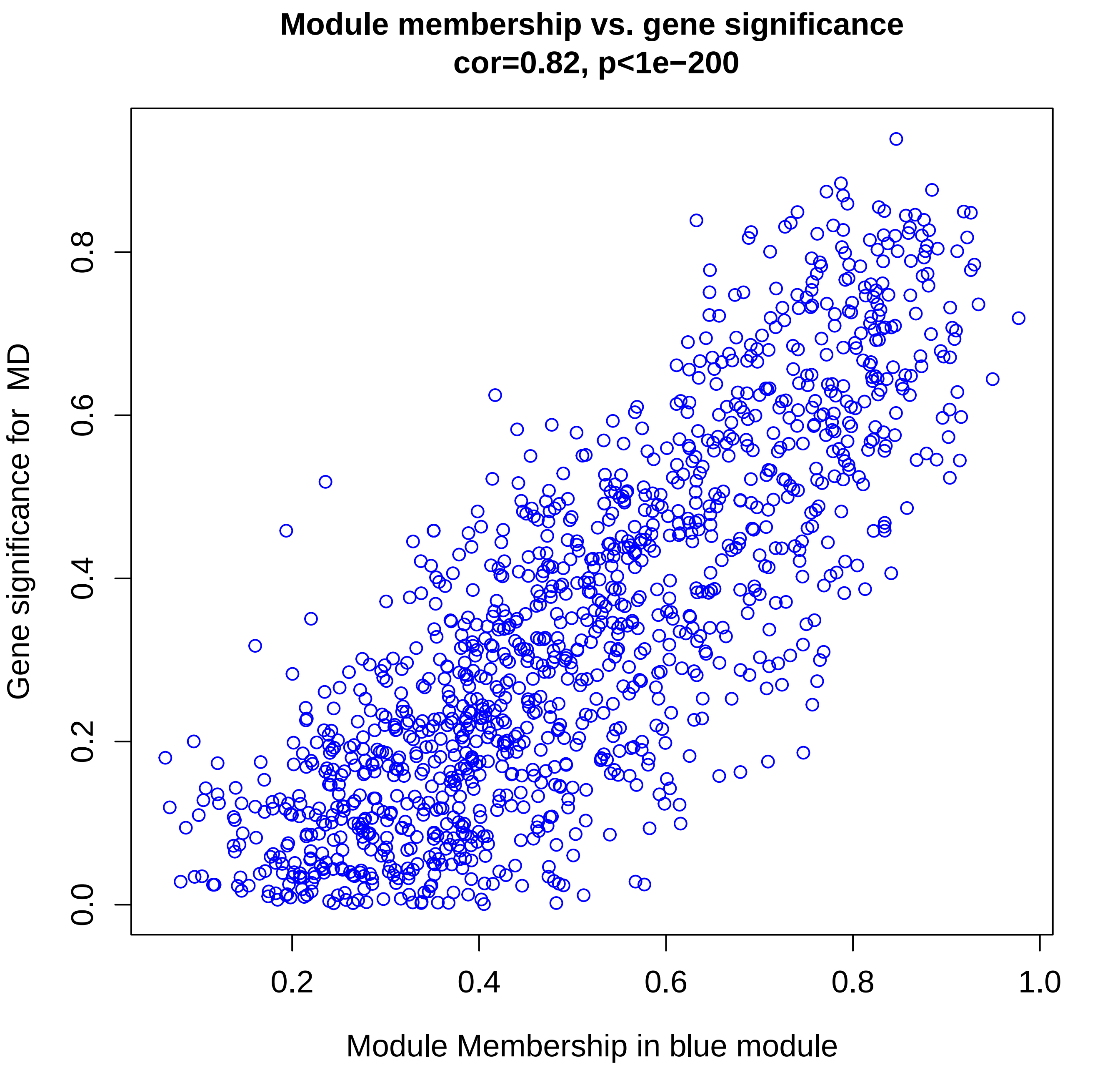

Module membership vs. gene significance
cor=0.82, p<1e−200
Gene significance for MD
0.0
0.2
0.4
0.6
0.8
0.2
0.4
0.6
0.8
1.0
Module Membership in blue module
