# Supplementary material for: Integrative genomic and single-cell transcriptomic analyses suggest a putative zinc transport–immune axis in Meniere’s disease
Source: Front Neurol. 2026 Sep 8;17:1858821. doi: 10.3389/fneur.2026.1858821 (PMC13595996; doi:10.3389/fneur.2026.1858821)
Supplement: Supplementary file 1 [file Data_Sheet_1.ZIP › 2.4基因表达情况分析/GSE202657_DEG.pdf]

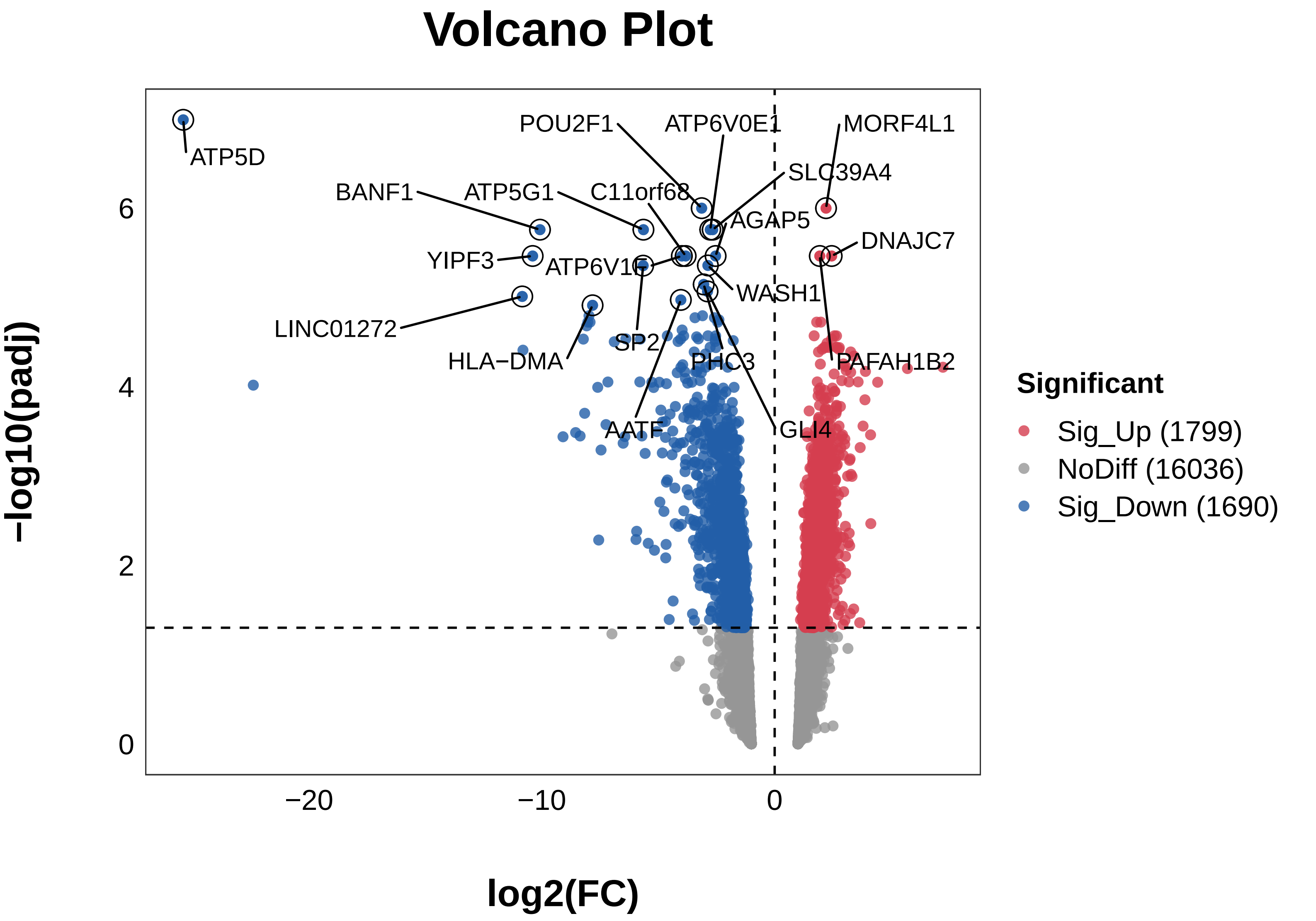
Volcano Plot
ATP5D
POU2F1
ATP6V0E1
MORF4L1
SLC39A4
BANF1
ATP5G1
C11orf68
AGAP5
DNAJC7
YIPF3
ATP6V1F
WASH1
LINC01272
SP2
PHC3
PAFAH1B2
HLA−DMA
AATF
GLI4
6
4
2
0
−20
−10
0
−log10(padj)
log2(FC)
Significant
Sig_Up (1799)
NoDiff (16036)
Sig_Down (1690)
