# Supplementary material for: Integrative genomic and single-cell transcriptomic analyses suggest a putative zinc transport–immune axis in Meniere’s disease
Source: Front Neurol. 2026 Sep 8;17:1858821. doi: 10.3389/fneur.2026.1858821 (PMC13595996; doi:10.3389/fneur.2026.1858821)
Supplement: Supplementary file 1 [file Data_Sheet_1.ZIP › 2.4基因表达情况分析/TOP20GSE202657热图.pdf]

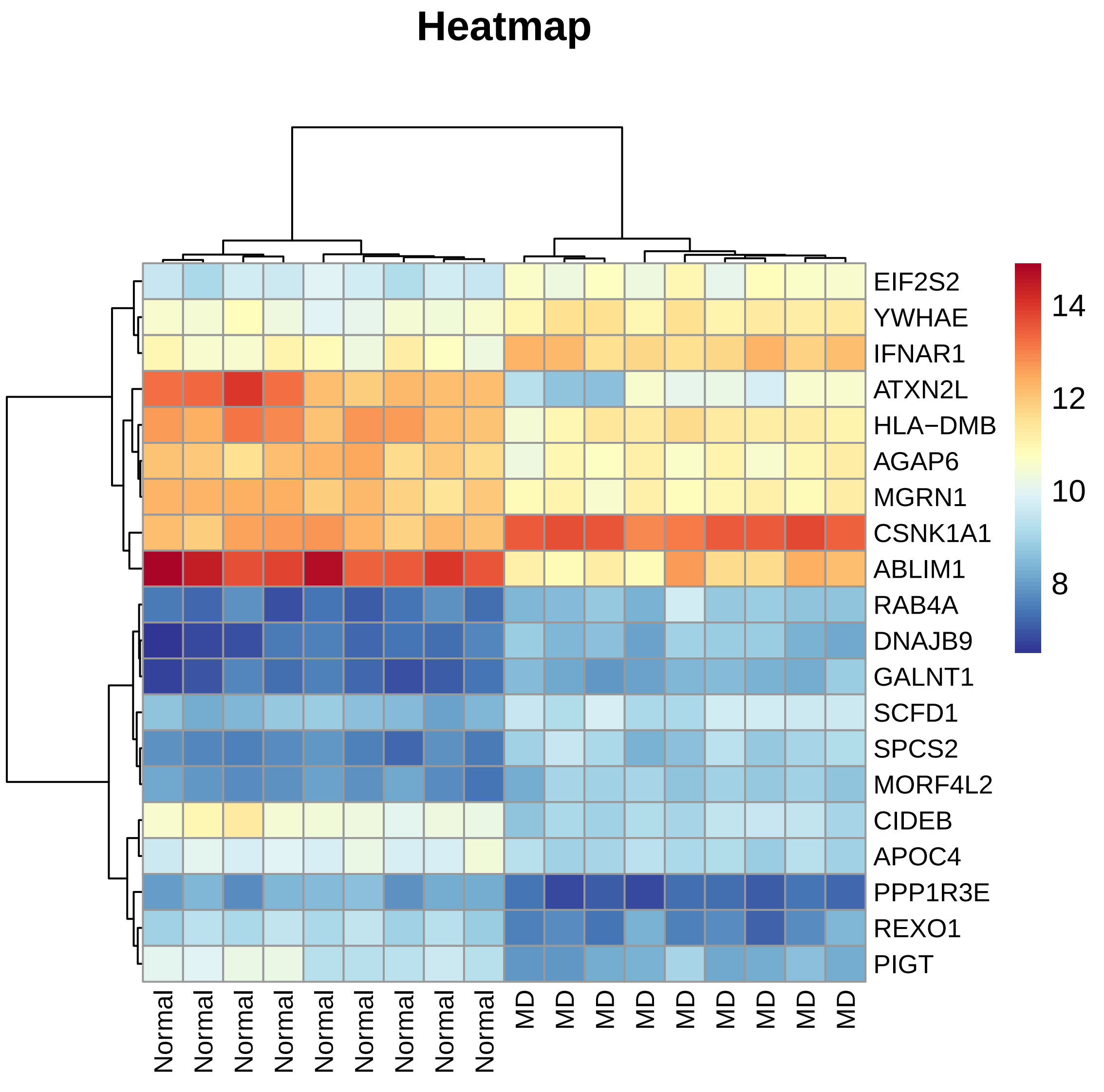

Heatmap
EIF2S2
YWHAE
IFNAR1
ATXN2L
HLA−DMB
AGAP6
MGRN1
CSNK1A1
ABLIM1
RAB4A
DNAJB9
GALNT1
SCFD1
SPCS2
MORF4L2
CIDEB
APOC4
PPP1R3E
REXO1
PIGT
Normal
Normal
Normal
Normal
Normal
Normal
Normal
Normal
Normal
MD
MD
MD
MD
MD
MD
MD
MD
MD
14
12
10
8
