# Supplementary material for: Integrative genomic and single-cell transcriptomic analyses suggest a putative zinc transport–immune axis in Meniere’s disease
Source: Front Neurol. 2026 Sep 8;17:1858821. doi: 10.3389/fneur.2026.1858821 (PMC13595996; doi:10.3389/fneur.2026.1858821)
Supplement: Supplementary file 1 [file Data_Sheet_1.ZIP › 2.6功能富集分析结果/top10GO_result.pdf]

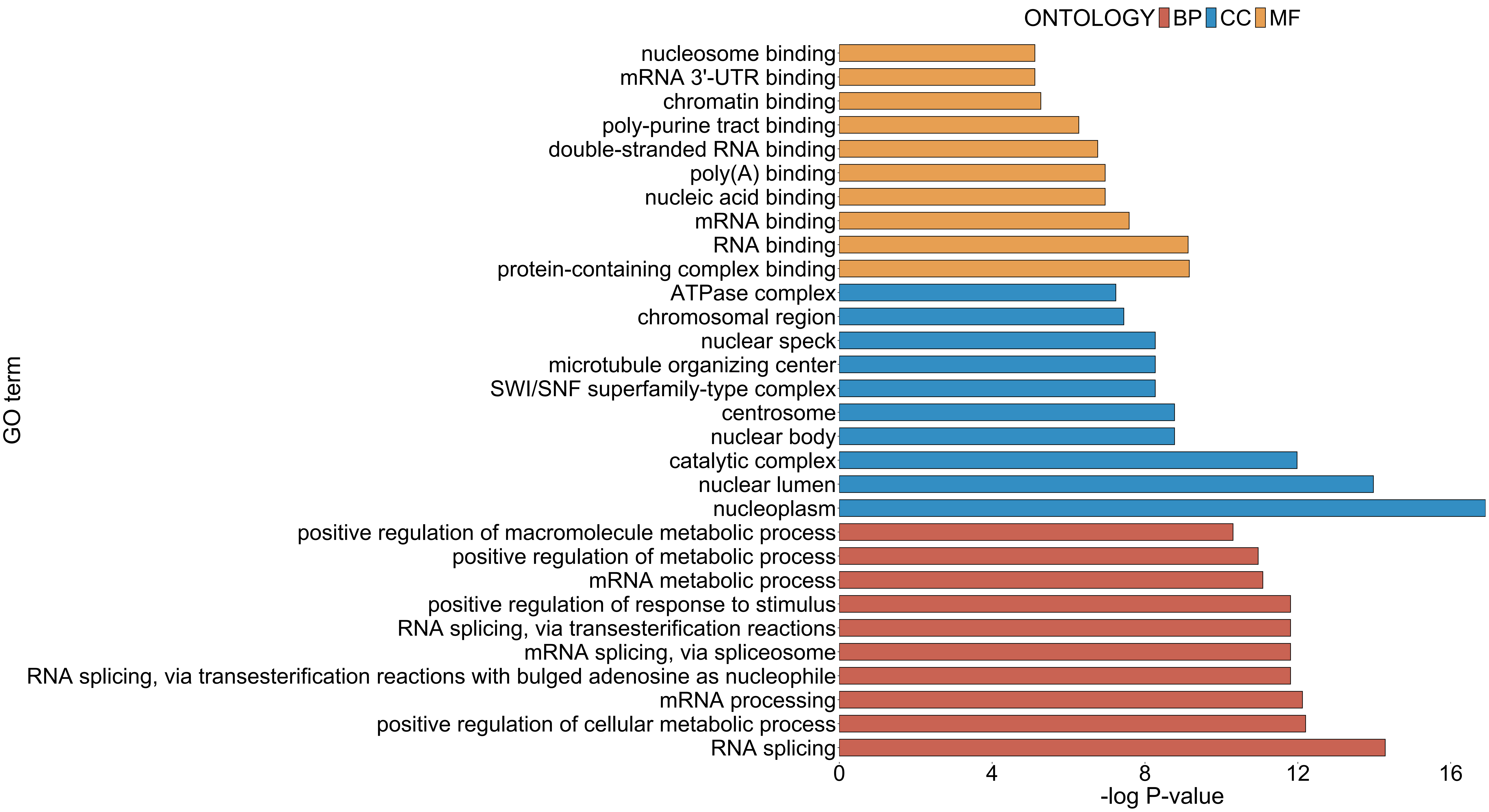

ONTOLOGY
BP
CC
MF
nucleosome binding
mRNA 3'-UTR binding
chromatin binding
poly-purine tract binding
double-stranded RNA binding
poly(A) binding
nucleic acid binding
mRNA binding
RNA binding
protein-containing complex binding
ATPase complex
chromosomal region
nuclear speck
microtubule organizing center
SWI/SNF superfamily-type complex
centrosome
nuclear body
catalytic complex
nuclear lumen
nucleoplasm
positive regulation of macromolecule metabolic process
positive regulation of metabolic process
mRNA metabolic process
positive regulation of response to stimulus
RNA splicing, via transesterification reactions
mRNA splicing, via spliceosome
RNA splicing, via transesterification reactions with bulged adenosine as nucleophile
mRNA processing
positive regulation of cellular metabolic process
RNA splicing
GO term
0
4
8
12
16
-log P-value
