# Supplementary material for: Integrative genomic and single-cell transcriptomic analyses suggest a putative zinc transport–immune axis in Meniere’s disease
Source: Front Neurol. 2026 Sep 8;17:1858821. doi: 10.3389/fneur.2026.1858821 (PMC13595996; doi:10.3389/fneur.2026.1858821)
Supplement: Supplementary file 1 [file Data_Sheet_1.ZIP › 2.6功能富集分析结果/top10KEGG_result.pdf]

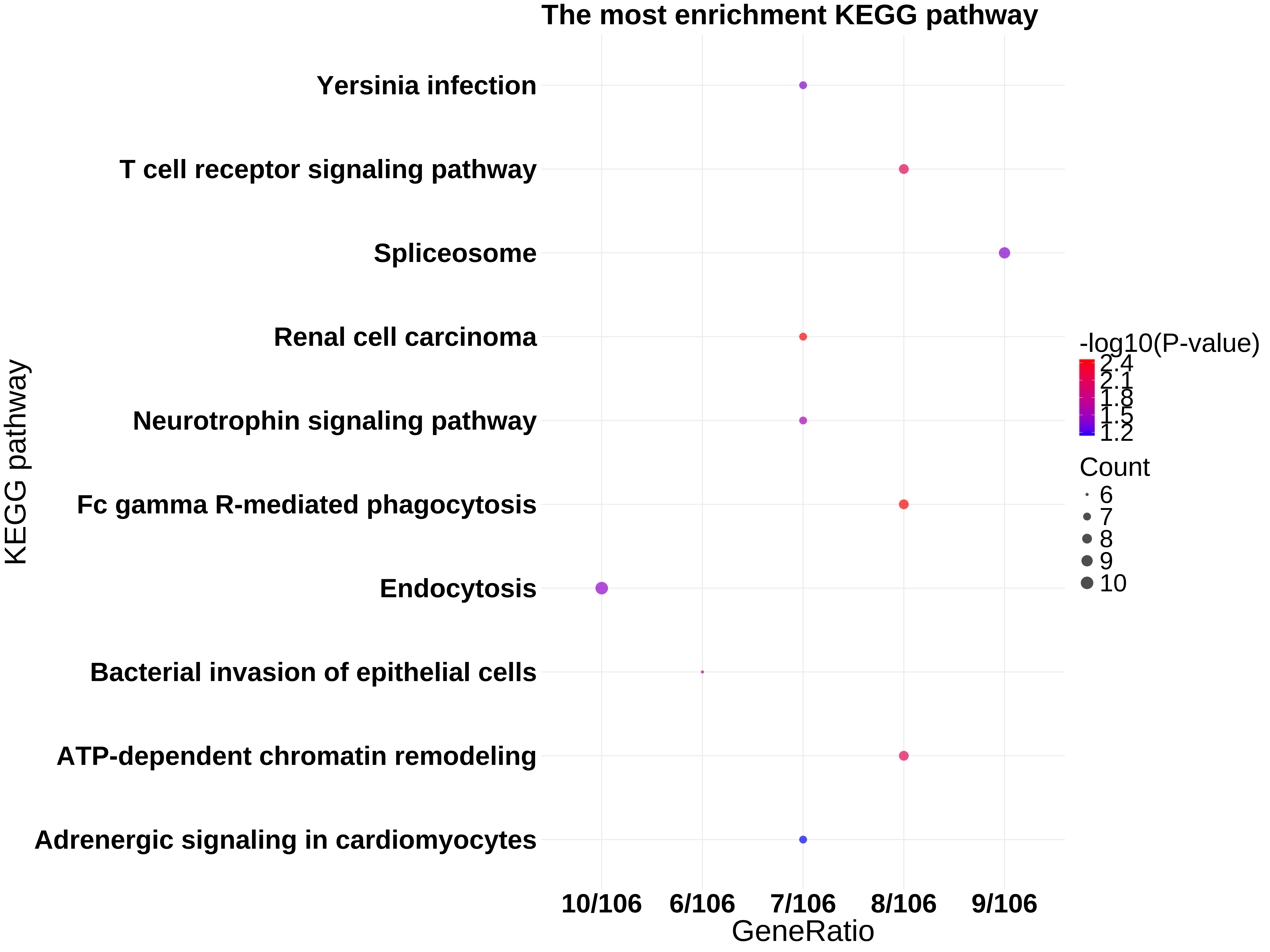

The most enrichment KEGG pathway
Yersinia infection
T cell receptor signaling pathway
Spliceosome
Renal cell carcinoma
Neurotrophin signaling pathway
Fc gamma R-mediated phagocytosis
Endocytosis
Bacterial invasion of epithelial cells
ATP-dependent chromatin remodeling
Adrenergic signaling in cardiomyocytes
KEGG pathway
10/106
6/106
7/106
8/106
9/106
GeneRatio
-log10(P-value)
2.4
2.1
1.8
1.5
1.2
Count
6
7
8
9
10
