# Supplementary material for: Integrative genomic and single-cell transcriptomic analyses suggest a putative zinc transport–immune axis in Meniere’s disease
Source: Front Neurol. 2026 Sep 8;17:1858821. doi: 10.3389/fneur.2026.1858821 (PMC13595996; doi:10.3389/fneur.2026.1858821)

Outcome	Exposure	method	nsnp	OR(95%CI)	pval
MD	LRRFIP1	Inverse variance weighted	14	0.56(0.36 to 0.88)	0.02773829
MD	SLC39A10	Inverse variance weighted	19	1.79(1.08 to 2.95)	0.03738301
MD	XCL2	Inverse variance weighted	18	0.85(0.74 to 0.98)	0.04296238
MD	GAB1	Inverse variance weighted	2	0.42(0.19 to 0.92)	0.02906060

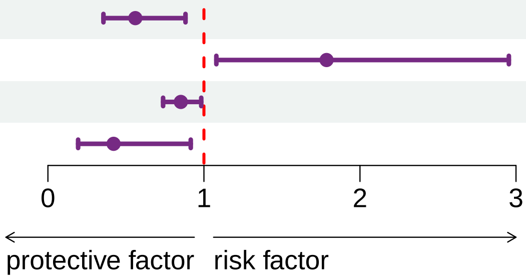

Supplement: Supplementary file 1 [file Data_Sheet_1.ZIP › 2.7孟德尔随机化/森林图.pdf]
