# Supplementary material for: Integrative genomic and single-cell transcriptomic analyses suggest a putative zinc transport–immune axis in Meniere’s disease
Source: Front Neurol. 2026 Sep 8;17:1858821. doi: 10.3389/fneur.2026.1858821 (PMC13595996; doi:10.3389/fneur.2026.1858821)
Supplement: Supplementary file 1 [file Data_Sheet_1.ZIP › 2.9重要基因确认/GAB1.pdf]

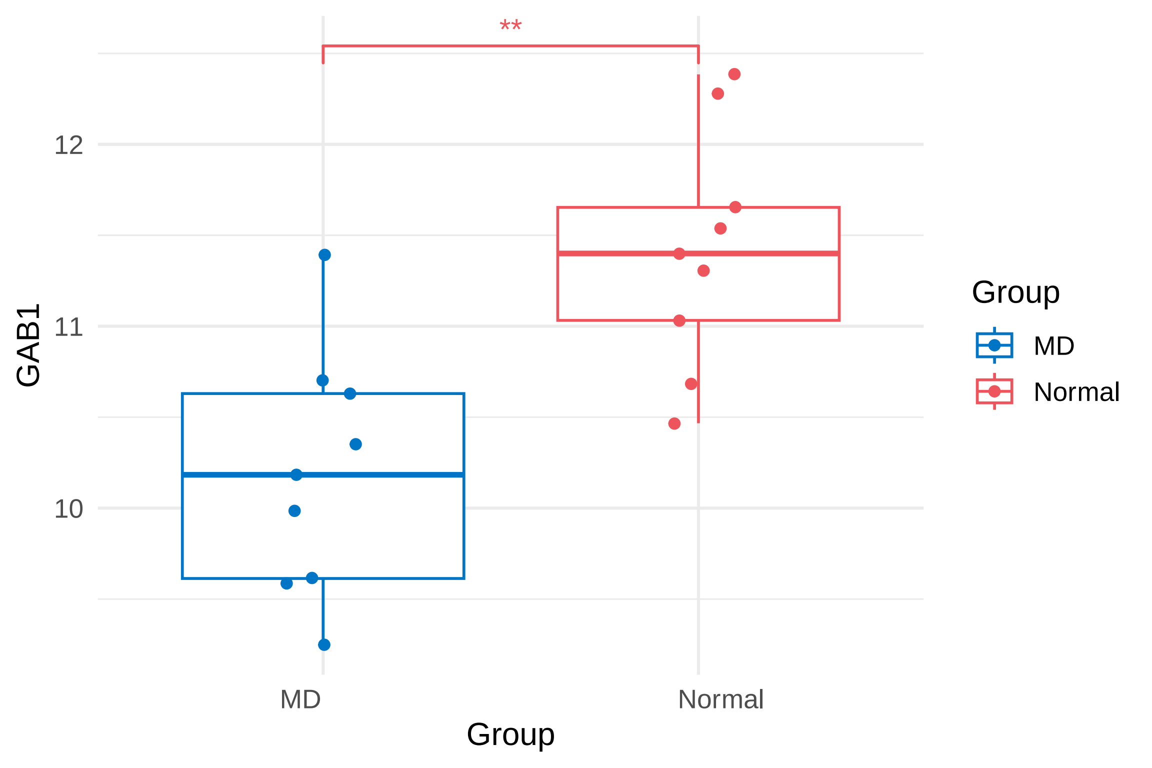

**
12
11
10
GAB1
MD
Normal
Group
Group
MD
Normal
